# Oral microbiome and lesion-associated microbiological findings in medication-related osteonecrosis of the jaw: a systematic review and evidence map

**DOI:** 10.3389/fcimb.2026.1923000

**Published:** 2026-09-08

**Authors:** Xinrui Yin, Shijia Du, Jun Wan

**Affiliations:** 1 Department of Anesthesiology, Aerospace Center Hospital, Beijing, China; 2 Department of VIP Dental Service, Peking University Stomatological Hospital, Beijing, China; 3 Department of Stomatology, The First Affiliated Hospital of Dalian Medical University, Dalian, China

**Keywords:** *Actinomyces*, anaerobic bacteria, biofilm, evidence map, medication-related osteonecrosis of the jaw, oral microbiome, systematic review

## Abstract

**Background:**

Medication-related osteonecrosis of the jaw (MRONJ) may involve microbial colonization, anaerobic organisms, and biofilm formation, but whether it has a reproducible microbial signature remains unclear. We mapped the human microbiological evidence in MRONJ.

**Methods:**

The protocol was registered in PROSPERO (CRD420261432571). Reporting followed PRISMA 2020, PRISMA-S, and Synthesis Without Meta-analysis guidance. Six sources were searched from inception in two waves through 25 July 2026, supplemented by citation searching. Original human microbiological studies of MRONJ were eligible. Community-level evidence was classified as core, other MRONJ microbiological evidence as supportive, and evidence from related jawbone conditions as contextual. Five prespecified domains included explicit MRONJ-specific *Actinomyces* events and denominators, strict-anaerobe evidence, and direct biofilm evidence. Design-specific JBI checklists and STORMS-informed reporting items were applied. Meta-analysis was conditional on prespecified compatibility criteria.

**Results:**

Of 4,044 records, 71 reports representing 67 independent studies were included: 51 MRONJ studies, comprising 13 core and 38 supportive studies, and 16 contextual studies. Community-level findings were not sufficiently comparable for pooling. Among the 51 MRONJ studies, criteria were met by 19 for strict-anaerobe evidence, seven for viral evidence, six for explicit *Actinomyces* events and denominators, three for direct biofilm evidence, and two for fungal evidence; 32 contributed to at least one domain. Nine method-specific *Actinomyces* observations from six studies reported raw detection proportions of 5.1%–65.7% by culture, 82.9%–96.4% by PCR or quantitative PCR, and 36.4%–100.0% by histology, using patient- or specimen-level denominators. Diagnostic criteria, antibiotic timing, negative controls, and data availability were inconsistently reported. No domain met the compatibility criteria for meta-analysis.

**Conclusions:**

The MRONJ microbiological evidence is methodologically heterogeneous and dominated by observational, lesion-derived studies. Detection proportions varied substantially with method and denominator unit, limiting their interpretation as prevalence estimates. Repeated reports of anaerobic organisms and *Actinomyces*, together with sparse direct biofilm evidence, describe lesion-associated findings of undetermined specificity rather than a reproducible MRONJ-specific signature. Standardized comparators and sampling, explicit denominators, contamination controls, extractable numerical results, and accessible sequence data are needed for future synthesis and clinical evaluation.

**Systematic review registration:**

https://www.crd.york.ac.uk/prospero/display_record.php?ID=CRD420261432571, identifier CRD420261432571.

## 1 Introduction

Medication-related osteonecrosis of the jaw (MRONJ) is an adverse condition associated with antiresorptive, antiangiogenic, and other bone-modifying therapies, occurring most frequently in patients treated with bisphosphonates or denosumab for malignancy-associated bone disease or osteoporosis (Yarom et al., 2019; Ruggiero et al., 2022). It is defined clinically by exposed or probeable necrotic bone in the maxillofacial region persisting beyond a specified interval in patients with relevant drug exposure and without a history of jaw radiotherapy or metastatic disease involving the jaws, and it may be accompanied by pain, suppuration, delayed healing, and impaired oral function (Yarom et al., 2019; Ruggiero et al., 2022). Despite successive refinements of diagnostic and staging criteria, MRONJ remains difficult to prevent and to treat, and its pathogenesis is generally regarded as multifactorial, involving suppressed bone remodeling, impaired angiogenesis, local trauma, mucosal breakdown, immune dysregulation, and infection (Yarom et al., 2019; Ruggiero et al., 2022).

The microbiological component of this model has attracted sustained attention because the jaws are anatomically adjacent to the densely colonized oral cavity and are frequently affected by dentoalveolar trauma and mucosal disruption (McLean, 2014; Baker et al., 2024). Once mucosal integrity is lost and necrotic bone becomes accessible to the oral environment, colonization, anaerobic proliferation, and biofilm formation may plausibly sustain inflammation and impede healing (Donlan and Costerton, 2002; Hall-Stoodley et al., 2004; Sedghizadeh et al., 2008; Ruggiero et al., 2022; Cui et al., 2025). Human studies have accordingly examined MRONJ specimens using culture, histology and special staining, targeted PCR and quantitative PCR, 16S rRNA gene sequencing, shotgun metagenomics, and biofilm-directed microscopy, and have repeatedly reported *Actinomyces*, anaerobic bacteria, and polymicrobial aggregates in necrotic bone and lesion-associated tissue (Sedghizadeh et al., 2008; Sedghizadeh et al., 2012; Pushalkar et al., 2014; Russmueller et al., 2016; Hallmer et al., 2017; Panya et al., 2017; Brody et al., 2022). Whether these observations reflect a disease-associated microbial signature, secondary colonization of devitalized tissue, or the way in which such specimens are obtained and examined remains unresolved.

Three features make this literature particularly difficult to interpret and cannot be resolved by statistical pooling alone. First, community-level profiling and targeted organism detection answer different questions: the former characterizes composition or diversity relative to a comparator, whereas the latter documents whether a prespecified organism or feature was detected in the sampled material (Russmueller et al., 2016; Hallmer et al., 2017; Panya et al., 2017; Brody et al., 2022). Counting them together introduces a systematic asymmetry, because a study designed to detect a given organism is far more likely to report it than a community-wide survey, so that simple study counts may reflect investigator intent and selective testing as well as underlying microbial occurrence. Second, detection proportions are reported using inconsistent denominators—patients, specimens, lesions, or isolates—and often derive from patients or specimens selected because surgical or histological material was available, so that numerically similar proportions may not be comparable and numerically different proportions may not indicate biological discordance (Russmueller et al., 2016; Panya et al., 2017; Brody et al., 2022). Third, terms such as *biofilm* and *anaerobe* are applied inconsistently, ranging from direct visualization of organized microbial communities to inference from the recovery of multiple taxa, and correspondingly heterogeneous claims cannot be aggregated without explicit operational definitions (Donlan and Costerton, 2002; Hall-Stoodley et al., 2004; Sedghizadeh et al., 2008).

Microbiological findings in MRONJ also overlap with those described in other necrotic or chronically inflamed jawbone conditions, notably osteoradionecrosis and jaw osteomyelitis (Hansen et al., 2006; Goda et al., 2014). These conditions differ from MRONJ in etiology and diagnostic criteria and cannot substitute for MRONJ populations, but the literature concerning them delineates the boundary of what may be attributed specifically to drug-associated osteonecrosis (Hansen et al., 2006; Goda et al., 2014; Yarom et al., 2019; Ruggiero et al., 2022). Reports of these conditions, and reports in which MRONJ and other jaw pathologies could not be separated, were therefore retained here as explicitly labeled contextual evidence used to delimit the reach of MRONJ-specific inference rather than as comparators for analysis.

Accordingly, this systematic review and evidence map had four objectives: to map the human microbiological evidence in MRONJ, separating core community-level evidence from supportive microbiological evidence and from contextual evidence in related jawbone conditions; to characterize how consistently clinical, pharmacological, and sampling variables relevant to microbial interpretation have been reported; to appraise methodological conduct at item level and the reporting completeness of core microbiome studies against STORMS-informed criteria (Mirzayi et al., 2021); and to determine, against prespecified compatibility criteria, whether any evidence domain was sufficiently comparable for meta-analysis, and what would be required to make such synthesis possible.

## 2 Methods

### 2.1 Protocol registration and reporting standards

This systematic review and evidence map was designed to synthesize human evidence on oral microbial findings in medication-related osteonecrosis of the jaw (MRONJ). The protocol was prospectively registered in PROSPERO on 24 June 2026 (CRD420261432571). The registered review question was unchanged during the conduct of the review; the manuscript title was refined to reflect the final structure of the evidence base. The review was conducted and reported in accordance with the Preferred Reporting Items for Systematic Reviews and Meta-Analyses (PRISMA) 2020 statement (Page et al., 2021), with the literature search reported in accordance with PRISMA-S (Rethlefsen et al., 2021) and the narrative, non-meta-analytic synthesis reported in accordance with the Synthesis Without Meta-analysis guidance (Campbell et al., 2020).

MRONJ was the primary condition of interest. Osteoradionecrosis of the jaw and jaw osteomyelitis were retained as contextual conditions in order to delimit the boundary of MRONJ-specific inference, that is, to indicate where the available evidence did not permit conclusions specific to drug-associated osteonecrosis. They were not treated as co-primary disease populations, were labeled separately throughout, and did not contribute to MRONJ-specific analyses, denominators, or conclusions.

The synthesis was planned as a structured narrative synthesis and evidence map. Meta-analysis was planned conditionally and was to be undertaken only where studies were sufficiently comparable with respect to population, design, comparator, outcome definition, detection method, and denominator unit.

### 2.2 Eligibility criteria

Eligible reports were original human studies reporting microbiome or lesion-associated microbiological findings in patients with MRONJ, including studies using the historical terms bisphosphonate-related, antiresorptive-related, and drug-related osteonecrosis of the jaw. Eligible specimens comprised oral, mucosal, salivary, lesion-associated, jawbone, necrotic-bone, and surgical samples obtained from the target condition. No minimum sample-size threshold was applied. Case reports and case series were eligible when they contained original human microbiological findings, but were classified as core community-level evidence only if they provided an eligible community-level analysis.

Participants were required to have MRONJ or an equivalent historical diagnosis as defined by the original investigators. We recorded the diagnostic criteria applied, whether a minimum duration of exposed or probeable bone was specified, and whether previous jaw radiotherapy and metastatic disease involving the jaw were excluded (Yarom et al., 2019; Ruggiero et al., 2022). Elements not reported by the original investigators were coded as not reported rather than assumed to have been satisfied.

Community-level or untargeted profiling constituted core evidence when an eligible MRONJ-specific diversity, community-composition, or differential-abundance result was available. Culture, targeted PCR or quantitative PCR, histology or special staining, microscopy, *in situ* hybridization, biofilm-specific methods, organism-specific detection, and fungal or viral findings constituted supportive microbiological evidence. Evidence role was assigned according to the type of eligible result available for synthesis rather than the laboratory platform alone; a study using a community-profiling platform was therefore classified as supportive when no eligible community-level diversity or composition result could be separately extracted.

For mixed disease populations, only separately extractable MRONJ findings contributed to the MRONJ-specific synthesis. Original human studies of osteoradionecrosis, jaw osteomyelitis, or non-separable mixed jaw-disease populations were retained as separately labeled contextual evidence when they reported lesion-associated microbiological findings, and did not contribute to MRONJ-specific denominators or conclusions.

Reviews, systematic reviews, guidelines, editorials, comments, conference abstracts and other abstract-only reports, non-human or *in vitro*–only studies, treatment-only reports, imaging-only studies, risk-factor studies, host-biomarker studies without original microbiological findings, studies of non-eligible anatomical sites, and reports without an eligible original microbiological result were excluded. No language restriction was applied.

### 2.3 Information sources and search strategy

The review database was assembled in two waves: an initial search on 25 June 2026 and a definitive search using the final, expanded strategies on 25 July 2026, both covering PubMed/MEDLINE, Embase, Scopus, Web of Science Core Collection, the Cochrane Library, and ClinicalTrials.gov. Records from both waves were consolidated, deduplicated within and across waves, and screened as a single set under the final eligibility criteria. Bibliographic databases were searched from inception without date, language, or publication-type restrictions, and controlled vocabulary was combined with database-specific free-text terms where available.

For each bibliographic database, an MRONJ disease branch and a contextual jaw-disease branch were combined with corresponding microbiology term blocks, and the two branches were joined with OR. ClinicalTrials.gov was searched using a disease-condition query without an additional microbiology block, in order to identify registered, ongoing, or potentially unpublished studies. Backward reference-list screening and forward citation searching were used as supplementary identification methods.

The exact executed queries, platforms, search dates, restrictions, and yields for each source and wave are provided in **Supplementary Table 1**. The strategies underwent internal structural verification and known-item recall testing, but were not formally peer-reviewed by an external information specialist (Rethlefsen et al., 2021).

### 2.4 Study selection and full-text retrieval

Records from both search waves were consolidated into a single database and deduplicated using DOI, PMID, and standardized title matching, with uncertain matches reviewed manually. The consolidated set was screened against the final eligibility criteria; screening decisions were based solely on those criteria, and no priority, likelihood, or evidence-level designation was used to determine eligibility.

Two reviewers independently screened titles and abstracts and independently assessed retrieved full texts. Records that could not be clearly excluded at title and abstract screening proceeded to full-text retrieval. Disagreements were resolved by consensus between the two reviewers. Records identified through citation searching were processed through a separate identification pathway.

Full texts were sought through DOI and publisher links, PubMed and PubMed Central, database-provided links, open repositories, local project holdings, and available institutional-access routes. Reports that remained unavailable after repeated retrieval attempts were documented separately, were excluded from the full-text-assessed denominator, and were not assigned a scientific exclusion reason.

### 2.5 Data extraction and study clustering

The extraction form captured study design, disease terminology and diagnostic criteria, sample sizes and denominator units, underlying treatment indication, medication class and route, treatment duration, MRONJ stage, lesion location, recent antibiotic exposure and its timing, oral hygiene, periodontal disease, previous extraction or dental surgery, previous debridement, smoking, immunosuppression, specimen timing and site, microbiological method, comparator structure, and result-level microbiological findings.

Two reviewers independently extracted all data and resolved discrepancies by consensus. Data were extracted from the main text, tables, figures, and **Supplementary Materials** at report level, and harmonized for synthesis at independent-study level. Information absent from a report was coded as not reported, incomplete or non-separable information as unclear, and genuinely non-applicable fields as not applicable. Missing values were not imputed.

Potentially overlapping reports were assessed using authorship, institution, recruitment period, sample size, participant characteristics, microbiological methods, and registration information where available. Companion reports were linked to the same independent-study cluster and could supplement non-duplicated information, but did not generate an additional study-level observation. Patients, specimens, lesions, isolates, appraisal units, and event and denominator data were counted only once within any synthesis domain.

### 2.6 Outcomes and evidence classification

Community-level outcomes comprised alpha-diversity metrics, beta-diversity analyses, differential-abundance findings, and other explicitly reported community-composition results (Knight et al., 2018). Alpha-diversity richness measures were distinguished from diversity and evenness measures, and paired and independent comparisons, as well as MRONJ-versus-comparator and within-MRONJ comparisons, were treated as separate analytical structures. Community-level profiling approaches were classified into mutually exclusive categories according to the technique used to generate taxonomic data: high-throughput 16S rRNA amplicon sequencing, community fingerprinting by denaturing gradient gel electrophoresis with clone-library sequencing, and shotgun metagenomic sequencing. Studies combining a community-level approach with culture were classified by their community-level component.

Organism- and domain-specific outcomes comprised five prespecified categories: explicit MRONJ-specific *Actinomyces* events and denominators, strict-anaerobe evidence, direct biofilm evidence, fungal evidence, and viral evidence. A taxon was coded as detected only when an eligible MRONJ result explicitly reported its presence, isolation, detection, or abundance. Assay target lists, background and discussion statements, comparator-only findings, and non-separable mixed-population findings did not establish detection in MRONJ. Enrichment or depletion required an explicit eligible comparison with an interpretable between-group result; presence in an uncontrolled lesion sample was not coded as enrichment.

Direct biofilm evidence required direct visualization, or a method explicitly used to demonstrate biofilm architecture or organization, including scanning electron microscopy, confocal microscopy, or spatially resolved *in situ* visualization (Donlan and Costerton, 2002; Hall-Stoodley et al., 2004). Strict-anaerobe evidence required anaerobic culture with an eligible result, an organism explicitly classified as anaerobic in the source report, or molecular detection of named obligate anaerobic taxa; detection of *Actinomyces* alone did not establish strict-anaerobe evidence (Könönen and Wade, 2015). Fungal and viral evidence required an explicit eligible MRONJ-specific result obtained by culture, histology, molecular detection, *in situ* hybridization, metagenomic analysis, or another clearly reported method.

For evidence mapping, result states were defined separately according to result type. Non-comparative detection results were coded as detected, not detected, unclear, or not reported. Comparative results were coded as enriched, depleted, no difference, mixed or unclear, or not reported, with “no difference” applied only when an eligible comparative analysis explicitly reported no statistically significant difference or otherwise supported a clear conclusion that no meaningful between-group difference was present. Domain-level results were coded as criterion met, criterion not met, unclear, or not reported. Throughout, “unclear” indicated that the reported information could not support an unambiguous classification, and “not reported” that no eligible result was located. Where a study reported several results within the same outcome domain, such as multiple diversity indices, comparator groups, specimen types, or detection methods, all eligible results were recorded separately and were not averaged or reduced to a single representative value.

### 2.7 Critical appraisal and microbiome reporting assessment

Each independent study was appraised using the JBI critical appraisal checklist corresponding to its design: analytical cross-sectional, case-control, cohort, case series, or case report (Barker et al., 2023). Companion reports could supply additional information for an item within the same study but did not create an additional appraisal unit. Judgments were recorded item by item as yes, no, unclear, or not applicable. No total score, percentage threshold, or overall category of methodological concern was derived, because the design-specific checklists assess different domains and contain different numbers of items.

Studies classified as core community-level evidence were additionally assessed against STORMS-informed reporting items (Mirzayi et al., 2021), categorized as reported, not reported, unclear, or not applicable. The operational definition of each item is provided in **Supplementary Table 5**. This assessment characterized reporting status and was not treated as a risk-of-bias assessment. Two reviewers independently completed the critical appraisal and the reporting assessment, with disagreements resolved by consensus.

### 2.8 Synthesis methods

Evidence was grouped as core community-level, supportive microbiological, or contextual, and organized by specimen, comparator, microbiological method, denominator unit, and outcome domain. Synthesis comprised study-level narrative description, structured tabulation, and result-level evidence mapping (Campbell et al., 2020).

Pooling of alpha-diversity results required compatible metrics, comparable populations and comparator structures, consistent paired or independent designs, and extractable group-specific means, standard deviations, and sample sizes. Beta-diversity and differential-abundance findings required a common interpretable effect measure and compatible analytical definitions before quantitative combination would be considered.

*Actinomyces* observations were eligible for descriptive result-level display only when they were MRONJ-specific, provided explicit events and denominators, identified the detection method and denominator unit, and did not use a denominator selected on the basis of positivity. Raw study-specific proportions were calculated as events divided by the corresponding denominator. Patient-, specimen-, lesion-, and isolate-level denominators were kept separate, and culture, histology, PCR or quantitative PCR, and sequencing results were not combined across methods.

Meta-analysis was to be undertaken only if studies addressed a sufficiently common estimand and were compatible with respect to the clinical population, study design, comparator structure, specimen type, sampling and testing strategy, outcome definition, detection method, denominator unit, cohort independence, and availability of extractable data; the assessment of feasibility against these criteria is presented with the corresponding results and summarized in **Table 1**. Summary statistics were used only as reported. Medians and interquartile ranges were not converted to means and standard deviations, values were not digitized from figures, and missing variance data were not imputed, because the underlying distributions, comparator structures, and denominator units were not sufficiently comparable to justify conversion. As no meta-analysis or pooled quantitative synthesis was performed, statistical exploration of heterogeneity was not applicable; methodological and clinical heterogeneity was instead addressed *a priori* by stratifying evidence according to evidence role, specimen type, detection method, comparator structure, and denominator unit. Data management, tabulation, calculation of raw proportions, and figure preparation were performed in R version 4.6.0 (R Core Team, Vienna, Austria) using the readr, dplyr, tidyr, janitor, gtsummary, ggplot2, ComplexUpset, and pheatmap packages (Giorgi et al., 2022).

**Table 1:** Summary of microbiological evidence and quantitative-synthesis feasibility.

Evidence domain	Independent studies or result-level observations	Evidence type	Main descriptive finding	Principal compatibility limitation	Synthesis approach
Community-level microbiome profiling	13 independent studies	Community-level profiles	Community profiles were reported with study-specific specimens and methods.	Design, specimen, comparator, and outcome heterogeneity.	Narrative synthesis
Alpha diversity	8 studies; 6 with explicit MRONJ-versus-comparator analyses	Richness and diversity/evenness metrics	Findings were retained separately by metric, comparator structure, and sampling design.	Metrics, paired status, summaries, and comparators were incompatible.	Not quantitatively pooled
*Actinomyces*	9 result-level observations from 6 independent studies	Method-specific event/denominator data	Method-specific event/denominator findings were summarized separately by detection method and denominator unit.	Small strata; mixed patient/specimen units; selected and targeted methods.	Method-specific descriptive display
Direct biofilm	3 independent studies	Direct or validated biofilm evidence	Direct biofilm evidence was distinguished from presumed or discussion-only biofilm.	Visualization and validation methods were not uniform.	Evidence-map synthesis
Strict anaerobic evidence	19 independent MRONJ studies	Direct anaerobic evidence or named obligate-anaerobe findings	Strict evidence required a qualifying anaerobic culture, explicit author classification, or named obligate anaerobes.	Culture and molecular evidence were method-specific.	Evidence-map synthesis
Fungal evidence	2 independent studies	Explicit fungal finding	Fungal findings were reported separately from bacterial and viral observations.	Limited and heterogeneous study-level reporting.	Evidence-map synthesis
Viral evidence	7 independent studies	Explicit viral finding	Viral findings were retained as explicit result-level evidence.	Limited target-specific and case-based observations.	Evidence-map synthesis
Contextual jaw-disease evidence	16 independent studies	Contextual community-level or lesion-associated evidence	Contextual jaw-disease findings were separated from MRONJ-specific synthesis.	Different diseases and clinical ecologies; excluded from MRONJ-specific counts.	Narrative synthesis

MRONJ, medication-related osteonecrosis of the jaw.

### 2.9 Implementation of the prespecified synthesis plan

Meta-analysis was conditional on adequate clinical and methodological comparability. No outcome domain met the prespecified compatibility criteria; the review was therefore completed using structured narrative synthesis, evidence mapping, and method- and denominator-stratified descriptive displays, and no pooled estimate or heterogeneity statistic was generated. No other substantive departure from the registered protocol occurred.

Certainty-of-evidence grading was not applied because the synthesis did not yield a sufficiently standardized outcome or effect estimate to which a common certainty judgment could be meaningfully assigned.

## 3 Results

### 3.1 Study selection

Searches conducted across the two waves identified 4,044 records from the six sources. After removal of 2,312 duplicate records within and across waves, 1,732 unique records were screened under the final eligibility criteria.

Title and abstract screening excluded 1,422 records, and 310 unique reports were sought for retrieval in the database and registry pathway. Of these, 293 were obtained and 17 remained unavailable, a retrieval rate of 94.5%. Full-text assessment resulted in the inclusion of 68 reports and the exclusion of 225. The most frequent reasons for exclusion were absence of an original microbiological result (n = 53), insufficient information to establish an eligible original microbiological finding (n = 49), an ineligible disease condition (n = 38), a focus restricted to treatment or risk factors (n = 29), and non-original, abstract-only, protocol, or registry publication status (n = 29). No eligible report was identified from ClinicalTrials.gov. Citation searching identified four additional reports, all of which were retrieved and assessed; three were included and one excluded. Reasons for full-text exclusion are reported by category in **Supplementary Table 2**, together with the list of reports that could not be retrieved.

**Figure 1:**
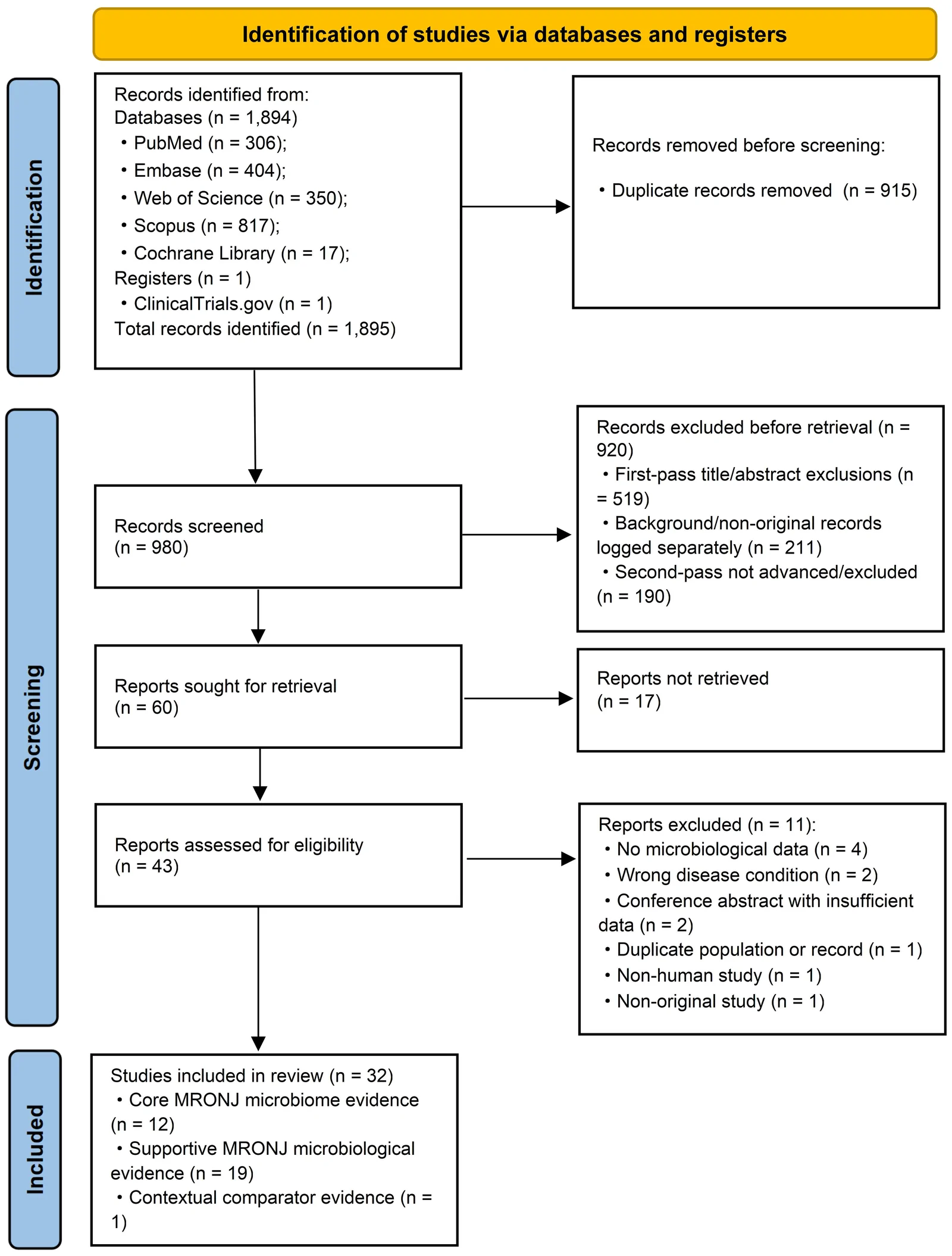
PRISMA 2020 flow diagram of study identification, screening, and inclusion. Searches were conducted in two waves (25 June 2026 and 25 July 2026), and records from the two waves were consolidated and deduplicated before screening. Reports that could not be retrieved were documented separately and were not assigned a scientific exclusion reason. Per-database and per-wave record counts are provided in **Supplementary Table 1**, and reasons for exclusion at full-text assessment are listed report by report in **Supplementary Table 2**.

The final evidence base comprised 71 reports representing 67 independent studies, of which 67 were primary reports and four were companion reports linked to existing study clusters. Independent studies, rather than individual reports, were used as the unit for study-level characterization, critical appraisal, and synthesis. The selection process is shown in **Figure 1**.

### 3.2 Characteristics and structure of the included evidence

The 67 independent studies were published between 1998 and 2026, with a median publication year of 2018. Fifty-one studies addressed MRONJ and were published between 2006 and 2026. The evidence base comprised 13 core community-level studies, 38 supportive microbiological studies, and 16 contextual studies. Eight contextual studies addressed osteoradionecrosis and eight addressed jaw osteomyelitis; two of the 16 studies enrolled mixed-etiology populations. Contextual studies were analyzed separately and did not contribute to MRONJ-specific clinical-reporting or microbiological evidence counts.

Country or region was coded only where explicitly reported, and was available for 52 studies representing 19 countries or regions. The United States contributed the largest number of studies (n = 12), followed by Germany (n = 8), Japan (n = 6), Italy (n = 4), and Hungary (n = 3). Overall, 16 studies used an analytical cross-sectional design, seven were case-control studies, 12 were cohort studies, 20 were case series, and 12 were case reports. The core evidence comprised five analytical cross-sectional studies, seven case-control studies, and one cohort study. The supportive evidence comprised eight analytical cross-sectional studies, seven cohort studies, 12 case series, and 11 case reports, and the contextual evidence comprised three analytical cross-sectional studies, four cohort studies, eight case series, and one case report.

Bone or necrotic-bone specimens predominated across the evidence base (n = 44), followed by lesion or tissue specimens (n = 8), studies in which the specimen was not adequately reported (n = 7), oral or saliva specimens (n = 5), and mixed oral or jaw specimens (n = 3). Mixed microbiological methods formed the principal method category in 21 studies, followed by histological or microscopic methods in 14, community-level profiling in 13, culture-based microbiology in 12, and targeted molecular detection in seven.

Community-level profiling was the principal method category in 13 studies, comprising 11 core studies, one supportive study, and one contextual study. Two further core studies were categorized as mixed-methods studies but incorporated community-level profiling, giving 13 core community-level studies in total. Within this core set, nine studies used high-throughput 16S rRNA amplicon sequencing, three used denaturing gradient gel electrophoresis with clone-library sequencing, and one used shotgun metagenomic sequencing. Comparator structures comprised healthy controls in seven studies, medication-exposed controls without MRONJ in four, disease controls in four, and an unaffected within-patient site in one; these categories were not mutually exclusive, and one study had no independent comparator.

**Figure 2:**
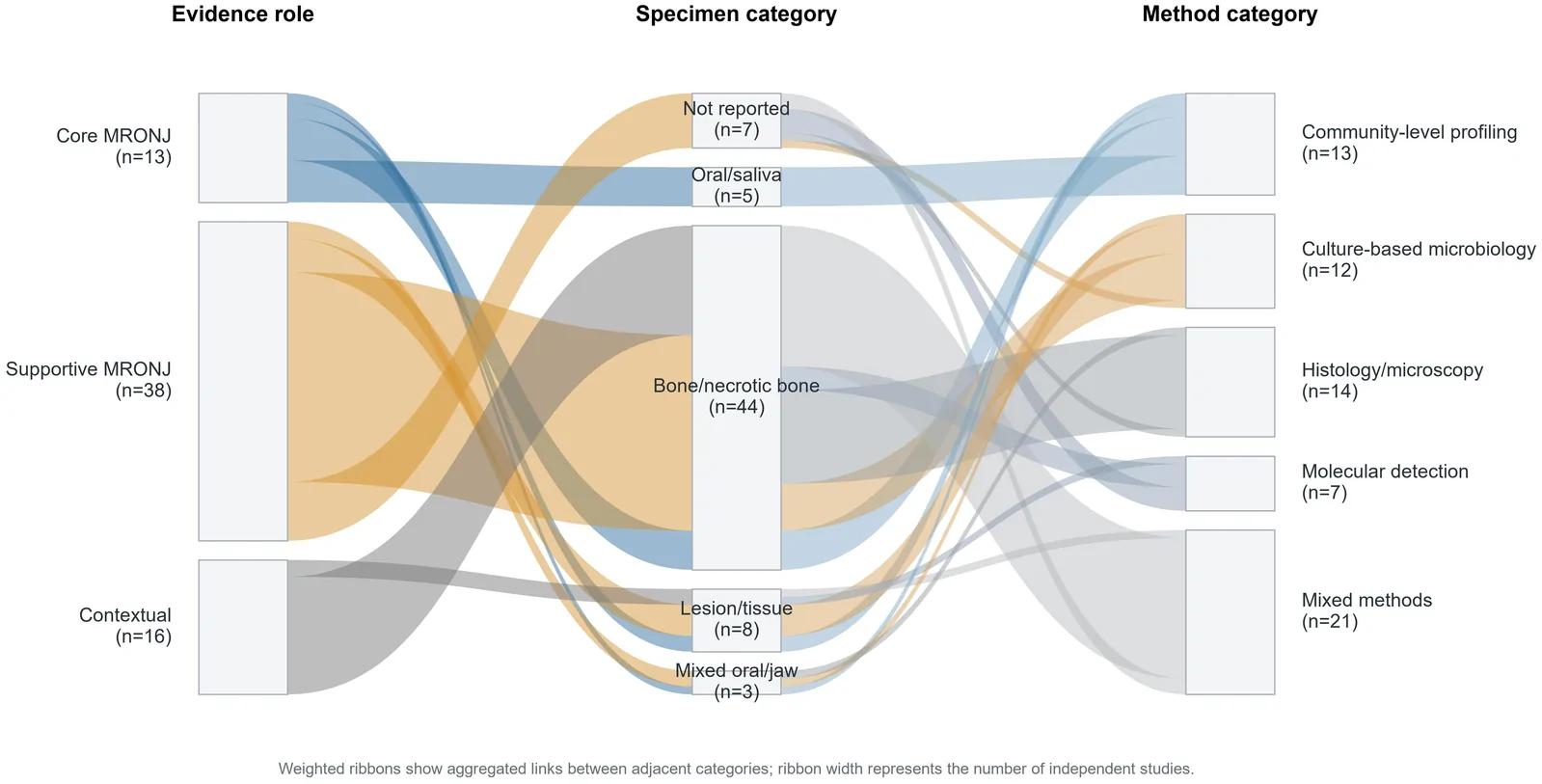
Evidence landscape of included independent studies by specimen category and microbiological method. The diagram maps 67 independent studies across the final specimen and microbiological-method categories, comprising 13 core, 38 supportive, and 16 contextual studies. Weighted ribbons show aggregated links between adjacent categories; ribbon width represents the number of independent studies. Categories were assigned using the final manual classification audit. Mixed methods were assigned only when a study contained two or more independent method domains.

The relationships between evidence role, specimen category, and microbiological method are shown in **Figure 2**. Characteristics of the core community-level studies are presented in **Table 2**, and report-level characteristics and independent-study clustering for the complete evidence base are provided in **Supplementary Table 3**.

**Table 2:** Characteristics of the core community-level MRONJ studies.

Study, country and design	MRONJ and comparator sample sizes	Diagnostic definition	Indication and medication exposure	MRONJ stage	Antibiotic exposure	Specimen and sampling	Community-level method
Yahara H 2020; Japan; Analytical cross-sectional	Initial comparison: 5 MRONJ metagenomic datasets and 5 healthy-control metagenomic datasets; extended analysis: 10 MRONJ and 14 healthy-control datasets.	NR	Cancer-associated MRONJ; antiresorptive exposure; route NR	NR	NR	Unstimulated saliva; 1 mL	Shotgun metagenomic sequencing; Illumina HiSeq 2 × 150 bp
Badros AZ 2021; United States; Cohort	14 patients with BRONJ; 28 control participants (microbiome profiling)	NR	Multiple myeloma; bisphosphonate therapy; route NR	NR	NR	Whole unstimulated saliva; serial sampling	16S rRNA gene sequencing; 97% OTU clustering
Ji 2012; United States; Analytical cross-sectional	20 patients; 10 antibiotic-treated versus 10 non-antibiotic BRONJ patients; no healthy comparator	historical BRONJ definition	Bisphosphonate exposure; intravenous (n=14) and oral (n=6)	Stage 0–2	10 received systemic antibiotics; 10 had no prior antibiotics; treated patients were sampled during a 2-week course	soft tissue associated with BRONJ lesion	16S rRNA DGGE fingerprinting with clone-library sequencing (ABI PRISM 3730xl)
Kim HY 2024; South Korea; Analytical cross-sectional	12 patients; 12 unaffected-site paired samples	partial definition	Osteoporosis (n=11) and multiple myeloma (n=1); medication class/route NR	NR	No antibiotics within 1 week before sampling	oral swab from affected and unaffected sides; submucosal/exudate around lesion	16S rRNA sequencing; V3–V4; Illumina MiSeq; 2 × 300 bp
Li Q 2020; China; Case-control	9 patients; 9 healthy/control oral mucosa specimens	NR	Osteoporosis (n=1) and cancer (n=8); medication class/route NR	Stage II/III	No antibiotics within 1 month before sampling	fresh inflammatory tissue next to necrotic bone or sequestrum	Mixed 16S sequencing and culture methods; V3–V4; Illumina MiSeq, paired-end 250 bp
Choi 2025; South Korea; Case-control	39 patients with MRONJ; 45 healthy participants	NR	Osteoporosis; bisphosphonate and denosumab; route partly reported	NR	NR	sequestrum/MRONJ-related oral microbial samples	16S rRNA sequencing; Illumina HiSeq 2500
Sedghizadeh 2012; United States; Case-control	5 patients with BRONJ; 5 non-BRONJ control participants	historical BRONJ definition	Osteoporosis and prostate cancer; oral or intravenous bisphosphonate exposure	NR	No local or systemic antimicrobials within 6 months before sampling	saliva and saliva-induced biofilms	16S rRNA V3–V5 amplicon sequencing; 454 GS FLX Titanium pyrosequencing; separate viral metagenomic component
Pushalkar 2014; United States; Case-control	15 patients with BRONJ; 10 periodontal control participants and 5 bisphosphonate-exposed participants without ONJ	historical BRONJ definition	Cancer; bisphosphonate exposure; route NR	NR	No antibiotics for approximately 3 months before sampling	BRONJ lesion tissue specimens	16S rRNA DGGE fingerprinting with clone-library sequencing
Kalyan 2015; Germany; Analytical cross-sectional	BAONJ subgroup size not clearly extractable; participants exposed to nitrogen-containing bisphosphonates without ONJ and treatment-naive controls	NR	Bisphosphonate exposure; indication and route NR	NR	No active antibiotic treatment at sampling	oral microbiome samples; exact oral site unclear	16S rRNA sequencing; 454 pyrosequencing
Wei 2012; United States; Case-control	6 patients with BRONJ; 6 cancer control participants without BRONJ or bisphosphonate exposure but with infection	NR	Cancer; intravenous bisphosphonate exposure	NR	No antibiotics at the time of sampling	jawbone samples	16S rRNA DGGE fingerprinting with clone-library sequencing
Erovigni FM 2026; Italy; Case-control	18 patients with MRONJ; 21 drug-exposed control participants without MRONJ; 16 healthy participants	NR	Mixed indications; bisphosphonate and denosumab exposure	NR	Antibiotics started 2 days before surgical sampling	saliva	16S rRNA sequencing; multiple amplicon regions; microbiome panel/NGS
Kövér Z 2025; Hungary; Case-control	35 patients with MRONJ; 35 healthy extraction controls; 5 + 5 specimens selected for 16S	partial definition	Malignancy with bone metastases; bisphosphonate/denosumab; predominantly intravenous	NR	No antibiotics for at least 2 months before primary sampling; initiated subsequently	surgically removed bone samples	Mixed 16S sequencing and culture methods; V3–V4; Illumina MiSeq
Hallmer 2017; Sweden; Analytical cross-sectional	18 necrotic bone samples; 11 visually healthy bone samples; 2 healthy control participants	NR	Mixed indications; medication exposure NR	NR	No antibiotics within 3 months before study sampling	necrotic bone center and visually healthy bone	16S rRNA molecular profiling; V3–V5; 454 GS Junior pyrosequencing

BAONJ, bisphosphonate-associated osteonecrosis of the jaw; BRONJ, bisphosphonate-related osteonecrosis of the jaw; DGGE, denaturing gradient gel electrophoresis; MRONJ, medication-related osteonecrosis of the jaw; NGS, next-generation sequencing; NR, not reported; ONJ, osteonecrosis of the jaw; OTU, operational taxonomic unit.

### 3.3 Reporting of clinical and sampling characteristics

Reporting of clinically relevant characteristics was uneven across the 51 independent MRONJ studies. Underlying treatment indication, medication class, and medication route were each reported in 42 studies (82.4%), and lesion location in 43 (84.3%). By contrast, a diagnostic definition was explicitly reported in 24 studies (47.1%), the duration of exposed or probeable bone in 12 (23.5%), exclusion of previous jaw radiotherapy in six (11.8%), and exclusion of metastatic disease involving the jaw in one (2.0%). MRONJ stage was reported in 26 studies (51.0%) and treatment duration in 16 (31.4%). Unclear reporting status was confined to these diagnostic eligibility variables, affecting four studies for diagnostic definition and two studies for each of the remaining three.

Potential clinical modifiers were also incompletely reported. Recent antibiotic exposure was described in 28 studies (54.9%), but its timing relative to specimen collection in only 21 (41.2%). Oral hygiene status and periodontal disease were reported in 12 (23.5%) and 21 studies (41.2%), respectively. Previous dental extraction or surgery was reported in 41 studies (80.4%) and previous debridement in 17 (33.3%). Smoking status was reported in 15 studies (29.4%) and immunosuppression in 23 (45.1%).

Sampling site was reported in 43 studies (84.3%), whereas specimen timing was reported in 23 (45.1%). Although the anatomical source of the specimen was therefore commonly identifiable, its temporal relationship to antibiotic exposure, surgery, debridement, or other clinical events was documented less consistently. Aggregate reporting frequencies are summarized in **Table 3**, and study-level clinical and sampling characteristics are provided in **Supplementary Tables 4A, B**.

**Table 3:** Reporting of key clinical and sampling variables across the MRONJ studies.

Variable	Reported, n/51 (%)	Not reported, n/51 (%)	Unclear, n/51 (%)
Diagnostic definition	24/51 (47.1%)	23/51 (45.1%)	4/51 (7.8%)
Exposed/probeable-bone duration	12/51 (23.5%)	37/51 (72.5%)	2/51 (3.9%)
Exclusion of previous jaw radiotherapy	6/51 (11.8%)	43/51 (84.3%)	2/51 (3.9%)
Exclusion of metastatic disease to the jaw	1/51 (2.0%)	48/51 (94.1%)	2/51 (3.9%)
Underlying indication	42/51 (82.4%)	9/51 (17.6%)	0/51 (0.0%)
Medication class	42/51 (82.4%)	9/51 (17.6%)	0/51 (0.0%)
Medication route	42/51 (82.4%)	9/51 (17.6%)	0/51 (0.0%)
Treatment duration	16/51 (31.4%)	35/51 (68.6%)	0/51 (0.0%)
MRONJ stage	26/51 (51.0%)	25/51 (49.0%)	0/51 (0.0%)
Lesion location	43/51 (84.3%)	8/51 (15.7%)	0/51 (0.0%)
Recent antibiotic exposure	28/51 (54.9%)	23/51 (45.1%)	0/51 (0.0%)
Antibiotic timing	21/51 (41.2%)	30/51 (58.8%)	0/51 (0.0%)
Oral hygiene	12/51 (23.5%)	39/51 (76.5%)	0/51 (0.0%)
Periodontal disease	21/51 (41.2%)	30/51 (58.8%)	0/51 (0.0%)
Prior extraction or dental surgery	41/51 (80.4%)	10/51 (19.6%)	0/51 (0.0%)
Prior debridement	17/51 (33.3%)	34/51 (66.7%)	0/51 (0.0%)
Smoking	15/51 (29.4%)	36/51 (70.6%)	0/51 (0.0%)
Immunosuppression	23/51 (45.1%)	28/51 (54.9%)	0/51 (0.0%)
Specimen timing	23/51 (45.1%)	28/51 (54.9%)	0/51 (0.0%)
Sampling site	43/51 (84.3%)	8/51 (15.7%)	0/51 (0.0%)

### 3.4 Critical appraisal and microbiome reporting assessment

**Figure 3:**
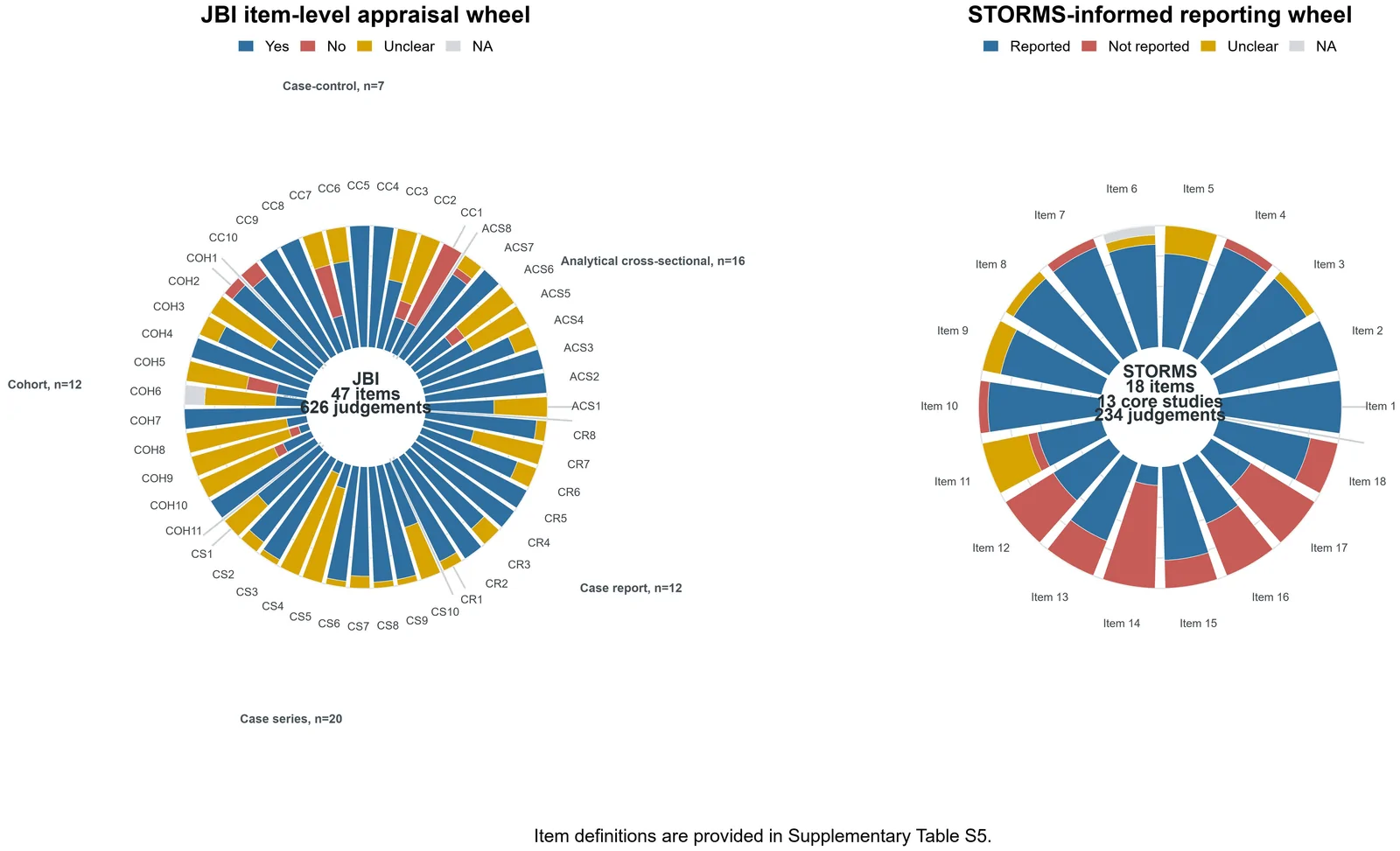
JBI item-level appraisal and STORMS-informed reporting assessment. Panel **(A)** presents design-specific JBI item-level judgments across 67 independent studies: analytical cross-sectional (n = 16), case-control (n = 7), cohort (n = 12), case series (n = 20), and case report (n = 12). Panel **(B)** presents STORMS-informed reporting statuses for 13 core community-level studies. JBI states were classified as yes, no, unclear, or not applicable; STORMS states were classified as reported, not reported, unclear, or not applicable. No total score or overall study grade was calculated. STORMS-informed assessment describes reporting status and is not a risk-of-bias assessment. Item definitions are provided in **Supplementary Table 5**.

Critical appraisal findings are summarized separately by study design and checklist item. No cross-design aggregate percentage, total score, or overall methodological grade was calculated, because the design-specific JBI checklists assess different domains and contain different numbers of items. The complete item-level judgments are presented in **Figure 3** and **Supplementary Table 5**.

The principal limitations differed by design. Among the 16 analytical cross-sectional studies, identification of potential confounders and strategies for addressing confounding were each unclear in eight studies (50.0%), and inclusion criteria were unclear in seven (43.8%). Among the seven case-control studies, comparability of cases and controls was not demonstrated in five (71.4%), matching was unclear in four (57.1%), and strategies for addressing confounding were rated no in three (42.9%). Follow-up reporting was the principal limitation among the 12 cohort studies, with follow-up duration and completeness each unclear in 10 studies (83.3%) and strategies for addressing incomplete follow-up unclear in eight (66.7%). Consecutive and complete inclusion were unclear in 18 (90.0%) and 16 (80.0%), respectively, of the 20 case series. Case reports generally provided the principal clinical and diagnostic information, although adverse or unanticipated events were unclear in seven of 12 (58.3%). Across designs, items concerning clinical and microbiological outcome assessment were more consistently supported than those concerning participant selection, confounding, and follow-up.

The STORMS-informed assessment comprised 234 item-level reporting judgments across the 13 core community-level studies, of which 175 (74.8%) were reported, 45 (19.2%) not reported, 13 (5.6%) unclear, and one (0.4%) not applicable. Sampling site and specimen type were reported in all 13 studies, and specimen collection, specimen storage, sequencing platform, bioinformatics pipeline, and the alpha-diversity method were each reported in 12 (92.3%). By contrast, negative controls were reported in two studies (15.4%) and data availability in five (38.5%). Differential-abundance methods and normalization or rarefaction procedures were each reported in seven studies (53.8%), and beta-diversity methods were reported in seven, unclear in five, and not reported in one.

These items record whether an analytical method was described, and do not indicate that the corresponding analysis was presented. The assessment characterized reporting status rather than risk of bias, and no overall reporting score or grade was assigned.

### 3.5 Community-level microbiome findings

Of the 13 core community-level studies, eight reported at least one alpha-diversity metric and six included an explicit comparison between MRONJ and a comparator group. Chao1 and Shannon indices were each reported in seven studies, Simpson diversity and evenness in four, unspecified richness measures in three, and the abundance-based coverage estimator in two; observed species and phylogenetic distance were each reported in one study. The explicit comparisons involved healthy controls, medication-exposed controls without MRONJ, periodontal controls, treatment-naive controls, and cancer controls. Because the studies used differing combinations of diversity measures and comparator structures, and because the reported directions were neither consistent nor completely extractable, no consistent direction of effect could be established across studies.

Four studies presented beta-diversity analyses, using UniFrac, Bray–Curtis, and Jaccard distance measures, principal coordinates analysis, and permutational multivariate analysis of variance. Twelve of the 13 core studies reported an explicit community-composition or differential result, including comparisons with healthy, medication-exposed, periodontal, or cancer control groups; comparisons between affected and unaffected oral sites; comparisons between antibiotic-treated and untreated patients with bisphosphonate-related osteonecrosis of the jaw; and longitudinal or descriptive community profiles. One study of salivary samples reported a higher average relative abundance of *Actinomyces* in MRONJ than in healthy controls; this comparative abundance result was considered separately from the explicit event-and-denominator observations described in the following section. Other studies reported group-level compositional differences, differential microbial features, anaerobic-genera patterns, microbial-network findings, or temporal changes, but the available numerical information was insufficiently consistent to establish a common direction or magnitude. One study reported community profiles without a separate differential-abundance comparison.

**Figure 4:**
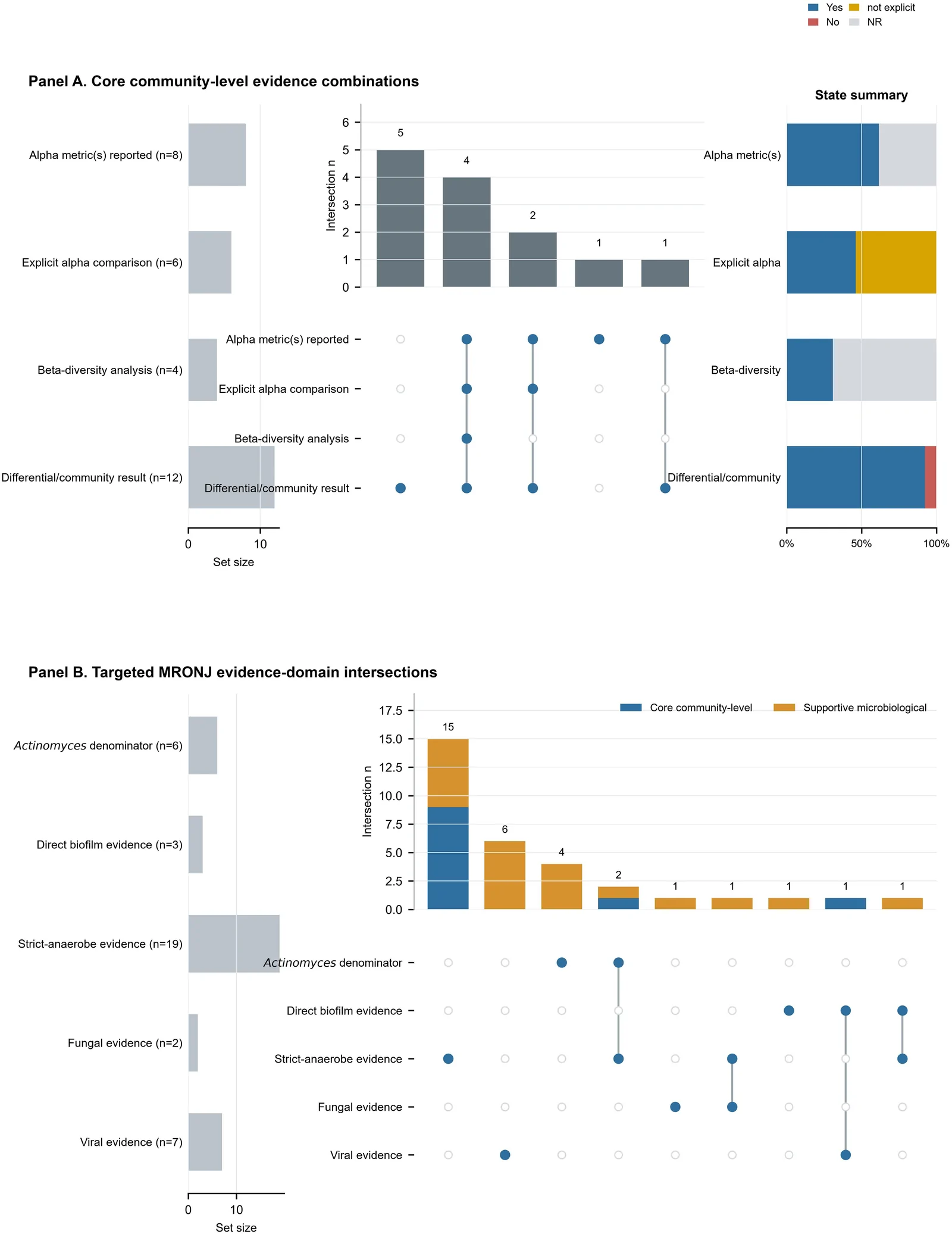
Community-level and targeted microbiological evidence domains in MRONJ. Panel **(A)** summarizes reported alpha-diversity metrics, explicit alpha-diversity comparisons, beta-diversity analyses, and differential or community-composition results among core community-level studies. Panel **(B)** shows overlap among five targeted evidence domains in MRONJ studies: explicit *Actinomyces* event/denominator data, direct biofilm evidence, strict-anaerobe evidence, fungal evidence, and viral evidence. Thirty-two studies contributed at least one targeted evidence domain; 27 contributed one domain and 5 contributed two domains. Viral evidence was identified in 7 studies after the Sedghizadeh correction. Only explicit result-level MRONJ evidence was coded, and contextual studies were excluded from the MRONJ-specific panels. Blank cells indicate that a study did not meet the predefined criterion for that evidence domain and do not necessarily indicate that the domain was not assessed.

The combinations of community-level evidence are summarized in **Figure 4A**. No meta-analysis or pooled quantitative synthesis was performed, because the studies differed in diversity metrics, paired or independent designs, comparator groups, specimen sources, sequencing platforms, amplicon regions, and analytical pipelines. Means, standard deviations, sample sizes, and other compatible numerical summaries were incompletely reported, and no common effect measure was available for beta diversity or differential abundance. Community-level findings were therefore synthesized narratively and through evidence mapping, with study-level data provided in **Supplementary Table 6A** and synthesis decisions summarized in **Table 1**.

### 3.6 Organism- and domain-specific microbiological evidence and feasibility of quantitative synthesis

The organism- and domain-specific evidence map drew on both core community-level and supportive microbiological studies. Among the 51 MRONJ studies, 19 (37.3%) met the prespecified criterion for strict-anaerobe evidence, seven (13.7%) provided viral evidence, six (11.8%) provided explicit MRONJ-specific *Actinomyces* events and denominators, three (5.9%) provided direct biofilm evidence, and two (3.9%) provided fungal evidence. In total, 32 of the 51 MRONJ studies (62.7%) contributed to at least one domain.

Strict-anaerobe evidence was identified in 10 core and nine supportive studies. Viral evidence was reported in seven studies, comprising one core and six supportive studies, and direct biofilm evidence in three studies, comprising one core and two supportive studies; fungal evidence was confined to two supportive studies. Fifteen studies contributed strict-anaerobe evidence alone, six viral evidence alone, four an explicit *Actinomyces* event and denominator alone, one direct biofilm evidence alone, and one fungal evidence alone. Five studies contributed to two domains: two combined *Actinomyces* event and denominator data with strict-anaerobe evidence, one combined direct biofilm with strict-anaerobe evidence, one combined fungal with strict-anaerobe evidence, and one combined direct biofilm with viral evidence. No study contributed to three or more domains. These counts represent fulfillment of prespecified evidence-map criteria and do not represent patient-level frequencies, infection rates, or causal associations (**Figure 4B**; **Supplementary Table 6B**).

**Figure 5:**
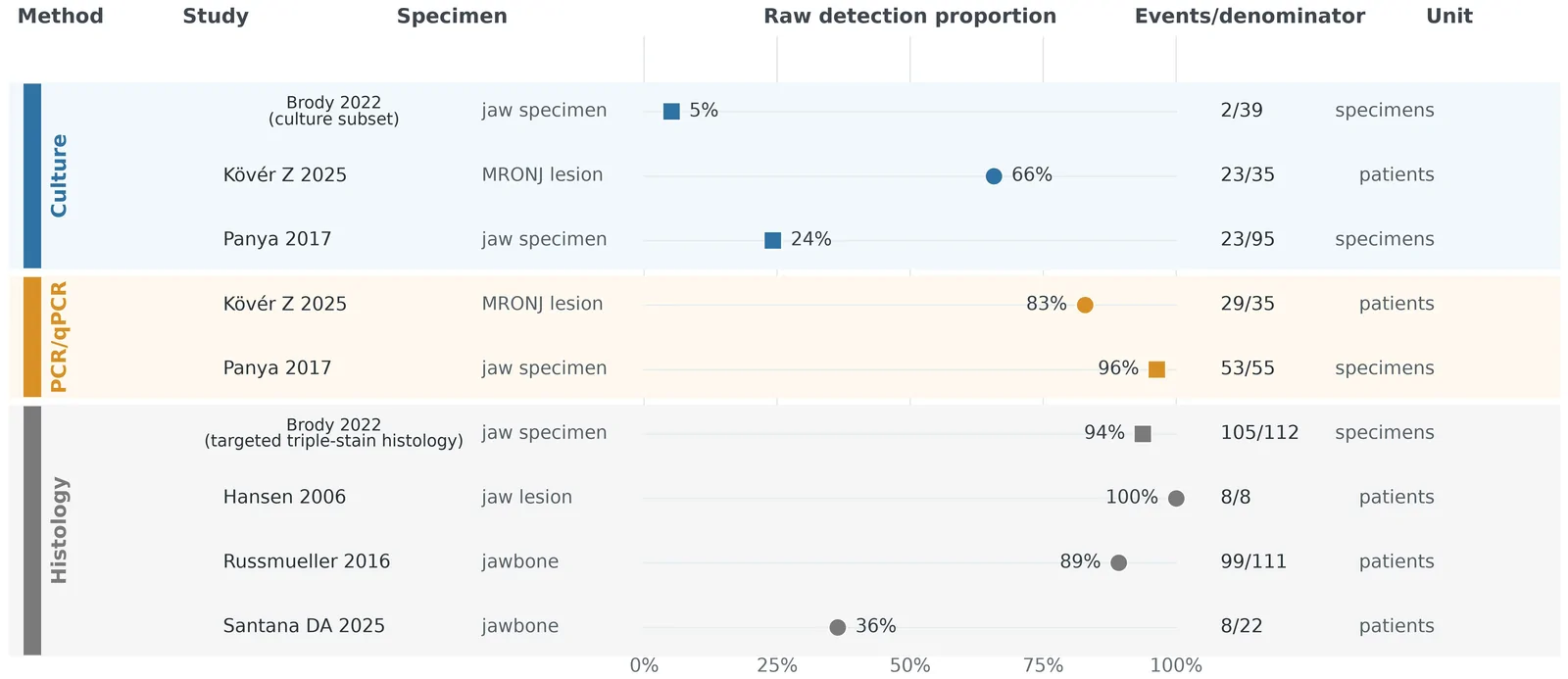
Descriptive study-specific *Actinomyces* detection results in MRONJ by detection method and denominator unit. Each point represents a study–method-specific raw event/denominator result. Raw proportions are displayed only to describe individual result rows and should not be compared across detection methods or denominator units. Panels show culture-based detection, PCR/qPCR-based detection, and histology-based detection. Patient-level and specimen-level denominators are indicated by distinct symbols. Brody et al. (2022) contributed two method-specific observations: microbiological culture results were available for a subset of 39 specimens, whereas targeted triple-stain histological evaluation was performed in 112 specimens. No confidence intervals, study weights, pooled diamond, or pooled estimate are shown, and no cross-method aggregation was performed. Selected surgical or archival specimens and targeted histological assessments are identified in the method labels or notes where applicable. MRONJ, medication-related osteonecrosis of the jaw; PCR, polymerase chain reaction; qPCR, quantitative polymerase chain reaction.

Nine method-specific *Actinomyces* observations from six independent studies provided explicit MRONJ-specific events and denominators. Three observations were culture-based, two used PCR or quantitative PCR, and four used histological detection. Raw proportions ranged from 5.1% to 65.7% for culture, from 82.9% to 96.4% for PCR or quantitative PCR, and from 36.4% to 100.0% for histology. Three studies contributed observations from more than one detection method. Five observations used patients and four used specimens as the denominator, and these units were not treated as interchangeable. These unpooled, denominator-specific proportions should not be interpreted as prevalence estimates. The study- and method-specific observations are displayed in **Figure 5**.

No pooled estimate was calculated for *Actinomyces* detection. The observations differed in specimen type, detection method, routine versus targeted testing, denominator unit, and the basis on which specimens or participants were selected for analysis. Culture, PCR or quantitative PCR, and histology were therefore treated as separate method strata, and patient- and specimen-level observations were not combined. The direct biofilm, strict-anaerobe, fungal, and viral findings were likewise retained as study-level evidence domains, because their detection methods, microbial targets, specimen types, and reporting formats were not sufficiently compatible for meta-analysis.

The 16 contextual studies were synthesized separately from the MRONJ-specific evidence; eight addressed osteoradionecrosis and eight jaw osteomyelitis. Eight used mixed microbiological methods, five reported culture-based findings, two used histological or microscopic detection, and one applied community-level profiling. These studies were retained to characterize microbiological evidence in related jawbone conditions, and did not contribute to MRONJ-specific clinical-reporting denominators, the STORMS-informed assessment, organism-specific domain counts, or MRONJ-specific conclusions. Contextual findings are detailed in **Supplementary Table 6C**, and the overall evidence domains and synthesis approaches are summarized in **Table 1**.

## 4 Discussion

This systematic review synthesized 71 reports representing 67 independent studies, including 51 studies of MRONJ and 16 contextual studies of osteoradionecrosis or jaw osteomyelitis. The MRONJ evidence was separated into 13 core community-level studies and 38 supportive microbiological studies. Across the core studies, microbial diversity and community-composition findings varied according to specimen source, comparator group, sequencing or profiling method, and analytical pipeline. No diversity pattern, taxonomic profile, or community-level feature was reported consistently enough across studies to support a uniform MRONJ microbiome or a clinically validated microbial signature.

Organism- and domain-specific microbial findings were repeatedly reported in established MRONJ lesions, but their reported occurrence varied according to specimen type, detection method, testing strategy, and denominator unit. The prespecified criteria were met by 19 studies for strict-anaerobe evidence, seven for viral evidence, six for explicit MRONJ-specific *Actinomyces* events and denominators, three for direct biofilm evidence, and two for fungal evidence. These study-level counts indicate fulfillment of operational evidence criteria and should not be interpreted as patient-level frequencies or prevalence estimates. *Actinomyces* detection varied markedly across culture, PCR or quantitative PCR, and histological assessment, as well as between patient- and specimen-level denominators. The available observations therefore cannot support a common prevalence estimate or a comparison of diagnostic performance across detection methods.

The principal barrier to meta-analysis was the absence of a sufficiently common estimand rather than merely anticipated statistical heterogeneity. Studies differed in disease definition, medication exposure, specimen type, sampling site and timing, comparator structure, microbiological method, testing strategy, denominator unit, and study design. The contextual studies showed that microbiological investigation of necrotic or chronically inflamed jawbone is not confined to MRONJ, but their indirect and heterogeneous nature did not permit determination of whether any reported microbial feature was specific to MRONJ. Taken together, these observations describe lesion-associated microbial findings whose specificity to MRONJ remains undetermined. The evidence does not establish a causal microbial pathway, a disease-defining organism, a reproducible diagnostic or prognostic signature, or a validated microbial target for therapy.

Community-level profiling describes the composition or diversity of a microbial community, whereas culture, targeted PCR or quantitative PCR, histology, microscopy, and related methods address selected organisms, structures, or molecular signals (McLean, 2014; Pushalkar et al., 2014; Hallmer et al., 2017; Panya et al., 2017; Knight et al., 2018; Brody et al., 2022). These evidence types should therefore be synthesized as distinct evidence streams and should not be combined into a common quantitative estimate. A finding reported in a lesion-only study documents detection or description within the sampled lesion; it does not demonstrate enrichment relative to an unaffected or disease comparator. Conversely, an observed between-group difference in community composition or diversity does not by itself establish diagnostic utility, prognosis, or causal importance.

Eight studies reported alpha-diversity results, but only six included an explicit MRONJ-versus-comparator analysis. These comparisons differed in diversity metrics, comparator definitions, sampling units, and study design, and no compatible set of complete summary statistics was available for quantitative synthesis. The separation of richness from diversity or evenness, of paired from independent comparisons, and of MRONJ-versus-comparator from within-MRONJ analyses is therefore essential to interpretation. The same principle applies to beta-diversity and differential community-composition findings: each result must be interpreted within its own method, comparator, and denominator context.

Histology-based *Actinomyces* observations illustrate the instability of small evidence sets. Three observations reported detection proportions of 89.2%–100.0% (Hansen et al., 2006; Russmueller et al., 2016; Brody et al., 2022), whereas another reported detection in 8 of 22 patients (36.4%) (Santana et al., 2025). With only four observations, and with differences in case selection, specimen processing, staining procedures, denominator units, and targeted versus incidental assessment, any pooled proportion or accompanying heterogeneity statistic would be highly sensitive to the composition of the evidence set. Statistical homogeneity in a small evidence set should not be interpreted as evidence of a stable underlying prevalence.

The lesion-ecology framework is biologically plausible and provides a useful way to organize these findings, but it should not be read as a causal model (Katsarelis et al., 2015; Yarom et al., 2019; Ruggiero et al., 2022). Most included studies examined established lesions using cross-sectional or case-based sampling. The evidence therefore cannot determine whether microbial changes preceded MRONJ onset, contributed to lesion progression, reflected secondary colonization of necrotic tissue, represented chronic infection, or were modified by antibiotic exposure, extraction, debridement, or other treatment. Neither can it establish whether the reported organisms are initiators, persistent residents, opportunistic colonizers, or markers of the local tissue environment.

The appropriate interpretation is consequently narrower: the included studies document microbial features associated with sampled lesions under specific clinical and laboratory conditions. Longitudinal sampling before lesion development, during progression, and after treatment would be required to address temporality. Prospective comparator designs would also be needed to distinguish disease-associated changes from changes related to medication exposure, coexisting oral disease, surgery, or sampling itself.

Microbial findings in MRONJ may be influenced by the underlying indication for antiresorptive or antiangiogenic treatment, medication class and route, treatment duration, disease stage, lesion location, recent antibiotic exposure, oral hygiene, periodontal disease, previous extraction or surgery, prior debridement, smoking, and immunosuppression (Yarom et al., 2019; Ruggiero et al., 2022; Köver et al., 2025). These are not interchangeable clinical descriptors: they may alter host immunity, tissue turnover, lesion ecology, microbial recovery, or the probability of receiving a particular intervention before sampling.

Reporting was insufficient for several of the variables most likely to affect microbial findings. An explicit diagnostic definition was provided in 24 of 51 MRONJ studies, treatment duration in 16, disease stage in 26, and the timing of antibiotic exposure relative to sampling in 21. Previous jaw radiotherapy was explicitly excluded in only six studies. Other potential modifiers, including periodontal disease, previous debridement, smoking, and immunosuppression, were also inconsistently documented. Non-reporting does not establish that these factors were absent or unconsidered, but it prevents reliable assessment of clinical comparability and leaves the target population underlying any pooled estimate poorly defined.

Of the 51 MRONJ studies, 32 (62.7%) met at least one of the five prespecified microbiological-domain criteria. The remaining studies either contributed microbiological evidence outside these five domains or did not report a result meeting any of the five domain criteria. Failure to meet a criterion should therefore not be interpreted as evidence that the corresponding feature was absent.

*Actinomyces* was a recurring lesion-associated finding, but this should not be read as a prevalence estimate or as proof of causation. Nine observations from six independent studies were retained because MRONJ-specific events and corresponding denominators were explicitly reported (Hansen et al., 2006; Russmueller et al., 2016; Panya et al., 2017; Brody et al., 2022; Köver et al., 2025; Santana et al., 2025). Raw detection proportions ranged from 5.1% to 65.7% by culture, from 82.9% to 96.4% by PCR or quantitative PCR, and from 36.4% to 100% by histology. The broad variation across methods is compatible with differences in analytical detectability. Culture requires viable organisms and is affected by transport conditions, anaerobic handling, previous antimicrobial exposure, and the slow growth of *Actinomyces* species (Könönen and Wade, 2015; Panya et al., 2017; Köver et al., 2025). Conventional DNA-based PCR and quantitative PCR detect target nucleic acids without necessarily establishing organism viability and may therefore yield higher detection than culture (Cangelosi and Meschke, 2014), whereas histological identification depends on the presence of recognizable colonies, staining procedures, and whether assessment was targeted or incidental (Hansen et al., 2006; Russmueller et al., 2016; Brody et al., 2022; Santana et al., 2025). Differences in patient selection, specimen type, disease characteristics, prior antimicrobial exposure, and targeted versus incidental testing cannot, however, be excluded. Three studies contributed observations from more than one detection method and reported different method-specific proportions, although these were not formal paired comparisons and may not have involved identical specimens (Russmueller et al., 2016; Panya et al., 2017; Köver et al., 2025). Denominator unit compounds the difficulty: five observations used patients and four used specimens, and a specimen-level proportion and a patient-level proportion are not interchangeable quantities. Reported detection proportions should not be treated as cross-study prevalence estimates. Their interpretation is more meaningful when detection method, specimen type, testing strategy, and denominator unit are comparable, but relative diagnostic performance would require paired head-to-head evaluation against an appropriate reference standard.

Comparable restraint is required for anaerobic and biofilm findings. Anaerobic culture, author-reported classification, and molecular identification of named obligate anaerobes are not equivalent observations, and detection of facultative anaerobes, the presence of *Actinomyces* alone (Könönen and Wade, 2015), descriptions of necrosis or low oxygen tension, general references to polymicrobial infection, and discussion-level mention of biofilm were not accepted as strict-anaerobe evidence. Direct biofilm evidence, requiring visualization or a method explicitly used to demonstrate biofilm architecture, was identified in only three studies. Recovery of multiple taxa from necrotic bone, or observation of dense bacterial masses on routine histology, is compatible with biofilm but does not establish it. Direct evidence requires demonstration of organized microbial communities with spatial architecture and an associated extracellular matrix, often in relation to a tissue or material surface (Donlan and Costerton, 2002; Hall-Stoodley et al., 2004).

Fungal and viral findings were sparse and method-dependent. They were retained as explicit supportive observations where the source report documented a qualifying result in MRONJ material, but they should not be interpreted as evidence that these organisms are universal components of MRONJ lesions. Across all domains, the distinction between detection, comparative enrichment, and an unassessed or unreported domain is essential. The evidence supports the recurrence of selected findings under particular conditions, not a ranked list of microbial causes.

Sixteen contextual studies of osteoradionecrosis and jaw osteomyelitis were retained to delimit the boundary of MRONJ-specific inference. These conditions differ from MRONJ in etiology and diagnostic criteria (Hansen et al., 2006; Goda et al., 2014; Yarom et al., 2019; Ruggiero et al., 2022), and the contextual studies were themselves heterogeneous in specimen type and detection method and were not designed for comparison with MRONJ. They indicate that lesion-associated microbiological investigation of necrotic or chronically inflamed jawbone is not confined to MRONJ, but they cannot establish whether the microbial findings reported in MRONJ are disease-specific or shared with other jawbone conditions. No inference about disease specificity should be drawn from their inclusion, in either direction.

The search was deliberately broad across detection modalities, covering community-level, culture-based, molecular, histological, microscopic, biofilm-directed, fungal, and viral evidence, and was conducted across six sources in two waves, supplemented by backward and forward citation searching. Core community-level evidence was systematically separated from supportive microbiological evidence, avoiding the asymmetry that would arise if community-wide profiling and organism- or feature-directed studies were counted together, because these designs have inherently different probabilities of detecting and reporting a specified microbial feature. Explicit operational criteria were applied to detection, comparative enrichment, direct biofilm evidence, and strict-anaerobe evidence; these criteria prevented background statements, assay target lists, and indirect or discussion-level claims from being counted as direct microbiological evidence.

Critical appraisal was conducted at item level with design-specific instruments and without summary scoring (Barker et al., 2023), in keeping with the structure of those instruments, and because the checklists assess different domains and contain different numbers of items, no cross-design summary was derived. Independent studies rather than reports were used as the unit of analysis, with companion reports clustered and patients, specimens, lesions, and isolates counted only once within any synthesis domain. Meta-analysis was not undertaken where the prespecified compatibility criteria were not met, rather than producing a pooled estimate of uncertain meaning.

Several limitations qualify these findings. The included studies used heterogeneous specimens and sampling procedures, and patient, specimen, lesion, and isolate denominators could not be treated as interchangeable. Many reports lacked the clinical and diagnostic information needed to assess comparability, and incomplete reporting did not permit reliable stratification by indication, medication exposure, disease stage, antibiotic exposure, periodontal disease, or previous treatment. The available core community-level and supportive microbiological results were not sufficiently compatible for meta-analysis without imposing an artificial common estimand, and quantitative pooling was therefore not undertaken for alpha diversity, *Actinomyces*, or any other microbial domain. A formal certainty-of-evidence framework was not applied because the synthesis did not yield a sufficiently standardized outcome or effect estimate to which a common certainty judgment could be meaningfully assigned; confidence in these findings rests instead on the item-level appraisal, the reporting assessment, and the transparency of the mapping criteria.

Many supportive studies were small and organism-directed, creating potential for selective testing and preferential publication of positive detections. Specimens may also have been selected for additional testing because of clinical, histological, or microbiological suspicion. Consequently, the number of studies meeting a domain criterion should not be interpreted as the frequency of that feature among all patients, lesions, or specimens with MRONJ.

Appraisal limitations were concentrated in participant selection, confounding, and follow-up: strategies for addressing confounding were unclear in half of the analytical cross-sectional studies, follow-up duration and completeness were unclear in 10 of 12 cohort studies, and consecutive inclusion was unclear in 18 of 20 case series. Items concerning clinical and microbiological outcome assessment were more frequently supported, suggesting that participant selection, clinical characterization, confounding, and follow-up were major sources of methodological concern. This should not, however, be interpreted as evidence that microbiological measurement and reporting were free of limitation. The STORMS-informed items described reporting status in core studies and did not constitute a risk-of-bias assessment; a reporting gap should not be converted into a judgment that a study failed to perform the relevant procedure (Mirzayi et al., 2021).

Two reporting gaps among the core studies are nonetheless consequential. Negative controls were reported in only 2 of 13 studies, and data availability was reported in five. Contamination control is particularly important for bone and tissue microbiome studies, in which microbial biomass may be low or highly variable and in which conclusions concerning low-abundance taxa cannot be adequately evaluated without appropriate controls (Salter et al., 2014; Knight et al., 2018; Eisenhofer et al., 2019; Mirzayi et al., 2021). The limited availability of deposited sequence data further restricts independent reanalysis and sensitivity analyses using harmonized bioinformatic pipelines (Wilkinson et al., 2016; Knight et al., 2018; Mirzayi et al., 2021). Such reanalysis could help quantify the contribution of analytical choices, although differences in amplicon region, sequencing platform, specimen processing, and available metadata may not be fully resolvable.

Finally, 17 reports remained unavailable after repeated retrieval attempts and their contents are unknown; unavailable reports were kept distinct from full-text exclusions. ClinicalTrials.gov was searched but contributed no eligible study or report. The search strategies were internally verified and tested for known-item recall but were not formally peer-reviewed by an external information specialist. Evidence classification, although based on explicit operational criteria, necessarily involved judgment where source reporting was ambiguous, and items so affected were coded conservatively as unclear.

For clinical practice, the implications are conservative. Although microbiological findings were frequently reported in studies of established MRONJ lesions, the current evidence does not support their use for diagnosis, staging, or prognosis, and no organism reported in the included literature is established as a disease-defining marker or validated therapeutic target. Where microbiological testing is performed for clinical reasons, its interpretation should take explicit account of specimen type, detection method, recent antimicrobial exposure, and the timing of sampling relative to surgery or debridement, and detection should not be equated with infection (Yarom et al., 2019; Ruggiero et al., 2022).

For research, additional small descriptive series using unstandardized lesion-only sampling and lacking clinically informative comparators are unlikely to substantially advance the field. Future studies should apply an explicit and reproducible MRONJ definition and report whether diagnostic criteria, duration requirements, radiotherapy exclusions, and metastatic-disease exclusions were applied (Yarom et al., 2019; Ruggiero et al., 2022). Clinical metadata should include underlying indication, medication class and route, treatment duration, disease stage, lesion location, recent antibiotic exposure and its timing, periodontal status, oral hygiene, previous extraction or surgery, debridement, smoking, and immunosuppression.

Sampling should be prospectively standardized. Studies should state whether the unit of analysis is a patient, specimen, lesion, or isolate; distinguish lesion-associated bone or tissue from saliva and other oral samples; and report sampling timing relative to treatment, antibiotic exposure, extraction, or debridement. Community-level studies should report the profiling method and, where applicable, the amplicon region and sequencing platform, together with quality-control procedures, negative controls, contamination-control procedures, and reproducible analysis metadata (Salter et al., 2014; Knight et al., 2018; Eisenhofer et al., 2019; Mirzayi et al., 2021), and should deposit raw sequence data (Wilkinson et al., 2016). Organism- or feature-directed studies should identify the assay, the rule by which specimens were selected, and whether the denominator represents all eligible samples or a selected subset.

Comparative and longitudinal designs are the highest priority. Well-matched medication-exposed patients without MRONJ are generally the most informative comparator for separating MRONJ-associated differences from those related to medication exposure, with healthy participants as a secondary reference; matching should ideally address underlying indication, medication class, route and duration, age, periodontal and other oral disease, recent antibiotic exposure, and relevant dental procedures. Paired sampling of affected and clinically unaffected sites within the same patient may reduce between-person variation in the oral microbiome background and is feasible in single-center settings without requiring multicenter infrastructure. Prospective repeated sampling before and after high-risk events such as extraction would be among the most informative designs for addressing temporal order. Results should distinguish detection from enrichment and should report extractable effect measures or complete descriptive data without combining incompatible methods or denominator units. Reporting complete group-level numerical summaries appropriate to the observed distribution, including means and standard deviations or medians and interquartile ranges as applicable, together with group sizes and between-group effect estimates with confidence intervals, would materially improve the interpretability and future synthesis of diversity findings.

## 5 Conclusion

The current evidence documents recurrent microbial findings of undetermined specificity in established MRONJ lesions. It does not establish a single reproducible microbial signature, a reliable prevalence estimate for any organism, the diagnostic accuracy of any microbial feature, or a causal role for the organisms detected. Reported detection proportions varied substantially according to detection method, specimen type, testing strategy, and denominator unit, limiting their interpretation as estimates of organism prevalence in MRONJ. Strict-anaerobe evidence was heterogeneous and method-dependent, whereas direct evidence of biofilm architecture was sparse. *Actinomyces*, anaerobic and biofilm-related findings, and the sparse fungal and viral observations should therefore be interpreted as method- and specimen-specific evidence within a broader lesion-associated ecological framework. More informative conclusions will require prospective, longitudinal, clinically stratified studies with standardized sampling, explicit denominators, transparent diagnostic reporting, contamination controls, and reproducible microbiological methods with publicly accessible data.

## Data Availability

The minimal review-level dataset supporting the findings of this study is publicly available in Zenodo (https://doi.org/10.5281/zenodo.22151668). The dataset contains the de-identified study-level extraction, evidence-domain coding, clinical reporting-status data, Actinomyces descriptive event/denominator data, and JBI/STORMS assessment data used in this systematic review and evidence map. This study did not generate new biological specimens or raw sequencing data.
