# Supplementary material for: Oral microbiome and lesion-associated microbiological findings in medication-related osteonecrosis of the jaw: a systematic review and evidence map
Source: Front Cell Infect Microbiol. 2026 Sep 8;16:1923000. doi: 10.3389/fcimb.2026.1923000 (PMC13593453; doi:10.3389/fcimb.2026.1923000)
Supplement: Supplementary file 1 [file DataSheet1.pdf]

Supplementary Material S1. Complete electronic search strategies

S1.1 Search overview

The review used two search waves across six sources: PubMed/MEDLINE, Embase.com, Scopus, Web of Science Core Collection, the Cochrane Library, and ClinicalTrials.gov. The initial wave was conducted on 25 June 2026 and the definitive updated wave on 25 July 2026. Each wave used a source-specific combination of MRONJ and historical related terms, contextual jaw-disease terms, and microbiological concepts. The updated wave broadened the microbiological concepts to include community profiling, targeted detection, culture, molecular methods, histology, biofilm, fungal, and viral terminology. No language, publication-date, study-design, or publication-type restrictions were applied; ClinicalTrials.gov was searched without date, language, or study-type restrictions. The strategies were developed by the review team and were not formally peer-reviewed by a professional information specialist or medical librarian.

S1.2 Initial search wave (25 June 2026)

PubMed/MEDLINE

Platform: PubMed (National Library of Medicine)

Date searched: 25 June 2026

Records retrieved: 306

Search field/interface: Title/Abstract search

Exact search strategy

(("medication-related osteonecrosis of the jaw"[Title/Abstract] OR MRONJ[Title/Abstract] OR "bisphosphonate-related osteonecrosis of the jaw"[Title/Abstract] OR BRONJ[Title/Abstract] OR "antiresorptive-related osteonecrosis of the jaw"[Title/Abstract] OR ARONJ[Title/Abstract] OR "drug-related osteonecrosis of the jaw"[Title/Abstract] OR DRONJ[Title/Abstract] OR "osteonecrosis of the jaw"[Title/Abstract] OR "jaw osteonecrosis"[Title/Abstract] OR osteoradionecrosis[Title/Abstract] OR "osteomyelitis of the jaw"[Title/Abstract] OR "jaw osteomyelitis"[Title/Abstract]) AND (microbiome[Title/Abstract] OR microbiota[Title/Abstract] OR microbial[Title/Abstract] OR bacteria[Title/Abstract] OR bacterial[Title/Abstract] OR biofilm[Title/Abstract] OR Actinomyces[Title/Abstract] OR "16S"[Title/Abstract] OR metagenomic[Title/Abstract] OR metagenomics[Title/Abstract] OR sequencing[Title/Abstract] OR "next-generation sequencing"[Title/Abstract] OR NGS[Title/Abstract]))

Embase

Platform: Embase.com (Elsevier)

Date searched: 25 June 2026

Records retrieved: 404

Search field/interface: ti,ab,kw search

Exact search strategy

(('medication-related osteonecrosis of the jaw':ti,ab,kw OR MRONJ:ti,ab,kw OR 'bisphosphonate-related osteonecrosis of the jaw':ti,ab,kw OR BRONJ:ti,ab,kw OR 'antiresorptive-related osteonecrosis of the jaw':ti,ab,kw OR ARONJ:ti,ab,kw OR 'drug-related osteonecrosis of the jaw':ti,ab,kw OR DRONJ:ti,ab,kw OR 'osteonecrosis of the jaw':ti,ab,kw OR 'jaw osteonecrosis':ti,ab,kw OR osteoradionecrosis:ti,ab,kw OR 'osteomyelitis of the jaw':ti,ab,kw OR 'jaw osteomyelitis':ti,ab,kw) AND (microbiome:ti,ab,kw OR microbiota:ti,ab,kw OR microbial:ti,ab,kw OR bacteria:ti,ab,kw OR bacterial:ti,ab,kw OR biofilm:ti,ab,kw OR actinomyces:ti,ab,kw OR '16S':ti,ab,kw OR metagenomic:ti,ab,kw OR metagenomics:ti,ab,kw OR sequencing:ti,ab,kw OR 'next-generation sequencing':ti,ab,kw OR NGS:ti,ab,kw))

Scopus

Platform: Scopus (Elsevier)

Date searched: 25 June 2026

Records retrieved: 817

Search field/interface: TITLE-ABS-KEY

Exact search strategy

TITLE-ABS-KEY(("medication-related osteonecrosis of the jaw" OR MRONJ OR "bisphosphonate-related osteonecrosis of the jaw" OR BRONJ OR "antiresorptive-related osteonecrosis of the jaw" OR ARONJ OR "drug-related osteonecrosis of the jaw" OR DRONJ OR "osteonecrosis of the jaw" OR "jaw osteonecrosis" OR osteoradionecrosis OR "osteomyelitis of the jaw" OR "jaw osteomyelitis") AND (microbiome OR microbiota OR microbial OR bacteria OR bacterial OR biofilm OR Actinomyces OR "16S" OR metagenomic OR metagenomics OR sequencing OR "next-generation sequencing" OR NGS))

Web of Science Core Collection

Platform: Web of Science Core Collection (Clarivate)

Date searched: 25 June 2026

Records retrieved: 350

Search field/interface: Topic search (TS)

Exact search strategy

TS=(("medication-related osteonecrosis of the jaw" OR MRONJ OR "bisphosphonate-related osteonecrosis of the jaw" OR BRONJ OR "antiresorptive-related osteonecrosis of the jaw" OR ARONJ OR "drug-related osteonecrosis of the jaw" OR DRONJ OR "osteonecrosis of the jaw" OR "jaw osteonecrosis" OR osteoradionecrosis OR "osteomyelitis of the jaw" OR "jaw osteomyelitis") AND (microbiome OR microbiota OR microbial OR bacteria OR bacterial OR biofilm OR Actinomyces OR "16S" OR metagenomic OR metagenomics OR sequencing OR "next-generation sequencing" OR NGS))

Cochrane Library

Platform: Cochrane Library (Wiley Search Manager)

Date searched: 25 June 2026

Records retrieved: 17

Search field/interface: Search Manager / all-text interface

Exact search strategy

(("medication-related osteonecrosis of the jaw" OR MRONJ OR "bisphosphonate-related osteonecrosis of the jaw" OR BRONJ OR "antiresorptive-related osteonecrosis of the jaw" OR ARONJ OR "drug-related osteonecrosis of the jaw" OR DRONJ OR "osteonecrosis of the jaw" OR "jaw osteonecrosis" OR osteoradionecrosis OR "osteomyelitis of the jaw" OR "jaw osteomyelitis") AND (microbiome OR microbiota OR microbial OR bacteria OR bacterial OR biofilm OR Actinomyces OR "16S" OR metagenomic OR metagenomics OR sequencing OR "next-generation sequencing" OR NGS)):ti,ab,kw

ClinicalTrials.gov

Platform: ClinicalTrials.gov (NLM registry)

Date searched: 25 June 2026

Records retrieved: 1

Search field/interface: Expert Search or standard search box

Exact search strategy

("medication-related osteonecrosis of the jaw" OR MRONJ OR "bisphosphonate-related osteonecrosis of the jaw" OR BRONJ OR "antiresorptive-related osteonecrosis of the jaw" OR ARONJ OR "drug-related osteonecrosis of the jaw" OR DRONJ OR "osteonecrosis of the jaw" OR "jaw osteonecrosis" OR osteoradionecrosis OR "osteomyelitis of the jaw" OR "jaw osteomyelitis") AND (microbiome OR microbiota OR microbial OR bacteria OR bacterial OR biofilm OR Actinomyces OR "16S" OR metagenomic OR metagenomics OR sequencing OR "next-generation sequencing" OR NGS)

S1.3 Updated search wave (25 July 2026; definitive)

PubMed/MEDLINE

Platform: PubMed (National Library of Medicine)
Date searched: 25 July 2026
Records retrieved: 383
Search field/interface: MeSH plus Title/Abstract fields

Exact search strategy

(("Bisphosphonate-Associated Osteonecrosis of the Jaw"[Mesh] OR "medication-related osteonecrosis of the jaw"[Title/Abstract] OR "medication related osteonecrosis of the jaw"[Title/Abstract] OR MRONJ[Title/Abstract] OR "bisphosphonate-related osteonecrosis of the jaw"[Title/Abstract] OR "bisphosphonate related osteonecrosis of the jaw"[Title/Abstract] OR BRONJ[Title/Abstract] OR "antiresorptive-related osteonecrosis of the jaw"[Title/Abstract] OR "antiresorptive related osteonecrosis of the jaw"[Title/Abstract] OR ARONJ[Title/Abstract] OR "drug-related osteonecrosis of the jaw"[Title/Abstract] OR "drug related osteonecrosis of the jaw"[Title/Abstract] OR DRONJ[Title/Abstract] OR (("osteonecrosis of the jaw"[Title/Abstract] OR "osteonecrosis of jaws"[Title/Abstract] OR "jaw osteonecrosis"[Title/Abstract]) AND (medication[Title/Abstract] OR medications[Title/Abstract] OR drug[Title/Abstract] OR drugs[Title/Abstract] OR bisphosphonate[Title/Abstract] OR bisphosphonates[Title/Abstract] OR denosumab[Title/Abstract] OR antiresorptive[Title/Abstract] OR antiangiogenic[Title/Abstract]))) AND ("Microbiota"[Mesh] OR "Microbiological Techniques"[Mesh] OR "Polymerase Chain Reaction"[Mesh] OR "Real-Time Polymerase Chain Reaction"[Mesh] OR "Biofilms"[Mesh] OR "Actinomyces"[Mesh] OR "Bacteria, Anaerobic"[Mesh] OR "Fungi"[Mesh] OR "Candida"[Mesh] OR "Viruses"[Mesh] OR "Herpesvirus 4, Human"[Mesh] OR "Epstein-Barr Virus Infections"[Mesh] OR microbiome[Title/Abstract] OR microbiota[Title/Abstract] OR microflora[Title/Abstract] OR "oral flora"[Title/Abstract] OR "microbial composition"[Title/Abstract] OR "microbial profile"[Title/Abstract] OR "microbial profiles"[Title/Abstract] OR "bacterial profile"[Title/Abstract] OR "bacterial profiles"[Title/Abstract] OR microbiological[Title/Abstract] OR microbiology[Title/Abstract] OR dysbiosis[Title/Abstract] OR PCR[Title/Abstract] OR qPCR[Title/Abstract] OR "polymerase chain reaction"[Title/Abstract] OR "quantitative polymerase chain reaction"[Title/Abstract] OR "real-time PCR"[Title/Abstract] OR "real time PCR"[Title/Abstract] OR "molecular detection"[Title/Abstract] OR "molecular profiling"[Title/Abstract] OR 16S[Title/Abstract] OR "16S rRNA"[Title/Abstract] OR metagenomic[Title/Abstract] OR metagenomics[Title/Abstract] OR sequencing[Title/Abstract] OR "next-generation sequencing"[Title/Abstract] OR "next generation sequencing"[Title/Abstract] OR NGS[Title/Abstract] OR DGGE[Title/Abstract] OR "denaturing gradient gel electrophoresis"[Title/Abstract] OR biofilm[Title/Abstract] OR biofilms[Title/Abstract] OR Actinomyces[Title/Abstract] OR actinomycosis[Title/Abstract] OR actinomycotic[Title/Abstract] OR Schaalia[Title/Abstract] OR "Actinomyces-like organism"[Title/Abstract] OR "Actinomyces-like organisms"[Title/Abstract] OR anaerobe[Title/Abstract] OR anaerobes[Title/Abstract] OR "anaerobic bacteria"[Title/Abstract] OR fungus[Title/Abstract] OR fungi[Title/Abstract] OR fungal[Title/Abstract] OR yeast[Title/Abstract] OR yeasts[Title/Abstract] OR Candida[Title/Abstract] OR candidiasis[Title/Abstract] OR mycosis[Title/Abstract] OR mycobiome[Title/Abstract] OR mycobiota[Title/Abstract] OR Aspergillus[Title/Abstract] OR aspergillosis[Title/Abstract] OR mucormycosis[Title/Abstract] OR virus[Title/Abstract] OR viruses[Title/Abstract] OR viral[Title/Abstract] OR virome[Title/Abstract] OR "Epstein-Barr virus"[Title/Abstract] OR EBV[Title/Abstract] OR EBVMCU[Title/Abstract] OR "methotrexate-associated lymphoproliferative disorder"[Title/Abstract] OR "methotrexate-associated lymphoproliferative disorders"[Title/Abstract] OR MTX-LPD[Title/Abstract] OR

((culture[Title/Abstract] OR cultures[Title/Abstract] OR cultured[Title/Abstract] OR isolation[Title/Abstract] OR isolated[Title/Abstract]) AND (microorganism[Title/Abstract] OR microorganisms[Title/Abstract] OR bacterium[Title/Abstract] OR bacteria[Title/Abstract] OR bacterial[Title/Abstract] OR Actinomyces[Title/Abstract] OR anaerobic[Title/Abstract] OR fungus[Title/Abstract] OR fungal[Title/Abstract] OR Candida[Title/Abstract] OR Aspergillus[Title/Abstract])) OR ((histology[Title/Abstract] OR histological[Title/Abstract] OR histopathology[Title/Abstract] OR histopathological[Title/Abstract] OR microscopy[Title/Abstract] OR microscopic[Title/Abstract] OR staining[Title/Abstract] OR "special stain"[Title/Abstract] OR "in situ hybridization"[Title/Abstract]) AND (microorganism[Title/Abstract] OR microorganisms[Title/Abstract] OR bacterium[Title/Abstract] OR bacteria[Title/Abstract] OR bacterial[Title/Abstract] OR Actinomyces[Title/Abstract] OR actinomycotic[Title/Abstract] OR fungus[Title/Abstract] OR fungal[Title/Abstract] OR Candida[Title/Abstract] OR Aspergillus[Title/Abstract] OR virus[Title/Abstract] OR viral[Title/Abstract] OR EBV[Title/Abstract])) OR ((infection[Title/Abstract] OR infections[Title/Abstract] OR infected[Title/Abstract] OR colonization[Title/Abstract] OR colonisation[Title/Abstract]) AND (microbiological[Title/Abstract] OR microbiology[Title/Abstract] OR microorganism[Title/Abstract] OR microorganisms[Title/Abstract] OR Actinomyces[Title/Abstract] OR actinomycosis[Title/Abstract] OR anaerobic[Title/Abstract] OR Candida[Title/Abstract] OR Aspergillus[Title/Abstract] OR "Epstein-Barr virus"[Title/Abstract] OR EBV[Title/Abstract] OR PCR[Title/Abstract] OR culture[Title/Abstract])))) OR (("osteoradionecrosis of the jaw"[Title/Abstract] OR "osteoradionecrosis of the jaws"[Title/Abstract] OR "jaw osteoradionecrosis"[Title/Abstract] OR (osteoradionecrosis[Title/Abstract] AND (jaw[Title/Abstract] OR jaws[Title/Abstract] OR mandibular[Title/Abstract] OR maxillary[Title/Abstract] OR maxillofacial[Title/Abstract])) OR "osteomyelitis of the jaw"[Title/Abstract] OR "osteomyelitis of the jaws"[Title/Abstract] OR "jaw osteomyelitis"[Title/Abstract] OR "chronic osteomyelitis of the jaw"[Title/Abstract] OR "chronic osteomyelitis of the jaws"[Title/Abstract] OR "chronic jaw osteomyelitis"[Title/Abstract]) AND ("Microbiota"[Mesh] OR "Biofilms"[Mesh] OR "Actinomyces"[Mesh] OR "Bacteria, Anaerobic"[Mesh] OR "Fungi"[Mesh] OR "Candida"[Mesh] OR "Viruses"[Mesh] OR microbiome[Title/Abstract] OR microbiota[Title/Abstract] OR microflora[Title/Abstract] OR "microbial composition"[Title/Abstract] OR "microbial profile"[Title/Abstract] OR "microbial profiles"[Title/Abstract] OR "bacterial profile"[Title/Abstract] OR "bacterial profiles"[Title/Abstract] OR microbiological[Title/Abstract] OR microbiology[Title/Abstract] OR PCR[Title/Abstract] OR qPCR[Title/Abstract] OR "polymerase chain reaction"[Title/Abstract] OR "molecular detection"[Title/Abstract] OR 16S[Title/Abstract] OR "16S rRNA"[Title/Abstract] OR metagenomic[Title/Abstract] OR metagenomics[Title/Abstract] OR sequencing[Title/Abstract] OR DGGE[Title/Abstract] OR biofilm[Title/Abstract] OR biofilms[Title/Abstract] OR Actinomyces[Title/Abstract] OR actinomycosis[Title/Abstract] OR actinomycotic[Title/Abstract] OR anaerobe[Title/Abstract] OR anaerobes[Title/Abstract] OR "anaerobic bacteria"[Title/Abstract] OR fungus[Title/Abstract] OR fungi[Title/Abstract] OR fungal[Title/Abstract] OR Candida[Title/Abstract] OR mycobiome[Title/Abstract] OR Aspergillus[Title/Abstract] OR mucormycosis[Title/Abstract] OR virus[Title/Abstract] OR viruses[Title/Abstract] OR viral[Title/Abstract] OR virome[Title/Abstract] OR "Epstein-Barr virus"[Title/Abstract] OR EBV[Title/Abstract] OR ((culture[Title/Abstract] OR cultures[Title/Abstract] OR cultured[Title/Abstract] OR isolation[Title/Abstract] OR isolated[Title/Abstract]) AND (microorganism[Title/Abstract] OR microorganisms[Title/Abstract] OR Actinomyces[Title/Abstract] OR anaerobic[Title/Abstract] OR fungus[Title/Abstract] OR fungal[Title/Abstract] OR Candida[Title/Abstract] OR Aspergillus[Title/Abstract])) OR ((histology[Title/Abstract] OR histological[Title/Abstract] OR histopathology[Title/Abstract] OR histopathological[Title/Abstract] OR microscopy[Title/Abstract] OR staining[Title/Abstract]) AND (microorganism[Title/Abstract] OR microorganisms[Title/Abstract] OR Actinomyces[Title/Abstract] OR actinomycotic[Title/Abstract] OR fungal[Title/Abstract] OR Candida[Title/Abstract] OR viral[Title/Abstract] OR EBV[Title/Abstract])))))

Embase

Platform: Embase.com (Elsevier)
Date searched: 25 July 2026
Records retrieved: 1,035
Search field/interface: Emtree plus ti,ab,kw fields

Exact search strategy

(("Bisphosphonate-Associated Osteonecrosis of the Jaw"[Mesh] OR "medication-related osteonecrosis of the jaw"[Title/Abstract] OR "medication related osteonecrosis of the jaw"[Title/Abstract] OR MRONJ[Title/Abstract] OR "bisphosphonate-related osteonecrosis of the jaw"[Title/Abstract] OR "bisphosphonate related osteonecrosis of the jaw"[Title/Abstract] OR BRONJ[Title/Abstract] OR "antiresorptive-related osteonecrosis of the jaw"[Title/Abstract] OR "antiresorptive related osteonecrosis of the jaw"[Title/Abstract] OR ARONJ[Title/Abstract] OR "drug-related osteonecrosis of the jaw"[Title/Abstract] OR "drug related osteonecrosis of the jaw"[Title/Abstract] OR DRONJ[Title/Abstract] OR (("osteonecrosis of the jaw"[Title/Abstract] OR "osteonecrosis of jaws"[Title/Abstract] OR "jaw osteonecrosis"[Title/Abstract]) AND (medication[Title/Abstract] OR medications[Title/Abstract] OR drug[Title/Abstract] OR drugs[Title/Abstract] OR bisphosphonate[Title/Abstract] OR bisphosphonates[Title/Abstract] OR denosumab[Title/Abstract] OR antiresorptive[Title/Abstract] OR antiangiogenic[Title/Abstract]))) AND ("Microbiota"[Mesh] OR "Microbiological Techniques"[Mesh] OR "Polymerase Chain Reaction"[Mesh] OR "Real-Time Polymerase Chain Reaction"[Mesh] OR "Biofilms"[Mesh] OR "Actinomyces"[Mesh] OR "Bacteria, Anaerobic"[Mesh] OR "Fungi"[Mesh] OR "Candida"[Mesh] OR "Viruses"[Mesh] OR "Herpesvirus 4, Human"[Mesh] OR "Epstein-Barr Virus Infections"[Mesh] OR microbiome[Title/Abstract] OR microbiota[Title/Abstract] OR microflora[Title/Abstract] OR "oral flora"[Title/Abstract] OR "microbial composition"[Title/Abstract] OR "microbial profile"[Title/Abstract] OR "microbial profiles"[Title/Abstract] OR "bacterial profile"[Title/Abstract] OR "bacterial profiles"[Title/Abstract] OR microbiological[Title/Abstract] OR microbiology[Title/Abstract] OR dysbiosis[Title/Abstract] OR PCR[Title/Abstract] OR qPCR[Title/Abstract] OR "polymerase chain reaction"[Title/Abstract] OR "quantitative polymerase chain reaction"[Title/Abstract] OR "real-time PCR"[Title/Abstract] OR "real time PCR"[Title/Abstract] OR "molecular detection"[Title/Abstract] OR "molecular profiling"[Title/Abstract] OR 16S[Title/Abstract] OR "16S rRNA"[Title/Abstract] OR metagenomic[Title/Abstract] OR metagenomics[Title/Abstract] OR sequencing[Title/Abstract] OR "next-generation sequencing"[Title/Abstract] OR "next generation sequencing"[Title/Abstract] OR NGS[Title/Abstract] OR DGGE[Title/Abstract] OR "denaturing gradient gel electrophoresis"[Title/Abstract] OR biofilm[Title/Abstract] OR biofilms[Title/Abstract] OR Actinomyces[Title/Abstract] OR actinomycosis[Title/Abstract] OR actinomycotic[Title/Abstract] OR Schaalia[Title/Abstract] OR "Actinomyces-like organism"[Title/Abstract] OR "Actinomyces-like organisms"[Title/Abstract] OR anaerobe[Title/Abstract] OR anaerobes[Title/Abstract] OR "anaerobic bacteria"[Title/Abstract] OR fungus[Title/Abstract] OR fungi[Title/Abstract] OR fungal[Title/Abstract] OR yeast[Title/Abstract] OR yeasts[Title/Abstract] OR Candida[Title/Abstract] OR candidiasis[Title/Abstract] OR mycosis[Title/Abstract] OR mycobiome[Title/Abstract] OR mycobiota[Title/Abstract] OR Aspergillus[Title/Abstract] OR aspergillosis[Title/Abstract] OR mucormycosis[Title/Abstract] OR virus[Title/Abstract] OR viruses[Title/Abstract] OR viral[Title/Abstract] OR virome[Title/Abstract] OR "Epstein-Barr virus"[Title/Abstract] OR EBV[Title/Abstract] OR EBVMCU[Title/Abstract] OR "methotrexate-associated lymphoproliferative disorder"[Title/Abstract] OR "methotrexate-associated lymphoproliferative disorders"[Title/Abstract] OR MTX-LPD[Title/Abstract] OR ((culture[Title/Abstract] OR cultures[Title/Abstract] OR cultured[Title/Abstract] OR isolation[Title/Abstract] OR isolated[Title/Abstract]) AND (microorganism[Title/Abstract] OR microorganisms[Title/Abstract] OR bacterium[Title/Abstract] OR bacteria[Title/Abstract] OR bacterial[Title/Abstract] OR Actinomyces[Title/Abstract] OR anaerobic[Title/Abstract] OR fungus[Title/Abstract] OR fungal[Title/Abstract] OR Candida[Title/Abstract] OR

Aspergillus[Title/Abstract])) OR ((histology[Title/Abstract] OR histological[Title/Abstract] OR histopathology[Title/Abstract] OR histopathological[Title/Abstract] OR microscopy[Title/Abstract] OR microscopic[Title/Abstract] OR staining[Title/Abstract] OR "special stain"[Title/Abstract] OR "in situ hybridization"[Title/Abstract]) AND (microorganism[Title/Abstract] OR microorganisms[Title/Abstract] OR bacterium[Title/Abstract] OR bacteria[Title/Abstract] OR bacterial[Title/Abstract] OR Actinomyces[Title/Abstract] OR actinomycotic[Title/Abstract] OR fungus[Title/Abstract] OR fungal[Title/Abstract] OR Candida[Title/Abstract] OR Aspergillus[Title/Abstract] OR virus[Title/Abstract] OR viral[Title/Abstract] OR EBV[Title/Abstract])) OR ((infection[Title/Abstract] OR infections[Title/Abstract] OR infected[Title/Abstract] OR colonization[Title/Abstract] OR colonisation[Title/Abstract]) AND (microbiological[Title/Abstract] OR microbiology[Title/Abstract] OR microorganism[Title/Abstract] OR microorganisms[Title/Abstract] OR Actinomyces[Title/Abstract] OR actinomycosis[Title/Abstract] OR anaerobic[Title/Abstract] OR Candida[Title/Abstract] OR Aspergillus[Title/Abstract] OR "Epstein-Barr virus"[Title/Abstract] OR EBV[Title/Abstract] OR PCR[Title/Abstract] OR culture[Title/Abstract])))) OR (("osteoradionecrosis of the jaw"[Title/Abstract] OR "osteoradionecrosis of the jaws"[Title/Abstract] OR "jaw osteoradionecrosis"[Title/Abstract] OR (osteoradionecrosis[Title/Abstract] AND (jaw[Title/Abstract] OR jaws[Title/Abstract] OR mandibular[Title/Abstract] OR maxillary[Title/Abstract] OR maxillofacial[Title/Abstract])) OR "osteomyelitis of the jaw"[Title/Abstract] OR "osteomyelitis of the jaws"[Title/Abstract] OR "jaw osteomyelitis"[Title/Abstract] OR "chronic osteomyelitis of the jaw"[Title/Abstract] OR "chronic osteomyelitis of the jaws"[Title/Abstract] OR "chronic jaw osteomyelitis"[Title/Abstract]) AND ("Microbiota"[Mesh] OR "Biofilms"[Mesh] OR "Actinomyces"[Mesh] OR "Bacteria, Anaerobic"[Mesh] OR "Fungi"[Mesh] OR "Candida"[Mesh] OR "Viruses"[Mesh] OR microbiome[Title/Abstract] OR microbiota[Title/Abstract] OR microflora[Title/Abstract] OR "microbial composition"[Title/Abstract] OR "microbial profile"[Title/Abstract] OR "microbial profiles"[Title/Abstract] OR "bacterial profile"[Title/Abstract] OR "bacterial profiles"[Title/Abstract] OR microbiological[Title/Abstract] OR microbiology[Title/Abstract] OR PCR[Title/Abstract] OR qPCR[Title/Abstract] OR "polymerase chain reaction"[Title/Abstract] OR "molecular detection"[Title/Abstract] OR 16S[Title/Abstract] OR "16S rRNA"[Title/Abstract] OR metagenomic[Title/Abstract] OR metagenomics[Title/Abstract] OR sequencing[Title/Abstract] OR DGGE[Title/Abstract] OR biofilm[Title/Abstract] OR biofilms[Title/Abstract] OR Actinomyces[Title/Abstract] OR actinomycosis[Title/Abstract] OR actinomycotic[Title/Abstract] OR anaerobe[Title/Abstract] OR anaerobes[Title/Abstract] OR "anaerobic bacteria"[Title/Abstract] OR fungus[Title/Abstract] OR fungi[Title/Abstract] OR fungal[Title/Abstract] OR Candida[Title/Abstract] OR mycobiome[Title/Abstract] OR Aspergillus[Title/Abstract] OR mucormycosis[Title/Abstract] OR virus[Title/Abstract] OR viruses[Title/Abstract] OR viral[Title/Abstract] OR virome[Title/Abstract] OR "Epstein-Barr virus"[Title/Abstract] OR EBV[Title/Abstract] OR ((culture[Title/Abstract] OR cultures[Title/Abstract] OR cultured[Title/Abstract] OR isolation[Title/Abstract] OR isolated[Title/Abstract]) AND (microorganism[Title/Abstract] OR microorganisms[Title/Abstract] OR Actinomyces[Title/Abstract] OR anaerobic[Title/Abstract] OR fungus[Title/Abstract] OR fungal[Title/Abstract] OR Candida[Title/Abstract] OR Aspergillus[Title/Abstract])) OR ((histology[Title/Abstract] OR histological[Title/Abstract] OR histopathology[Title/Abstract] OR histopathological[Title/Abstract] OR microscopy[Title/Abstract] OR staining[Title/Abstract]) AND (microorganism[Title/Abstract] OR microorganisms[Title/Abstract] OR Actinomyces[Title/Abstract] OR actinomycotic[Title/Abstract] OR fungal[Title/Abstract] OR Candida[Title/Abstract] OR viral[Title/Abstract] OR EBV[Title/Abstract])))))

Scopus

Platform: Scopus (Elsevier)

Date searched: 25 July 2026

Records retrieved: 292

Search field/interface: TITLE-ABS fields

Exact search strategy

((TITLE-ABS (MRONJ OR BRONJ OR ARONJ OR DRONJ OR {medication-related osteonecrosis of the jaw} OR {medication related osteonecrosis of the jaw} OR {bisphosphonate-related osteonecrosis of the jaw} OR {bisphosphonate related osteonecrosis of the jaw} OR {antiresorptive-related osteonecrosis of the jaw} OR {antiresorptive related osteonecrosis of the jaw} OR {drug-related osteonecrosis of the jaw} OR {drug related osteonecrosis of the jaw} OR ((osteonecrosis W/3 jaw*) AND (medication* OR drug* OR bisphosphonate* OR denosumab OR antiresorptive OR antiangiogenic))) AND TITLE-ABS (microbiome OR microbiota OR microflora OR {oral flora} OR {microbial composition} OR {microbial profile} OR {bacterial profile} OR microbiolog* OR dysbiosis OR 16S OR {16S rRNA} OR metagenom* OR DGGE OR biofilm* OR Actinomyces OR actinomycosis OR actinomycotic OR Schaalia OR anaerob* OR fungus OR fungi OR fungal OR yeast* OR Candida OR candidiasis OR mycobiome OR mycobiota OR Aspergillus OR aspergillosis OR mucormycosis OR virus OR viruses OR viral OR virome OR {Epstein-Barr virus} OR EBV OR EBVMCU OR MTX-LPD OR {methotrexate-associated lymphoproliferative disorder} OR ((PCR OR qPCR OR {polymerase chain reaction} OR sequencing OR NGS OR culture OR cultures OR cultured OR isolation OR isolated OR histology OR histological OR histopathology OR histopathological OR microscopy OR microscopic OR staining OR {special stain} OR {in situ hybridization}) AND (microb* OR bacter* OR Actinomyces OR actinomycosis OR actinomycotic OR anaerob* OR fung* OR yeast* OR Candida OR Aspergillus OR virus OR viral OR EBV)) OR ((infection OR infections OR infected OR colonization OR colonisation) AND (microbiolog* OR microorganism* OR Actinomyces OR actinomycosis OR anaerob* OR Candida OR Aspergillus OR {Epstein-Barr virus} OR EBV OR PCR OR culture)))) OR (TITLE-ABS ({osteoradionecrosis of the jaw} OR {osteoradionecrosis of the jaws} OR {jaw osteoradionecrosis} OR {osteomyelitis of the jaw} OR {osteomyelitis of the jaws} OR {jaw osteomyelitis} OR {chronic osteomyelitis of the jaw} OR {chronic osteomyelitis of the jaws} OR {chronic jaw osteomyelitis}) AND TITLE-ABS (microbiome OR microbiota OR microflora OR {oral flora} OR {microbial composition} OR {microbial profile} OR {bacterial profile} OR microbiolog* OR 16S OR {16S rRNA} OR metagenom* OR DGGE OR biofilm* OR Actinomyces OR actinomycosis OR actinomycotic OR anaerob* OR fungus OR fungi OR fungal OR yeast* OR Candida OR mycobiome OR Aspergillus OR mucormycosis OR virus OR viruses OR viral OR virome OR {Epstein-Barr virus} OR EBV OR ((PCR OR qPCR OR {polymerase chain reaction} OR sequencing OR culture OR cultures OR cultured OR isolation OR isolated OR histology OR histological OR histopathology OR histopathological OR microscopy OR staining) AND (microb* OR bacter* OR Actinomyces OR actinomycosis OR actinomycotic OR anaerob* OR fung* OR Candida OR Aspergillus OR virus OR viral OR EBV)))))

Web of Science Core Collection

Platform: Web of Science Core Collection (Clarivate)

Date searched: 25 July 2026

Records retrieved: 385

Search field/interface: Topic search (TS)

Exact search strategy

TS=((("medication-related osteonecrosis of the jaw" OR "medication related osteonecrosis of the jaw" OR MRONJ OR "bisphosphonate-related osteonecrosis of the jaw" OR "bisphosphonate related osteonecrosis of the jaw" OR BRONJ OR "antiresorptive-related osteonecrosis of the jaw" OR "antiresorptive related osteonecrosis of the jaw" OR ARONJ OR "drug-related osteonecrosis of the jaw" OR "drug related osteonecrosis of the jaw" OR DRONJ OR (("osteonecrosis of the jaw" OR "osteonecrosis of jaws" OR "jaw osteonecrosis") AND (medication OR medications OR drug OR drugs OR bisphosphonate OR bisphosphonates OR denosumab OR antiresorptive OR antiangiogenic))) AND (microbiome OR microbiota OR microflora OR "oral flora" OR "microbial composition" OR "microbial profile" OR "microbial profiles" OR "bacterial profile" OR "bacterial profiles" OR microbiological OR microbiology OR dysbiosis OR PCR OR qPCR OR "polymerase chain reaction" OR "quantitative polymerase chain reaction" OR "real-time PCR" OR "real time PCR" OR "molecular detection" OR "molecular profiling" OR 16S OR "16S rRNA" OR metagenomic OR metagenomics OR sequencing OR "next-generation sequencing" OR "next generation sequencing" OR NGS OR DGGE OR "denaturing gradient gel electrophoresis" OR biofilm OR biofilms OR Actinomyces OR actinomycosis OR actinomycotic OR Schaalia OR "Actinomyces-like organism" OR "Actinomyces-like organisms" OR anaerobe OR anaerobes OR "anaerobic bacteria" OR fungus OR fungi OR fungal OR yeast OR yeasts OR Candida OR candidiasis OR mycosis OR mycobiome OR mycobiota OR Aspergillus OR aspergillosis OR mucormycosis OR virus OR viruses OR viral OR virome OR "Epstein-Barr virus" OR EBV OR EBVMCU OR "methotrexate-associated lymphoproliferative disorder" OR "methotrexate-associated lymphoproliferative disorders" OR MTX-LPD OR ((culture OR cultures OR cultured OR isolation OR isolated) AND (microorganism OR microorganisms OR bacterium OR bacteria OR bacterial OR Actinomyces OR anaerobic OR fungus OR fungal OR Candida OR Aspergillus)) OR ((histology OR histological OR histopathology OR histopathological OR microscopy OR microscopic OR staining OR "special stain" OR "in situ hybridization") AND (microorganism OR microorganisms OR bacterium OR bacteria OR bacterial OR Actinomyces OR actinomycotic OR fungus OR fungal OR Candida OR Aspergillus OR virus OR viral OR EBV)) OR ((infection OR infections OR infected OR colonization OR colonisation) AND (microbiological OR microbiology OR microorganism OR microorganisms OR Actinomyces OR actinomycosis OR anaerobic OR Candida OR Aspergillus OR "Epstein-Barr virus" OR EBV OR PCR OR culture)))) OR (("osteoradionecrosis of the jaw" OR "osteoradionecrosis of the jaws" OR "jaw osteoradionecrosis" OR "osteomyelitis of the jaw" OR "osteomyelitis of the jaws" OR "jaw osteomyelitis" OR "chronic osteomyelitis of the jaw" OR "chronic osteomyelitis of the jaws" OR "chronic jaw osteomyelitis" OR (osteoradionecrosis AND (jaw OR jaws OR mandibular OR maxillary OR maxillofacial))) AND (microbiome OR microbiota OR microflora OR "oral flora" OR "microbial composition" OR "microbial profile" OR "microbial profiles" OR "bacterial profile" OR "bacterial profiles" OR microbiological OR microbiology OR PCR OR qPCR OR "polymerase chain reaction" OR "molecular detection" OR 16S OR "16S rRNA" OR metagenomic OR metagenomics OR sequencing OR DGGE OR biofilm OR biofilms OR Actinomyces OR actinomycosis OR actinomycotic OR anaerobe OR anaerobes OR "anaerobic bacteria" OR fungus OR fungi OR fungal OR Candida OR mycobiome OR Aspergillus OR mucormycosis OR virus OR viruses OR viral OR virome OR "Epstein-Barr virus" OR EBV OR ((culture OR cultures OR cultured OR isolation OR isolated) AND (microorganism OR microorganisms OR Actinomyces OR anaerobic OR fungus OR fungal OR Candida OR Aspergillus)) OR ((histology OR histological OR histopathology OR histopathological OR microscopy OR microscopic OR staining) AND (microorganism OR microorganisms OR Actinomyces OR actinomycotic OR fungal OR Candida OR viral OR EBV)))))

Cochrane Library

Platform: Cochrane Library (Wiley Search Manager)
Date searched: 25 July 2026
Records retrieved: 10
Search field/interface: Title/abstract/keyword fields; near/3 syntax where used

Exact search strategy

((("medication-related osteonecrosis of the jaw" OR "medication related osteonecrosis of the jaw" OR MRONJ OR BRONJ OR ARONJ OR DRONJ OR "bisphosphonate-related osteonecrosis of the jaw" OR "bisphosphonate related osteonecrosis of the jaw" OR "antiresorptive-related osteonecrosis of the jaw" OR "antiresorptive related osteonecrosis of the jaw" OR "drug-related osteonecrosis of the jaw" OR "drug related osteonecrosis of the jaw" OR ((osteonecrosis near/3 jaw*) AND (medication* OR drug* OR bisphosphonate* OR denosumab OR antiresorptive OR antiangiogenic))) AND (microbiome OR microbiota OR microflora OR "oral flora" OR "microbial composition" OR "microbial profile" OR "bacterial profile" OR microbiolog* OR dysbiosis OR 16S OR "16S rRNA" OR metagenom* OR DGGE OR biofilm* OR Actinomyces OR actinomycosis OR actinomycotic OR Schaalia OR anaerob* OR fungus OR fungi OR fungal OR yeast* OR Candida OR candidiasis OR mycobiome OR mycobiota OR Aspergillus OR aspergillosis OR mucormycosis OR virus OR viruses OR viral OR virome OR "Epstein-Barr virus" OR EBV OR EBVMCU OR MTX-LPD OR "methotrexate-associated lymphoproliferative disorder" OR ((PCR OR qPCR OR "polymerase chain reaction" OR sequencing OR NGS OR culture OR cultures OR cultured OR isolation OR isolated OR histology OR histological OR histopathology OR histopathological OR microscopy OR microscopic OR staining OR "special stain" OR "in situ hybridization") AND (microb* OR bacter* OR Actinomyces OR actinomycosis OR actinomycotic OR anaerob* OR fung* OR yeast* OR Candida OR Aspergillus OR virus OR viral OR EBV)) OR ((infection OR infections OR infected OR colonization OR colonisation) AND (microbiolog* OR microorganism* OR Actinomyces OR actinomycosis OR anaerob* OR Candida OR Aspergillus OR "Epstein-Barr virus" OR EBV OR PCR OR culture)))) OR (("osteoradionecrosis of the jaw" OR "osteoradionecrosis of the jaws" OR "jaw osteoradionecrosis" OR "osteomyelitis of the jaw" OR "osteomyelitis of the jaws" OR "jaw osteomyelitis" OR "chronic osteomyelitis of the jaw" OR "chronic osteomyelitis of the jaws" OR "chronic jaw osteomyelitis") AND (microbiome OR microbiota OR microflora OR "oral flora" OR "microbial composition" OR "microbial profile" OR "bacterial profile" OR microbiolog* OR 16S OR "16S rRNA" OR metagenom* OR DGGE OR biofilm* OR Actinomyces OR actinomycosis OR actinomycotic OR anaerob* OR fungus OR fungi OR fungal OR yeast* OR Candida OR mycobiome OR Aspergillus OR mucormycosis OR virus OR viruses OR viral OR virome OR "Epstein-Barr virus" OR EBV OR ((PCR OR qPCR OR "polymerase chain reaction" OR sequencing OR culture OR cultures OR cultured OR isolation OR isolated OR histology OR histological OR histopathology OR histopathological OR microscopy OR staining) AND (microb* OR bacter* OR Actinomyces OR actinomycosis OR actinomycotic OR anaerob* OR fung* OR Candida OR Aspergillus OR virus OR viral OR EBV))))):ti,ab,kw

ClinicalTrials.gov

Platform: ClinicalTrials.gov (NLM registry)
Date searched: 25 July 2026
Records retrieved: 44
Search field/interface: Disease-condition query

Exact search strategy

("medication-related osteonecrosis of the jaw" OR "medication related osteonecrosis of the jaw" OR MRONJ OR "bisphosphonate-related osteonecrosis of the jaw" OR "bisphosphonate related osteonecrosis of the jaw" OR BRONJ OR "antiresorptive-related osteonecrosis of the jaw" OR "antiresorptive related osteonecrosis of the jaw" OR ARONJ OR "drug-related osteonecrosis of the jaw" OR "drug related osteonecrosis of the jaw" OR DRONJ OR (("osteonecrosis of the jaw" OR "jaw osteonecrosis") AND (medication OR drug OR bisphosphonate OR denosumab OR antiresorptive OR antiangiogenic)) OR "osteoradionecrosis of the jaw" OR "jaw osteoradionecrosis" OR "osteomyelitis of the jaw" OR "jaw osteomyelitis" OR "chronic osteomyelitis of the jaw" OR "chronic jaw osteomyelitis") ClinicalTrials.gov was searched to identify ongoing, completed-but-unpublished, or otherwise registered human studies. No registry record contributed eligible microbiological results to the final evidence base.

S1.4 Strategy changes between waves

The two waves are reported separately because their executed strategies were not identical.

Concept or implementation feature	Initial wave (25 June 2026)	Updated wave (25 July 2026)
Disease concepts	MRONJ and historical related terms, with contextual jaw-disease terms in the disease block	MRONJ and historical related terms, with expanded contextual jaw-disease branches
Microbiology concepts	Microbiome, microbiota, bacteria, biofilm, Actinomyces, 16S, metagenomic, and sequencing terms	Added infection, colonization, culture, microbiology, PCR/qPCR, histology, microscopy, in situ hybridization, fungal, yeast, viral, virome, and anaerobe terms
Scopus field syntax	TITLE-ABS-KEY	TITLE-ABS
Cochrane field syntax	Title/abstract/keyword search with :ti,ab,kw	Title/abstract/keyword search with :ti,ab,kw and an expanded strategy structure
Citation searching	Not part of the database query	Backward and forward citation searching conducted separately from the six database/registry queries

The lower Scopus yield in the updated wave reflects the documented change from TITLE-ABS-KEY to TITLE-ABS together with the revised query structure; the updated wave was not expected to be numerically larger solely because its concept coverage was broader. Cochrane retained title/abstract/keyword searching but also used a revised and expanded query structure.

Two-wave record accounting

Accounting item	Initial wave	Updated wave	Combined or cross-wave value
Raw records retrieved	1,895	2,149	4,044
Within-wave duplicate records removed	915	908	1,823
Within-wave unique records	980	1,241	—
Cross-wave overlap	—	—	489
Total duplicates removed after within-wave and cross-wave consolidation	—	—	2,312

Accounting item	Initial wave	Updated wave	Combined or cross-wave value
Combined unique records entering screening	—	—	1,732

The within-wave unique counts are not additive because 489 records were present in both waves. The combined record-level identity is therefore 4,044 raw records minus 2,312 duplicate or overlapping records, yielding 1,732 unique records.

Database	Initial wave (25 Jun)	Updated wave (25 Jul)	Combined total
PubMed/MEDLINE	306	383	689
Embase	404	1,035	1,439
Scopus	817	292	1,109
Web of Science	350	385	735
Cochrane Library	17	10	27
ClinicalTrials.gov	1	44	45
**Total**	**1,895**	**2,149**	**4,044**

S1.5 Additional notes

No language, date, study-design, or publication-type restrictions were applied to the bibliographic searches. ClinicalTrials.gov was searched with the disease-condition strategy to identify registered or potentially unpublished human studies; its records were not assumed to contain microbiological results.

The six-source records were deduplicated within each wave and then matched across waves before the final screening and report-level full-text accounting. Backward and forward citation searching was handled as a separate identification route. The exact executed strategies are reproduced above for each wave; no new combined query was created for this supplement.

S1.6 Abbreviations

ARONJ, antiresorptive-related osteonecrosis of the jaw; BRONJ, bisphosphonate-related osteonecrosis of the jaw; DGGE, denaturing gradient gel electrophoresis; DRONJ, drug-related osteonecrosis of the jaw; EBV, Epstein–Barr virus; Emtree, Embase thesaurus vocabulary; MeSH, Medical Subject Headings; MRONJ, medication-related osteonecrosis of the jaw; MTX-LPD, methotrexate-associated lymphoproliferative disorder; NGS, next-generation sequencing; PCR, polymerase chain reaction; qPCR, quantitative polymerase chain reaction.

Supplementary Table S2. Study-selection accounting, full-text exclusion reasons, and unavailable reports

S2A. Final study-selection accounting

Metric	Value	Unit
Updated six-source raw records	2149	records
Updated-search internal duplicates removed	908	records
Updated-search unique records	1241	records
Overlap with original database	489	records
Newly identified records	752	records
Updated-search records considered potentially eligible after title and abstract screening	214	records
Updated-search records with unclear eligibility after title and abstract screening	12	records
Total updated-search records retained for further assessment	226	records
Updated-search records excluded	1015	records
Combined pathways: unique reports sought	310	reports
Reports retrieved	293	reports
Reports unavailable	17	reports
Database/registry reports included	68	reports
Database/registry reports excluded	225	reports
Citation reports assessed	4	reports
Citation reports included	3	reports
Citation reports excluded	1	reports
Final included reports	71	reports
Final independent studies	67	studies

S2B. Full-text exclusion reasons

Full-text exclusion reason	Reports
No original microbiological data or result	53
Insufficient information to establish an eligible original microbiological finding	49
Wrong or non-eligible condition	38
Treatment or risk-factor only	29
Non-original, abstract-only, or protocol/registry	29
Peripheral contextual evidence	15
Non-human or in-vitro study	10
Mixed population not separable	1
Duplicate or no new data	1
Total	225

S2C. Reports not retrieved

Display title	Original-language or alternative title	Authors	Year	Journal	DOI	PMID	Condition group
Microbial biofilms in osteomyelitis of the jaw and osteonecrosis of the jaw secondary to bisphosphonate therapy.		Sedghizadeh PP, Kumar SK, Gorur A, Schaudinn C, Shuler CF, Costerton JW.	2009	J Am Dent Assoc.	10.14219/jada.archive.2009.0049	19797556	MRONJ
[Bisphosphonate related osteonecrosis of the jaw and infection with Actinomyces].		Arranz Caso JA, Flores Ballester E, Ngo Pombe S, López Pizarro V, Dominguez-Mompello JL, Restoy Lozano A.	2012	Med Clin (Barc).	10.1016/j.medcli.2012.05.039	23103102	MRONJ
Long-term follow-up of jaw osteomyelitis associated with bisphosphonate use in a tertiary-care center.		Pigrau-Serrallach C, Cabral-Galeano E, Almirante-Gragera B, Sordé-Masip R, Rodriguez-Pardo D, Fernandez-Hidalgo N, Larrosa-Escartín N, Bescos-Atín S, Pahissa-Berga A.	2014	Enferm Infecc Microbiol Clin.	10.1016/j.eimc.2013.01.007	23473675	MRONJ
Bisphosphonate-related osteonecrosis of the jaws: a review of 34 cases and evaluation of risk.		Kos M, Kuebler JF, Luczak K, Engelke W.	2010	J Craniomaxillofac Surg.	10.1016/j.jcms.2009.06.005	19592261	MRONJ
Medication-related osteonecrosis of the jaw presenting as cervicofacial actinomycosis	Osteonecrosis mandibular por medicamentos simulando una actinomicosis cervicofacial	Sanín Escobar, A.M.; Mejía Posada, M.I.; Aguilar Gómez, M.; Pérez Madrid, C.; Cadavid Sierra, M.	2022	Piel	10.1016/j.piel.2020.11.004	NR	MRONJ
Epstein - Barr virus positive Mucocutaenous ulcer presenting as medication related osteonecrosis of the jaw		Yassin Alsabbagh, A.; Higgs, N.; Moutasim, K.; Foria, V.; Webb, R.	2021	Advances in Oral and Maxillofacial Surgery	10.1016/j.adoms.2021.100096	NR	MRONJ
[Conditionally pathogenic microorganisms in patients with		Ivaniushko TP, Poliakov KA, Medvedev IA,	2016	Stomatologiia (Mosk).	10.17116/stomat201695144-48	26925566	MRONJ

Display title	Original-language or alternative title	Authors	Year	Journal	DOI	PMID	Condition group
bisphosphonate jaw osteonecrosis].		Shamanaev SV, Trofimov DI, Abramov DD, Balykin RA.					
Actinomyces and bisphosphonate-related osteonecrosis of the jaw		Perna, C.; Ruiz-Bravo, E.; Regojo, R.M.; Tarín, V.; Alonso, S.; Pérez-Míes, B.	2013	Revista Espanola de Patologia	10.1016/j.patol.2013.03.007	NR	MRONJ
A Diagnostic Pitfall: Maxillary Sinus Aspergillosis with Osteomyelitis Mimicking Medication-Related Osteonecrosis of the Jaw in a Bisphosphonate-Treated Patient		Shudo, A.	2026	Journal of Maxillofacial and Oral Surgery	10.1007/s12663-026-03188-1	NR	MRONJ
Role of microbiota and inflammation in osteonecrosis of the jaw		Real, CV; Sayáns, MP; Peñaranda, JMS; Sanchez, FP; Vila, PG; Carrión, AB; Archilla, AR; García, AG	2016	INTERNATIONAL JOURNAL OF CLINICAL AND EXPERIMENTAL PATHOLOGY	NR	NR	MRONJ
Dual Deceivers: Naganishia Diffluens and Aspergillus Ochraceus Masquerading in Post-Radiation Mandibular Osteoradionecrosis		Kumar, P; Tripathi, M; Lal, S; Pandey, M	2025	JOURNAL OF MAXILLOFACIAL & ORAL SURGERY	10.1007/s12663-025-02840-6	NR	Contextual
Osteoradionecrosis of the jaw infected with actinomyces sp: A challenge for clinical practice		Muniz, L.V.; Lacerda, R.P.; Chaves, A.L.F.; Soares, J.M.A.; Leitão, T.J.; Barbosa, L.A.; Dos Santos, H.B.; Ribeiro, R.I.M.A.	2015	Bioscience Journal	10.14393/bj-v31n1a2015-23254; 10.14393/bj-v31n1a2015-31	NR	Contextual
Actinomyces israelii in osteoradionecrosis of the jaws. Histopathologic and immunocytochemical study of five cases.		Happonen RP, Viander M, Pelliniemi L, Aitasalo K.	1983	Oral Surg Oral Med Oral Pathol.	10.1016/0030-4220(83)90374-2	6348637	Contextual
Osteomyelitis of the jaw, simulating sarcoma, myositis ossificans, and actinomycosis		Dixon, O.J.	1947	The Annals of otology, rhinology, and laryngology	NR	20239848	Contextual

Display title	Original-language or alternative title	Authors	Year	Journal	DOI	PMID	Condition group
Two cases of mandibular osteomyelitis caused by Actinomyces		Nagashima, H.; Asada, K.; Yamamoto, H.; Nakayama, A.; Yamaguchi, K.; Nakagawa, Y.; Ishibashi, K.	2006	Oral Therapeutics and Pharmacology	NR	NR	Contextual
[Jaw osteomyelitis microbiology].		Malmström M.	1977	Arsb Odontol Samf Finl.	NR	282531	Contextual
Infectious foci in chronic osteomyelitis of the jaws		Wannfors, K.; Hammarström, L.	1985	International Journal of Oral Surgery	10.1016/s0300-9785(85)80055-7	3936797	Contextual

Abbreviations: DOI, digital object identifier; MRONJ, medication-related osteonecrosis of the jaw; NR, not reported; PMID, PubMed identifier.

Supplementary Table S3. Characteristics of included reports and independent-study clustering

Report	Country	Condition group	Evidence role	Design	Study sample size and unit	Comparator or subgroup details	Diagnostic definition	Specimen	Microbiological method	Primary/companion status	Independent-study cluster
Yamashita 2012	NR	MRONJ	Supportive microbiological	Case report	1 patient	NR	NR	NR	EBER in situ hybridization	Primary report	Study 1
Kimoto 2013	NR	MRONJ	Supportive microbiological	Case report	1 patient	NR	NR	NR	EBER in situ hybridization	Primary report	Study 2
Miyamoto 2017	NR	MRONJ	Supportive microbiological	Case report	1 patient	NR	NR	NR	EBER in situ hybridization	Primary report	Study 3
De Antoni CC 2018	NR	MRONJ	Supportive microbiological	Analytical cross-sectional	16 MRONJ specimens	NR	Unclear	necrotic bone/sequestrum	16S rRNA molecular profiling	Primary report	Study 4
Ogura I 2021	NR	MRONJ	Supportive microbiological	Analytical cross-sectional	7 MRONJ specimens	NR	NR	necrotic bone/sequestrum	histopathology/special staining	Primary report	Study 5
Kanno NI 2024	NR	MRONJ	Supportive microbiological	Case report	1 patient	NR	Unclear	necrotic bone/sequestrum	Histopathology; EBER in situ hybridization; EBV immunohistochemistry	Primary report	Study 6
Yahara H 2020	Japan	MRONJ	Core community-level	Analytical cross-sectional	Initial comparison: 5 MRONJ metagenomic datasets; extended analysis: 10 MRONJ datasets.	Initial comparison: 5 healthy-control metagenomic datasets; extended analysis: 14 healthy-control datasets.	NR	Unstimulated saliva; 1 mL	Shotgun metagenomic sequencing; Illumina HiSeq 2 × 150 bp	Primary report	Study 7

Report	Country	Condition group	Evidence role	Design	Study sample size and unit	Comparator or subgroup details	Diagnostic definition	Specimen	Microbiological method	Primary/companion status	Independent-study cluster
Ari I 2025	NR	MRONJ	Supportive microbiological	Analytical cross-sectional	13 MRONJ patients	NR	NR	necrotic bone/sequestrum	culture; histopathology/special staining	Primary report	Study 8
Jabbour Z 2016	NR	MRONJ	Supportive microbiological	Case series	10 patients with anti-osteoclastic drug-related osteonecrosis of the jaw	NR	Unclear	necrotic bone/sequestrum	DNA checkerboard hybridization with bacterial and fungal genomic probes	Primary report	Study 9
Badros AZ 2021	United States	MRONJ	Core community-level	Cohort	14 patients with BRONJ	28 control participants (microbiome profiling)	NR	Whole unstimulated saliva; serial sampling	16S rRNA gene sequencing; 97% OTU clustering	Primary report	Study 10
Ciobanu GA 2023	NR	MRONJ	Supportive microbiological	Case series	51 MRONJ patients	NR	NR	necrotic bone/sequestrum	culture; histopathology/special staining	Primary report	Study 11
Choi SG 2022	NR	Jaw osteomyelitis	Contextual	Analytical cross-sectional	222 jaw-osteomyelitis patients	NR	NR	necrotic bone/sequestrum	culture; histopathology/special staining	Primary report	Study 12
Li 2023	NR	Osteoradionecrosis	Contextual	Analytical cross-sectional	30 ORN patients	NR	NR	necrotic bone/sequestrum	community sequencing; culture; PCR/qPCR	Primary report	Study 13
Takeda 2021	NR	Osteoradionecrosis	Contextual	Case series	4 ORN specimens	NR	NR	necrotic bone/sequestrum	culture; PCR/qPCR; histopathology/special staining	Primary report	Study 14

Report	Country	Condition group	Evidence role	Design	Study sample size and unit	Comparator or subgroup details	Diagnostic definition	Specimen	Microbiological method	Primary/companion status	Independent-study cluster
Miranda-Rius J 2014	NR	MRONJ	Supportive microbiological	Case report	1 patient	NR	NR	necrotic bone/sequestrum	culture; histopathology/special staining	Primary report	Study 15
Bhavthankar JD 2023	NR	Jaw osteomyelitis	Contextual	Case series	4 patients	NR	NR	necrotic bone/sequestrum	culture	Primary report	Study 16
Sezer B 2017	NR	Jaw osteomyelitis	Contextual	Case series	4 patients	NR	NR	necrotic bone/sequestrum	culture; PCR/qPCR; histopathology/special staining	Primary report	Study 17
Anavi-Lev K 2013	Israel	MRONJ	Supportive microbiological	Cohort	52 BRONJ patients	NR	NR	archived jaw biopsy tissue; archived oral mucosa and jawbone tissue	histopathological Actinomyces assessment	Primary report	Study 18
Kaplan 2009	Israel	MRONJ	Supportive microbiological	Cohort	14 patients with bisphosphonate-associated jaw disease	NR	NR	archived jaw biopsy tissue; archived oral mucosa and jawbone tissue	histopathological Actinomyces assessment	Companion report	Study 18
Crincoli V 2015	Italy	MRONJ	Supportive microbiological	Case series	20 BRONJ patients	NR	Unclear	oral mucosa; necrotic bone fragments; fistula drainage	culture	Primary report	Study 19
Fukuzawa S 2022	Japan	MRONJ	Supportive microbiological	Case report	1 patient	NR	historical BRONJ definition	palatal mucosal biopsy and sequestrum	EBER in situ hybridization	Primary report	Study 20

Report	Country	Condition group	Evidence role	Design	Study sample size and unit	Comparator or subgroup details	Diagnostic definition	Specimen	Microbiological method	Primary/companion status	Independent-study cluster
Koerdt S 2014	Germany	MRONJ	Supportive microbiological	Analytical cross-sectional	23 BRONJ patients	NR	historical BRONJ definition	jaw tissue specimens	histopathology/special staining	Primary report	Study 21
Sugisaki 2024	Japan	MRONJ	Supportive microbiological	Case report	1 patient	NR	historical BRONJ definition	maxillary gingival biopsy and resected necrotic bone	EBER in situ hybridization	Primary report	Study 22
Cerrato A 2021	Italy	MRONJ	Supportive microbiological	Case series	101 MRONJ patients	NR	historical BRONJ definition	surgically excised jaw lesions	histopathological Actinomyces assessment	Primary report	Study 23
He P 2022	United States	MRONJ	Supportive microbiological	Cohort	33 MRONJ patients	NR	partial definition	wound/tissue specimens	culture	Primary report	Study 24
Zirk M 2019	Germany	MRONJ	Supportive microbiological	Cohort	71 MRONJ patients	NR	historical BRONJ definition	osseous lesion-bottom and submucosal specimens	culture	Primary report	Study 25
Seki K 2023	Japan	MRONJ	Supportive microbiological	Case report	1 patient	NR	historical BRONJ definition	excised maxillary bone tissue	histopathological assessment	Primary report	Study 26
Arianpour K 2024	United States	Osteoradionecrosis	Contextual	Case series	24 ORN patients	NR	NR	tissue at rescue-flap surgery	culture; Gram staining	Primary report	Study 27
Longfellow GA 2026	United States	Osteoradionecrosis	Contextual	Cohort	84 ORN patients	NR	NR	clinical osteoradionecrosis cultures	culture	Primary report	Study 28

Report	Country	Condition group	Evidence role	Design	Study sample size and unit	Comparator or subgroup details	Diagnostic definition	Specimen	Microbiological method	Primary/companion status	Independent-study cluster
Hansen 2006 — Actinomyces in infected osteoradionecrosis	Germany	Osteoradionecrosis	Contextual	Case series	31 ORN patients	NR	NR	bone and fistula skin/mucosa biopsies; mandibular bone biopsy	Histopathology with special staining; semi-nested PCR	Primary report	Study 29
Hansen 2006 — IORN follow-up	Germany	Osteoradionecrosis	Contextual	Case series	25 ORN patients	NR	NR	No new microbiological specimen reported; follow-up of the previously biopsied cohort.	No new microbiological testing reported; clinical follow-up linked to prior Actinomyces bone-biopsy findings.	Companion report	Study 29
Zhu 2022	China	Osteoradionecrosis	Contextual	Cohort	152 ORN patients	NR	NR	jaw lesion specimens	culture	Primary report	Study 30
Curi MM 2000	Brazil	Osteoradionecrosis	Contextual	Cohort	50 ORN patients	NR	NR	jawbone biopsy	bone-biopsy histopathology	Primary report	Study 31
Pigrau C 2009	Spain	Jaw osteomyelitis, mixed etiologies	Contextual	Case series	46 jaw-osteomyelitis patients	NR	NR	jaw infection cultures	culture	Primary report	Study 32
Frid P 2009	Norway	Jaw osteomyelitis	Contextual	Case series	5 patients with primary chronic osteomyelitis	NR	NR	mandibular bone biopsy	culture; PCR/qPCR	Primary report	Study 33
Bertrand K 2018	France	Jaw osteomyelitis, mixed etiologies	Contextual	Cohort	56 jaw-osteomyelitis patients	NR	NR	intraoral bone samples	culture	Primary report	Study 34

Report	Country	Condition group	Evidence role	Design	Study sample size and unit	Comparator or subgroup details	Diagnostic definition	Specimen	Microbiological method	Primary/companion status	Independent-study cluster
Liu 1998	Taiwan	Osteoradionecrosis	Contextual	Case report	1 patient	NR	NR	necrotic maxillary bone fragments	microscopy	Primary report	Study 35
Kos M 2010	Germany	MRONJ	Supportive microbiological	Cohort	29 jaw-osteomyelitis patients	18 patients with previous bisphosphonate treatment and 11 control patients without bisphosphonate exposure	historical BRONJ definition	exposed jawbone	histopathological Actinomyces assessment	Primary report	Study 36
Ji 2012	United States	MRONJ	Core community-level	Analytical cross-sectional	20 patients	10 antibiotic-treated versus 10 non-antibiotic BRONJ patients; no healthy comparator	historical BRONJ definition	soft tissue associated with BRONJ lesion	DGGE and Sanger clone sequencing; V4–V5	Primary report	Study 37
Kim HY 2024	South Korea	MRONJ	Core community-level	Analytical cross-sectional	12 patients	12 unaffected-site paired samples	partial definition	oral swab from affected and unaffected sides; submucosal/exudate around lesion	16S rRNA sequencing; V3–V4; Illumina MiSeq; 2 × 300 bp	Primary report	Study 38

Report	Country	Condition group	Evidence role	Design	Study sample size and unit	Comparator or subgroup details	Diagnostic definition	Specimen	Microbiological method	Primary/companion status	Independent-study cluster
Li Q 2020	China	MRONJ	Core community-level	Case-control	9 patients	9 healthy/control oral mucosa specimens	NR	fresh inflammatory tissue next to necrotic bone or sequestrum	Mixed 16S sequencing and culture methods; V3–V4; Illumina MiSeq, paired-end 250 bp	Primary report	Study 39
Goda 2014	Japan	Jaw osteomyelitis	Contextual	Analytical cross-sectional	16 bone specimens from chronic osteomyelitis jaw lesions	5 patients with BRONJ within chronic osteomyelitis; 11 patients with non-BRONJ chronic osteomyelitis/ORN/primary chronic osteomyelitis	partial definition	bone samples from chronic osteomyelitis jaw lesions	16S rRNA amplicon sequencing	Primary report	Study 40
Choi 2025	South Korea	MRONJ	Core community-level	Case-control	39 patients with MRONJ	45 healthy participants	NR	sequestrum / MRONJ-related oral microbial samples	16S rRNA sequencing; Illumina HiSeq 2500	Primary report	Study 41
Hershberger 2024	United States	MRONJ	Supportive microbiological	Case series	39 patients with maxillary MRONJ	subgroups by sinusitis/opacification	NR	MRONJ tissue culture specimens	culture	Primary report	Study 42

Report	Country	Condition group	Evidence role	Design	Study sample size and unit	Comparator or subgroup details	Diagnostic definition	Specimen	Microbiological method	Primary/companion status	Independent-study cluster
Sedghizadeh 2012	United States	MRONJ	Core community-level	Case-control	5 patients with BRONJ	5 non-BRONJ control participants	historical BRONJ definition	saliva and saliva-induced biofilms	Shotgun metagenomic sequencing; V3–V5; 454 GS FLX Titanium pyrosequencing	Primary report	Study 43
Pushalkar 2014	United States	MRONJ	Core community-level	Case-control	15 patients with BRONJ	10 periodontal control participants and 5 bisphosphonate-exposed participants without ONJ	historical BRONJ definition	BRONJ lesion tissue specimens	16S rRNA sequencing; DGGE and clone sequencing	Primary report	Study 44
Kalyan 2015	Germany	MRONJ	Core community-level	Analytical cross-sectional	BAONJ subgroup size not clearly extractable	participants exposed to nitrogen-containing bisphosphonates without ONJ and treatment-naive controls	NR	oral microbiome samples; exact oral site unclear	16S rRNA sequencing; 454 pyrosequencing	Primary report	Study 45
Wei 2012	United States	MRONJ	Core community-level	Case-control	6 patients with BRONJ	6 cancer control participants without BRONJ or bisphosphonate exposure but with infection	NR	jawbone samples	16S rRNA molecular profiling; DGGE and clone sequencing	Primary report	Study 46

Report	Country	Condition group	Evidence role	Design	Study sample size and unit	Comparator or subgroup details	Diagnostic definition	Specimen	Microbiological method	Primary/companion status	Independent-study cluster
Erovigni FM 2026	Italy	MRONJ	Core community-level	Case-control	18 patients with MRONJ	21 drug-exposed control participants without MRONJ; 16 healthy participants	NR	saliva	16S rRNA sequencing; multiple amplicon regions; microbiome panel/NGS	Primary report	Study 47
Kövér Z 2025	Hungary	MRONJ	Core community-level	Case-control	35 patients with MRONJ	35 healthy extraction controls; 5 + 5 specimens selected for 16S	partial definition	surgically removed bone samples	Mixed 16S sequencing and culture methods; V3–V4; Illumina MiSeq	Primary report	Study 48
Hallmer 2017	Sweden	MRONJ	Core community-level	Analytical cross-sectional	18 necrotic bone samples	11 visually healthy bone samples; 2 healthy control participants	NR	necrotic bone center and visually healthy bone	16S rRNA molecular profiling; V3–V5; 454 GS Junior pyrosequencing	Primary report	Study 49
De Bruyn 2018	Belgium	MRONJ	Supportive microbiological	Cohort	38 patients with MRONJ	29 orally healthy controls	partial definition	swabs at infected MRONJ lesion margin with visible pus	Targeted real-time PCR using TaqMan assays	Primary report	Study 50
Panya 2017	Germany	MRONJ	Supportive microbiological	Analytical cross-sectional	95 patients with MRONJ	NR	NR	MRONJ bone samples	culture; PCR/qPCR	Primary report	Study 51
Kövér Z 2023	Hungary	MRONJ	Supportive microbiological	Case series	3 MRONJ patients	NR	NR	necrotic bone samples	anaerobic culture; molecular identification	Primary report	Study 52

Report	Country	Condition group	Evidence role	Design	Study sample size and unit	Comparator or subgroup details	Diagnostic definition	Specimen	Microbiological method	Primary/companion status	Independent-study cluster
Ewald 2021	Germany	MRONJ	Supportive microbiological	Case series	98 patients with MRONJ	NR	partial definition	necrotic bone	culture; PCR/qPCR	Primary report	Study 53
Kusumoto 2025	Japan	MRONJ	Supportive microbiological	Analytical cross-sectional	55 patients with MRONJ	22 patients with ORN	partial definition	deep tissue culture specimens	culture	Primary report	Study 54
Russmueller 2016	Austria	MRONJ	Supportive microbiological	Cohort	111 patients with MRONJ	NR	partial definition	deep biopsies of necrotic bone	culture; histopathology/special staining	Primary report	Study 55
Micheletti 2022	United States	MRONJ	Supportive microbiological	Case report	1 patient with MRONJ; 3 bone specimens (2 lesion-associated and 1 unaffected-site)	NR	partial definition	NR	BSE-SEM/SE-SEM; resin-cast etching	Primary report	Study 56
Schubert 2021	Austria	MRONJ	Supportive microbiological	Cohort	52 patients with MRONJ	NR	partial definition	NR	culture	Primary report	Study 57
Ibrahim N 2022	Malaysia	MRONJ	Supportive microbiological	Case series	8 ONJ cases (3 MRONJ, 5 ORN)	NR	partial definition	NR	culture; histopathology/special staining; PCR/qPCR	Primary report	Study 58
Naik 2009	United States	MRONJ	Supportive microbiological	Case report	1 patient	NR	NR	NR	culture; histopathology/special staining	Primary report	Study 59

Report	Country	Condition group	Evidence role	Design	Study sample size and unit	Comparator or subgroup details	Diagnostic definition	Specimen	Microbiological method	Primary/companion status	Independent-study cluster
Hansen 2006 — BP-ONJ versus IORN	Germany	MRONJ	Supportive microbiological	Analytical cross-sectional	8 patients with bisphosphonate-associated osteonecrosis	10 patients with infected osteoradionecrosis	NR	necrotic jaw bone tissue	Histomorphological assessment	Primary report	Study 60
Hansen 2007 — Actinomycosis of the jaws	Germany	MRONJ	Supportive microbiological	Analytical cross-sectional	45 total patients	Relevant bisphosphonate-associated osteonecrosis and IORN subgroups were not separately extractable from the accessible report.	NR	necrotic jaw bone tissue	Histopathological assessment; PCR on selected tissue specimens	Companion report	Study 60
Carmagnola 2013	Italy	MRONJ	Supportive microbiological	Analytical cross-sectional	14 patients with BONJ	2 patients with ORN	historical BRONJ definition	bone tissue samples	histopathology/special staining	Primary report	Study 61
Sedghizadeh 2008	United States	MRONJ	Supportive microbiological	Case series	4 patients with BRONJ	NR	historical BRONJ definition	affected bone samples; NR	direct biofilm imaging; scanning electron microscopy	Primary report	Study 62
Wanger 2013	United States	MRONJ	Supportive microbiological	Case series	4 patients with BRONJ	NR	historical BRONJ definition	affected bone samples; NR	direct biofilm imaging; scanning electron microscopy	Companion report	Study 62

Report	Country	Condition group	Evidence role	Design	Study sample size and unit	Comparator or subgroup details	Diagnostic definition	Specimen	Microbiological method	Primary/companion status	Independent-study cluster
Santana DA 2025	Brazil; Guatemala	MRONJ	Supportive microbiological	Case series	22 patients with MRONJ	NR	partial definition	histologic slides / jaw lesion tissue	histopathology/special staining	Primary report	Study 63
Aftimos V 2014	Lebanon	MRONJ	Supportive microbiological	Case series	18 patients with BRONJ	NR	historical BRONJ definition	curetted jaw necrotic lesions	histopathology/special staining	Primary report	Study 64
Brody 2022	Hungary	MRONJ	Supportive microbiological	Case series	112 samples	NR	NR	histological MRONJ jaw samples	histopathology/special staining	Primary report	Study 65
Gallay 2013	France	MRONJ	Supportive microbiological	Case report	1 patient	NR	NR	surgical bone sampling	culture	Primary report	Study 66
Gaetti-Jardim Júnior 2010	Brazil	Jaw osteomyelitis	Contextual	Case series	22 specimens (bone sequestra, purulent secretion, and granulomatous tissue biopsies)	NR	NR	bone sequestra; purulent secretion; granulomatous tissue biopsies	anaerobic culture; PCR	Primary report	Study 67

Abbreviations: BAONJ, bisphosphonate-associated osteonecrosis of the jaw; BONJ, bisphosphonate-associated osteonecrosis of the jaw; BP-ONJ, bisphosphonate-associated osteonecrosis of the jaw; BRONJ, bisphosphonate-related osteonecrosis of the jaw; BSE-SEM, backscattered-electron scanning electron microscopy; DGGE, denaturing gradient gel electrophoresis; EBER, Epstein–Barr virus-encoded RNA; EBV, Epstein–Barr virus; IORN, infected osteoradionecrosis; MRONJ, medication-related osteonecrosis of the jaw; NGS, next-generation sequencing; NR, not reported; ONJ, osteonecrosis of the jaw; ORN, osteoradionecrosis; OTU, operational taxonomic unit; PCR, polymerase chain reaction; qPCR, quantitative polymerase chain reaction; SE-SEM, secondary-electron scanning electron microscopy.

Supplementary Table S4A. Clinical variables

Study	Author–year	Diagnostic definition	Exposed/probeable-bone duration	Exclusion of previous jaw radiotherapy	Exclusion of metastatic disease to the jaw	Underlying indication	Medication class	Medication route	Treatment duration	MRONJ stage	Lesion location	Oral hygiene	Periodontal disease	Prior extraction or dental surgery	Prior debridement	Smoking	Immunosuppression
Study 1	Yamashita 2012	NR	NR	NR	NR	NR	NR	NR	NR	NR	NR	NR	NR	NR	NR	NR	NR
Study 2	Kimoto 2013	NR	NR	NR	NR	NR	NR	NR	NR	NR	NR	NR	NR	NR	NR	NR	NR
Study 3	Miyamoto 2017	NR	NR	NR	NR	Reported	Reported	Reported	NR	NR	Reported	NR	NR	Reported	NR	NR	Reported
Study 4	De Antoni CC 2018	Unclear	NR	NR	NR	Reported	Reported	Reported	NR	NR	Reported	NR	NR	Reported	NR	NR	NR
Study 5	Ogura I 2021	NR	NR	NR	NR	NR	NR	NR	NR	NR	NR	NR	NR	NR	NR	NR	NR
Study 6	Kanno NI 2024	Unclear	NR	NR	NR	Reported	Reported	Reported	Reported	Reported	Reported	Reported	Reported	Reported	Reported	NR	Reported
Study 7	Yahara H 2020	NR	NR	NR	NR	NR	NR	NR	NR	NR	NR	NR	NR	NR	NR	NR	NR
Study 8	Ari I 2025	NR	NR	NR	NR	NR	NR	NR	NR	NR	NR	NR	NR	NR	NR	NR	NR
Study 9	Jabbour Z 2016	Unclear	NR	NR	NR	Reported	Reported	Reported	NR	Reported	Reported	NR	Reported	Reported	NR	NR	NR
Study 10	Badros AZ 2021	NR	NR	NR	NR	NR	NR	NR	NR	NR	NR	NR	NR	NR	NR	NR	NR
Study 11	Ciobanu GA 2023	NR	NR	NR	NR	NR	NR	NR	NR	NR	NR	NR	NR	NR	NR	NR	NR

Study	Author–year	Diagnostic definition	Exposed/probeable-bone duration	Exclusion of previous jaw radiotherapy	Exclusion of metastatic disease to the jaw	Underlying indication	Medication class	Medication route	Treatment duration	MRONJ stage	Lesion location	Oral hygiene	Periodontal disease	Prior extraction or dental surgery	Prior debridement	Smoking	Immunosuppression
Study 15	Miranda-Rius J 2014	NR	NR	NR	NR	NR	NR	NR	NR	NR	NR	NR	NR	NR	NR	NR	NR
Study 18	Anavi-Lev K 2013	NR	Unclear	Unclear	Unclear	Reported	Reported	Reported	Reported	Reported	Reported	NR	NR	Reported	Reported	NR	Reported
Study 19	Crincoli V 2015	Unclear	Unclear	Unclear	Unclear	NR	NR	NR	NR	NR	Reported	NR	NR	NR	NR	NR	NR
Study 20	Fukuzawa S 2022	Reported	NR	NR	NR	Reported	Reported	Reported	NR	NR	Reported	NR	Reported	Reported	Reported	NR	Reported
Study 21	Koerdt S 2014	Reported	NR	NR	NR	Reported	Reported	Reported	NR	NR	Reported	NR	NR	Reported	NR	NR	Reported
Study 22	Sugisaki 2024	Reported	NR	NR	NR	Reported	Reported	Reported	Reported	Reported	Reported	NR	NR	Reported	NR	NR	Reported
Study 23	Cerrato A 2021	Reported	NR	NR	NR	Reported	Reported	Reported	Reported	NR	Reported	NR	Reported	Reported	NR	NR	Reported
Study 24	He P 2022	Reported	NR	NR	NR	Reported	Reported	Reported	NR	Reported	Reported	NR	Reported	Reported	NR	Reported	NR
Study 25	Zirk M 2019	Reported	NR	NR	NR	Reported	Reported	Reported	NR	Reported	Reported	Reported	Reported	Reported	Reported	NR	NR
Study 26	Seki K 2023	Reported	NR	NR	NR	Reported	Reported	Reported	NR	Reported	Reported	NR	Reported	Reported	Reported	Reported	NR
Study 36	Kos M 2010	Reported	NR	NR	NR	Reported	Reported	Reported	NR	NR	Reported	NR	NR	Reported	NR	NR	Reported
Study 37	Ji 2012	Reported	Reported	Reported	NR	Reported	Reported	Reported	NR	Reported	Reported	Reported	Reported	Reported	Reported	Reported	Reported

Study	Author–year	Diagnostic definition	Exposed/probeable-bone duration	Exclusion of previous jaw radiotherapy	Exclusion of metastatic disease to the jaw	Underlying indication	Medication class	Medication route	Treatment duration	MRONJ stage	Lesion location	Oral hygiene	Periodontal disease	Prior extraction or dental surgery	Prior debridement	Smoking	Immunosuppression
Study 38	Kim HY 2024	Reported	NR	NR	NR	Reported	Reported	Reported	NR	Reported	Reported	NR	Reported	Reported	NR	Reported	NR
Study 39	Li Q 2020	NR	NR	NR	NR	Reported	Reported	Reported	NR	Reported	Reported	NR	Reported	Reported	NR	NR	Reported
Study 41	Choi 2025	NR	NR	NR	NR	Reported	Reported	Reported	Reported	Reported	Reported	Reported	Reported	Reported	NR	NR	Reported
Study 42	Hershberger 2024	NR	NR	NR	NR	Reported	Reported	Reported	NR	Reported	Reported	NR	Reported	Reported	Reported	Reported	NR
Study 43	Sedghizadeh 2012	Reported	NR	NR	NR	Reported	Reported	Reported	Reported	Reported	Reported	NR	NR	NR	NR	NR	NR
Study 44	Pushalkar 2014	Reported	Reported	Reported	NR	Reported	Reported	Reported	NR	NR	Reported	Reported	Reported	Reported	NR	NR	Reported
Study 45	Kalyan 2015	NR	NR	NR	NR	Reported	Reported	Reported	Reported	NR	Reported	NR	Reported	Reported	NR	Reported	Reported
Study 46	Wei 2012	NR	NR	NR	NR	Reported	Reported	Reported	NR	NR	Reported	NR	Reported	Reported	Reported	NR	NR
Study 47	Erovigni FM 2026	NR	NR	NR	NR	Reported	Reported	Reported	Reported	Reported	Reported	Reported	Reported	Reported	Reported	Reported	NR
Study 48	Kövér Z 2025	Reported	Reported	NR	NR	Reported	Reported	Reported	NR	Reported	Reported	Reported	NR	Reported	Reported	Reported	Reported
Study 49	Hallmer 2017	NR	NR	NR	NR	Reported	Reported	Reported	Reported	Reported	Reported	NR	Reported	Reported	NR	NR	NR
Study 50	De Bruyn 2018	Reported	Reported	Reported	NR	Reported	Reported	Reported	NR	Reported	Reported	Reported	Reported	Reported	NR	Reported	Reported

Study	Author–year	Diagnostic definition	Exposed/probeable-bone duration	Exclusion of previous jaw radiotherapy	Exclusion of metastatic disease to the jaw	Underlying indication	Medication class	Medication route	Treatment duration	MRONJ stage	Lesion location	Oral hygiene	Periodontal disease	Prior extraction or dental surgery	Prior debridement	Smoking	Immunosuppression
Study 51	Panya 2017	NR	NR	NR	NR	Reported	Reported	Reported	NR	Reported	Reported	NR	Reported	Reported	NR	Reported	Reported
Study 52	Kövér Z 2023	NR	NR	NR	NR	Reported	Reported	Reported	Reported	Reported	Reported	Reported	NR	Reported	NR	Reported	NR
Study 53	Ewald 2021	Reported	NR	Reported	NR	Reported	Reported	Reported	NR	Reported	Reported	NR	Reported	Reported	NR	NR	Reported
Study 54	Kusumoto 2025	Reported	Reported	NR	NR	Reported	Reported	Reported	Reported	Reported	Reported	NR	NR	Reported	Reported	Reported	Reported
Study 55	Russmueller 2016	Reported	Reported	NR	NR	Reported	Reported	Reported	NR	Reported	Reported	NR	NR	Reported	NR	Reported	Reported
Study 56	Micheletti 2022	Reported	Reported	NR	Reported	Reported	Reported	Reported	NR	NR	Reported	NR	Reported	Reported	NR	Reported	NR
Study 57	Schubert 2021	Reported	Reported	NR	NR	Reported	Reported	Reported	Reported	Reported	Reported	NR	NR	Reported	Reported	Reported	Reported
Study 58	Ibrahim N 2022	Reported	Reported	Reported	NR	Reported	Reported	Reported	Reported	Reported	Reported	Reported	NR	Reported	Reported	NR	Reported
Study 59	Naik 2009	NR	NR	NR	NR	Reported	Reported	Reported	Reported	NR	Reported	NR	NR	Reported	Reported	NR	NR
Study 60	Hansen et al. 2006/2007 (MRONJ cluster)	NR	NR	NR	NR	Reported	Reported	Reported	Reported	NR	Reported	NR	NR	Reported	NR	NR	Reported
Study 61	Carmagnola 2013	Reported	Reported	Reported	NR	Reported	Reported	Reported	Reported	Reported	Reported	Reported	NR	Reported	Reported	NR	NR

Study	Author–year	Diagnostic definition	Exposed/probeable-bone duration	Exclusion of previous jaw radiotherapy	Exclusion of metastatic disease to the jaw	Underlying indication	Medication class	Medication route	Treatment duration	MRONJ stage	Lesion location	Oral hygiene	Periodontal disease	Prior extraction or dental surgery	Prior debridement	Smoking	Immunosuppression
Study 62	Sedghizadeh 2008	Reported	NR	NR	NR	Reported	Reported	Reported	NR	NR	Reported	NR	NR	Reported	Reported	NR	NR
Study 63	Santana DA 2025	Reported	Reported	NR	NR	Reported	Reported	Reported	NR	NR	Reported	NR	NR	Reported	NR	NR	NR
Study 64	Aftimos V 2014	Reported	NR	NR	NR	Reported	Reported	Reported	NR	NR	Reported	Reported	NR	Reported	NR	NR	NR
Study 65	Brody 2022	NR	Reported	NR	NR	Reported	Reported	Reported	NR	Reported	Reported	NR	NR	Reported	NR	NR	NR
Study 66	Gallay 2013	NR	NR	NR	NR	Reported	Reported	Reported	NR	NR	Reported	NR	NR	Reported	Reported	NR	Reported

Reported indicates that the variable was explicitly reported; NR, not reported; Unclear indicates insufficient information to determine reporting status. MRONJ, medication-related osteonecrosis of the jaw.

Supplementary Table S4B. Antibiotic and sampling variables

Study	Author–year	Recent antibiotic exposure	Antibiotic timing	Specimen timing	Sampling site
Study 1	Yamashita 2012	NR	NR	NR	NR
Study 2	Kimoto 2013	NR	NR	NR	NR
Study 3	Miyamoto 2017	NR	NR	NR	Reported
Study 4	De Antoni CC 2018	NR	NR	NR	Reported
Study 5	Ogura I 2021	NR	NR	NR	NR
Study 6	Kanno NI 2024	NR	NR	Reported	Reported
Study 7	Yahara H 2020	NR	NR	NR	NR
Study 8	Ari I 2025	NR	NR	NR	NR
Study 9	Jabbour Z 2016	Reported	Reported	Reported	Reported
Study 10	Badros AZ 2021	NR	NR	NR	NR
Study 11	Ciobanu GA 2023	NR	NR	NR	NR
Study 15	Miranda-Rius J 2014	NR	NR	NR	NR
Study 18	Anavi-Lev K 2013	Reported	Reported	Reported	Reported
Study 19	Crincoli V 2015	NR	NR	Reported	Reported
Study 20	Fukuzawa S 2022	NR	NR	NR	Reported
Study 21	Koerdt S 2014	NR	NR	NR	Reported
Study 22	Sugisaki 2024	NR	NR	NR	Reported
Study 23	Cerrato A 2021	NR	NR	Reported	Reported
Study 24	He P 2022	Reported	Reported	NR	Reported
Study 25	Zirk M 2019	Reported	Reported	NR	Reported
Study 26	Seki K 2023	NR	NR	NR	Reported
Study 36	Kos M 2010	NR	NR	Reported	Reported
Study 37	Ji 2012	Reported	Reported	Reported	Reported
Study 38	Kim HY 2024	Reported	Reported	Reported	Reported
Study 39	Li Q 2020	Reported	Reported	Reported	Reported
Study 41	Choi 2025	Reported	NR	NR	Reported

Study	Author–year	Recent antibiotic exposure	Antibiotic timing	Specimen timing	Sampling site
Study 42	Hershberger 2024	Reported	NR	NR	Reported
Study 43	Sedghizadeh 2012	Reported	Reported	NR	Reported
Study 44	Pushalkar 2014	Reported	Reported	Reported	Reported
Study 45	Kalyan 2015	Reported	Reported	NR	Reported
Study 46	Wei 2012	Reported	NR	Reported	Reported
Study 47	Erovigni FM 2026	Reported	Reported	Reported	Reported
Study 48	Kövér Z 2025	Reported	Reported	Reported	Reported
Study 49	Hallmer 2017	Reported	Reported	NR	Reported
Study 50	De Bruyn 2018	Reported	Reported	Reported	Reported
Study 51	Panya 2017	Reported	NR	NR	Reported
Study 52	Kövér Z 2023	Reported	NR	Reported	Reported
Study 53	Ewald 2021	Reported	Reported	Reported	Reported
Study 54	Kusumoto 2025	Reported	Reported	Reported	Reported
Study 55	Russmueller 2016	Reported	Reported	Reported	Reported
Study 56	Micheletti 2022	NR	NR	Reported	Reported
Study 57	Schubert 2021	Reported	Reported	NR	Reported
Study 58	Ibrahim N 2022	Reported	Reported	NR	Reported
Study 59	Naik 2009	Reported	Reported	NR	Reported
Study 60	Hansen et al. 2006/2007 (MRONJ cluster)	NR	NR	NR	Reported
Study 61	Carmagnola 2013	NR	NR	Reported	Reported
Study 62	Sedghizadeh 2008	Reported	NR	Reported	Reported
Study 63	Santana DA 2025	NR	NR	NR	Reported
Study 64	Aftimos V 2014	NR	NR	NR	Reported
Study 65	Brody 2022	Reported	Reported	Reported	Reported
Study 66	Gallay 2013	Reported	NR	Reported	Reported

Reported indicates that the variable was explicitly reported; NR, not reported; Unclear indicates insufficient information to determine reporting status. MRONJ, medication-related osteonecrosis of the jaw.

Supplementary Table S5. Design-specific JBI appraisal and STORMS-informed reporting assessment

S5A. Design-specific JBI appraisal

JBI item-level judgements were finalized after dual human verification of all reassessed items.

S5A status definitions and summary

Status definitions: Yes = explicitly supported; No = explicitly not met; Unclear = insufficient information; NA = not applicable to the design-specific checklist. The appraisal unit is the independent study. No total score or overall grade was calculated.

JBI summary: Yes 445; No 19; Unclear 160; NA 2; total 626.

Analytical cross-sectional

Item legend: Item 1: clear inclusion criteria; Item 2: subjects and setting; Item 3: exposure measurement; Item 4: objective condition criteria; Item 5: confounders identified; Item 6: confounding strategies; Item 7: outcome measurement; Item 8: statistical analysis

Study	Author–year	Item 1	Item 2	Item 3	Item 4	Item 5	Item 6	Item 7	Item 8
Study 4	De Antoni CC 2018	Unclear	Yes	Yes	Yes	Unclear	Unclear	Yes	Unclear
Study 5	Ogura I 2021	Unclear	Yes	Yes	Yes	Unclear	Unclear	Yes	Yes
Study 7	Yahara H 2020	Yes	Yes	Yes	Yes	Yes	Yes	Yes	Yes
Study 8	Ari I 2025	Yes	Yes	Yes	Unclear	Yes	Unclear	Yes	Yes
Study 12	Choi SG 2022	Yes	Yes	Yes	Unclear	Yes	Yes	Yes	Yes
Study 13	Li 2023	Unclear	Yes	Yes	Unclear	Unclear	Yes	Yes	Yes
Study 21	Koerdt S 2014	Yes	Yes	Yes	Yes	Unclear	Unclear	Yes	Yes
Study 37	Ji 2012	Unclear	Yes	Yes	Yes	Unclear	No	Yes	Yes
Study 38	Kim HY 2024	Yes	Yes	Yes	Yes	Yes	Yes	Yes	Yes
Study 40	Goda 2014	Unclear	Yes	Yes	Yes	Unclear	Unclear	Yes	Yes
Study 45	Kalyan 2015	Yes	Yes	Yes	Yes	Yes	Yes	Yes	Yes
Study 49	Hallmer 2017	Yes	Yes	Yes	Yes	Unclear	Unclear	Yes	Yes
Study 51	Panya 2017	Yes	Yes	Yes	Yes	Yes	Unclear	Yes	Yes
Study 54	Kusumoto 2025	Yes	Yes	Yes	Yes	Yes	Yes	Yes	Yes
Study 60	Hansen et al. 2006/2007 (MRONJ cluster)	Unclear	Yes	Yes	Yes	Unclear	Unclear	Yes	Unclear

Study	Author–year	Item 1	Item 2	Item 3	Item 4	Item 5	Item 6	Item 7	Item 8
Study 61	Carmagnola 2013	Unclear	Yes	Yes	Yes	Yes	No	Yes	No

Case-control

Item legend: Item 1: comparability; Item 2: matching; Item 3: case/control criteria; Item 4: exposure measurement; Item 5: same exposure measurement; Item 6: confounders; Item 7: confounding strategies; Item 8: outcome assessment; Item 9: exposure period; Item 10: statistical analysis

Study	Author–year	Item 1	Item 2	Item 3	Item 4	Item 5	Item 6	Item 7	Item 8	Item 9	Item 10
Study 39	Li Q 2020	No	Unclear	Unclear	Yes	Yes	Unclear	Unclear	Yes	Yes	Yes
Study 41	Choi 2025	No	Yes	Unclear	Yes	Yes	Yes	No	Yes	Yes	Yes
Study 43	Sedghizadeh 2012	Yes	Yes	Yes	Yes	Yes	Yes	Yes	Yes	Yes	Yes
Study 44	Pushalkar 2014	No	Unclear	Yes	Yes	Yes	Yes	No	Yes	Yes	Yes
Study 46	Wei 2012	No	Unclear	Unclear	Yes	Yes	Unclear	Unclear	Yes	Yes	Yes
Study 47	Erovigni FM 2026	Yes	Unclear	Yes	Yes	Yes	Yes	Yes	Yes	Yes	Yes
Study 48	Kövér Z 2025	No	No	Yes	Yes	Yes	Yes	No	Yes	Yes	No

Cohort

Item legend: Item 1: groups and population; Item 2: exposure assignment; Item 3: exposure measurement; Item 4: confounders; Item 5: confounding strategies; Item 6: baseline outcome status; Item 7: outcome measurement; Item 8: follow-up time; Item 9: follow-up completeness; Item 10: incomplete follow-up strategies; Item 11: statistical analysis

Study	Author–year	Item 1	Item 2	Item 3	Item 4	Item 5	Item 6	Item 7	Item 8	Item 9	Item 10	Item 11
Study 10	Badros AZ 2021	Yes	Yes	Yes	Yes	Unclear	Yes	Yes	Yes	Unclear	Unclear	Yes
Study 18	Anavi-Lev K 2013	Yes	Unclear	Yes	Yes	Unclear	Unclear	Yes	Unclear	Yes	Yes	Yes
Study 24	He P 2022	Yes	Unclear	Yes	Yes	Unclear	Unclear	Yes	Unclear	Unclear	Unclear	Yes

Study	Author–year	Item 1	Item 2	Item 3	Item 4	Item 5	Item 6	Item 7	Item 8	Item 9	Item 10	Item 11
Study 25	Zirk M 2019	Yes	Unclear	Yes	Yes	Yes	Unclear	Yes	Unclear	Unclear	Unclear	Yes
Study 28	Longfellow GA 2026	Yes	Unclear	Unclear	Yes	Yes	Unclear	Yes	Unclear	Unclear	Unclear	Yes
Study 30	Zhu 2022	Yes	Unclear	Unclear	Yes	Unclear	Unclear	Yes	Unclear	Unclear	Unclear	Yes
Study 31	Curi MM 2000	Yes	Yes	Yes	Yes	Unclear	Unclear	Yes	Unclear	Unclear	Unclear	Yes
Study 34	Bertrand K 2018	Yes	Unclear	Yes	Yes	Yes	Yes	Yes	Unclear	Unclear	Yes	Yes
Study 36	Kos M 2010	Yes	Unclear	Yes	Yes	Unclear	Unclear	Yes	Unclear	Unclear	Unclear	Yes
Study 50	De Bruyn 2018	No	Yes	Yes	Yes	No	NA	Yes	Yes	No	No	Yes
Study 55	Russmueller 2016	Yes	Yes	Yes	Yes	No	NA	Yes	Unclear	Unclear	Unclear	Yes
Study 57	Schubert 2021	Yes	Yes	Yes	Yes	No	Yes	Yes	Unclear	Unclear	Yes	Yes

Case series

Item legend: Item 1: inclusion criteria; Item 2: condition measurement; Item 3: condition identification; Item 4: consecutive inclusion; Item 5: complete inclusion; Item 6: demographics; Item 7: clinical information; Item 8: outcomes/follow-up; Item 9: reporting site/context; Item 10: statistical analysis

Study	Author–year	Item 1	Item 2	Item 3	Item 4	Item 5	Item 6	Item 7	Item 8	Item 9	Item 10
Study 9	Jabbour Z 2016	Yes	Yes	Yes	Unclear	Unclear	Yes	Yes	Yes	Yes	Yes
Study 11	Ciobanu GA 2023	Yes	Yes	Yes	Unclear	Unclear	Yes	Yes	Yes	Yes	Yes
Study 14	Takeda 2021	Yes	Yes	Yes	Yes	Unclear	Yes	Yes	Yes	Yes	Unclear
Study 16	Bhavthankar JD 2023	Unclear	Yes	Yes	Unclear	Unclear	Yes	Yes	Yes	Yes	Unclear

Study	Author–year	Item 1	Item 2	Item 3	Item 4	Item 5	Item 6	Item 7	Item 8	Item 9	Item 10
Study 17	Sezer B 2017	Unclear	Yes	Yes	Unclear	Unclear	Yes	Yes	Yes	Yes	Unclear
Study 19	Crincoli V 2015	Yes	Unclear	Yes	Unclear	Unclear	Yes	Yes	Yes	Yes	Unclear
Study 23	Cerrato A 2021	Unclear	Yes	Yes	Yes	Unclear	Unclear	Yes	Yes	Yes	Unclear
Study 27	Arianpour K 2024	Unclear	Yes	Yes	Unclear	Unclear	Yes	Yes	Yes	Yes	Unclear
Study 29	Hansen et al. 2006 (ORN cluster)	Unclear	Yes	Yes	Unclear	Unclear	Yes	Yes	Yes	Unclear	Unclear
Study 32	Pigrau C 2009	Unclear	Yes	Unclear	Unclear	Unclear	Yes	Yes	Yes	Yes	Unclear
Study 33	Frid P 2009	Yes	Yes	Yes	Unclear	Unclear	Yes	Unclear	Unclear	Yes	Unclear
Study 42	Hershberger 2024	Yes	Yes	Yes	Unclear	Yes	Yes	Yes	Yes	Yes	Yes
Study 52	Kövér Z 2023	Unclear	Yes	Yes	Unclear	Unclear	Yes	Yes	Yes	Yes	Yes
Study 53	Ewald 2021	Yes	Yes	Yes	Unclear	Unclear	Yes	Yes	Yes	Yes	Yes
Study 58	Ibrahim N 2022	Yes	Yes	Yes	Unclear	Unclear	Yes	Yes	Yes	Yes	Yes
Study 62	Sedghizadeh 2008	Yes	Yes	Yes	Unclear	Unclear	Yes	Yes	Yes	Yes	Yes
Study 63	Santana DA 2025	Yes	Yes	Yes	Unclear	Yes	Yes	Yes	Yes	Yes	Yes
Study 64	Aftimos V 2014	Yes	Unclear	Yes	Unclear	Yes	Yes	Unclear	Yes	Yes	Yes
Study 65	Brody 2022	Yes	Yes	Yes	Unclear	Yes	Yes	Yes	Yes	Yes	Yes
Study 67	Gaetti-Jardim Júnior 2010	Yes	Yes	Yes	Unclear	Unclear	Yes	Yes	Yes	Yes	Yes

Case report

Item legend: Item 1: demographics; Item 2: history/timeline; Item 3: current condition; Item 4: diagnostic assessment; Item 5: intervention; Item 6: post-intervention condition; Item 7: adverse/unanticipated events; Item 8: takeaway lessons

Study	Author–year	Item 1	Item 2	Item 3	Item 4	Item 5	Item 6	Item 7	Item 8
Study 1	Yamashita 2012	Yes	Yes	Yes	Yes	Yes	Yes	Unclear	Yes
Study 2	Kimoto 2013	Yes	Yes	Yes	Yes	Yes	Yes	Unclear	Yes
Study 3	Miyamoto 2017	Unclear	Yes	Yes	Yes	Yes	Unclear	Unclear	Yes
Study 6	Kanno NI 2024	Yes	Yes	Yes	Yes	Yes	Yes	Yes	Yes
Study 15	Miranda-Rius J 2014	Yes	Yes	Yes	Yes	Yes	Yes	Unclear	Yes
Study 20	Fukuzawa S 2022	Yes	Yes	Yes	Yes	Yes	Yes	Yes	Unclear
Study 22	Sugisaki 2024	Yes	Yes	Yes	Yes	Yes	Yes	Yes	Yes
Study 26	Seki K 2023	Yes	Yes	Unclear	Yes	Yes	Yes	Yes	Yes
Study 35	Liu 1998	Yes	Yes	Unclear	Yes	Yes	Yes	Yes	Yes
Study 56	Micheletti 2022	Yes	Yes	Yes	Yes	Yes	Unclear	Unclear	Yes
Study 59	Naik 2009	Yes	Yes	Yes	Yes	Yes	Yes	Unclear	Yes
Study 66	Gallay 2013	Yes	Yes	Yes	Yes	Yes	Yes	Unclear	Yes

S5B. STORMS-informed reporting assessment

S5B STORMS item legend and status definitions

The panel includes 13 core community-level studies and 18 STORMS items. Item legend: Item 1: sampling site; Item 2: specimen type; Item 3: specimen collection method; Item 4: specimen storage; Item 5: DNA extraction method; Item 6: sequencing region; Item 7: sequencing platform; Item 8: bioinformatics pipeline; Item 9: taxonomic database; Item 10: alpha-diversity method; Item 11: beta-diversity method; Item 12: differential-abundance method; Item 13: contamination control; Item 14: negative control; Item 15: sequencing depth/read counts; Item 16: normalization/rarefaction; Item 17: data availability; Item 18: author-reported limitations.

Status definitions: Reported = explicitly reported; Not reported = not reported; Unclear = insufficiently clear; NA = not applicable. STORMS was used as a reporting assessment and not as a risk-of-bias tool; no total score or overall reporting grade was calculated.

STORMS summary: Reported 175; Not reported 45; Unclear 13; NA 1; total 234.

Study	Author–year	Item 1	Item 2	Item 3	Item 4	Item 5	Item 6	Item 7	Item 8	Item 9	Item 10	Item 11	Item 12	Item 13	Item 14	Item 15	Item 16	Item 17	Item 18

Study	Author–year	Item 1	Item 2	Item 3	Item 4	Item 5	Item 6	Item 7	Item 8	Item 9	Item 10	Item 11	Item 12	Item 13	Item 14	Item 15	Item 16	Item 17	Item 18
Study 7	Yahara H 2020	Reported	Reported	Reported	Reported	Reported	NA	Reported	Reported	Reported	Not reported	Not reported	Not reported	Not reported	Not reported	Reported	Reported	Reported	Reported
Study 10	Badros AZ 2021	Reported	Reported	Reported	Reported	Reported	Unclear	Not reported	Reported	Reported	Reported	Reported	Reported	Not reported	Not reported	Not reported	Not reported	Reported	Reported
Study 37	Ji 2012	Reported	Reported	Reported	Reported	Reported	Reported	Reported	Reported	Reported	Reported	Unclear	Reported	Reported	Not reported	Reported	Reported	Not reported	Reported
Study 38	Kim HY 2024	Reported	Reported	Reported	Reported	Reported	Reported	Reported	Reported	Unclear	Reported	Reported	Not reported	Reported	Not reported	Not reported	Reported	Not reported	Reported
Study 39	Li Q 2020	Reported	Reported	Reported	Not reported	Unclear	Reported	Reported	Reported	Reported	Reported	Reported	Reported	Reported	Reported	Reported	Not reported	Not reported	Not reported
Study 41	Choi 2025	Reported	Reported	Reported	Reported	Unclear	Reported	Reported	Reported	Reported	Reported	Reported	Reported	Reported	Not reported	Not reported	Not reported	Reported	Reported
Study 43	Sedghizadeh 2012	Reported	Reported	Reported	Reported	Reported	Reported	Reported	Reported	Reported	Reported	Unclear	Not reported	Reported	Not reported	Reported	Not reported	Not reported	Reported
Study 44	Pushalkar 2014	Reported	Reported	Reported	Reported	Reported	Reported	Reported	Unclear	Reported	Reported	Unclear	Reported	Reported	Not reported	Reported	Reported	Not reported	Reported
Study 45	Kalyan 2015	Reported	Reported	Unclear	Reported	Reported	Reported	Reported	Reported	Reported	Reported	Reported	Not reported	Not reported	Not reported	Reported	Not reported	Not reported	Not reported
Study 46	Wei 2012	Reported	Reported	Reported	Reported	Reported	Reported	Reported	Reported	Reported	Reported	Unclear	Not reported	Reported	Not reported	Reported	Reported	Not reported	Not reported
Study 47	Erovigni FM 2026	Reported	Reported	Reported	Reported	Reported	Reported	Reported	Reported	Unclear	Reported	Reported	Reported	Not reported	Not reported	Reported	Reported	Reported	Reported

Study	Author–year	Item 1	Item 2	Item 3	Item 4	Item 5	Item 6	Item 7	Item 8	Item 9	Item 10	Item 11	Item 12	Item 13	Item 14	Item 15	Item 16	Item 17	Item 18
Study 48	Kövér Z 2025	Reported	Reported	Reported	Reported	Reported	Reported	Reported	Reported	Reported	Reported	Reported	Reported	Reported	Reported	Reported	Not reported	Reported	Reported
Study 49	Hallmer 2017	Reported	Reported	Reported	Reported	Unclear	Reported	Reported	Reported	Reported	Reported	Unclear	Not reported	Reported	Not reported	Reported	Reported	Not reported	Reported

Abbreviations: JBI, Joanna Briggs Institute; MRONJ, medication-related osteonecrosis of the jaw; NA, not applicable; ORN, osteoradionecrosis; STORMS, Strengthening the Organization and Reporting of Microbiome Studies.

Supplementary Table S6. Study-level microbial evidence matrix

S6A. Core community-level evidence

Study	Author–year	Country	Specimen	Method	Comparator structure	Alpha-diversity metric(s)	Explicit alpha-diversity comparison	Beta-diversity analysis	Differential-abundance/community-composition result
Study 7	Yahara H 2020	Japan	Unstimulated saliva; 1 mL for newly collected samples; publicly available healthy-control metagenomes were also included.	Shotgun metagenomic sequencing	Initial comparison: 5 MRONJ metagenomic datasets and 5 healthy-control metagenomic datasets; extended analysis: 10 MRONJ and 14 healthy-control datasets.	NR	No explicit MRONJ-versus-comparator alpha analysis	NR	Comparative metagenomic analysis reported a 10.1% higher average relative abundance of Actinomyces in MRONJ saliva than in healthy controls.
Study 10	Badros AZ 2021	United States	Whole unstimulated saliva; serial sampling	16S rRNA gene sequencing; 97% OTU clustering; Greengenes, SILVA and HOMD references	BRONJ 14; controls 28 (microbiome profiling)	NR	No explicit MRONJ-versus-comparator alpha analysis	NR	A distinct Streptococcus pattern was reported during follow-up.
Study 37	Ji 2012	United States	soft tissue associated with BRONJ lesion	DGGE and Sanger clone sequencing; V4–V5	20; 10 antibiotic-treated vs 10 non-antibiotic BRONJ; no healthy comparator	Shannon	No explicit MRONJ-versus-comparator alpha analysis	NR	Taxon-pattern differences were reported between antibiotic-treated and untreated BRONJ groups.

Study	Author–year	Country	Specimen	Method	Comparator structure	Alpha-diversity metric(s)	Explicit alpha-diversity comparison	Beta-diversity analysis	Differential-abundance/community-composition result
Study 38	Kim HY 2024	South Korea	oral swab from affected and unaffected sides; submucosal/exudate around lesion	16S rRNA sequencing; V3–V4; Illumina MiSeq; 2 × 300 bp	12; 12 unaffected-site paired samples	NR	No explicit MRONJ-versus-comparator alpha analysis	NR	Community-composition differences were reported between affected and unaffected oral sites.
Study 39	Li Q 2020	China	fresh inflammatory tissue next to necrotic bone or sequestrum	Mixed 16S sequencing and culture methods; V3–V4; Illumina MiSeq, paired-end 250 bp	9; 9 healthy/control oral mucosa specimens	Chao1; Shannon; Simpson; evenness; observed species	MRONJ versus healthy controls	Weighted and unweighted UniFrac; PCoA; PERMANOVA	Selected taxa differed between MRONJ and controls; inflammatory-marker correlations were reported.
Study 41	Choi 2025	South Korea	sequestrum / MRONJ-related oral microbial samples	16S rRNA sequencing; Illumina HiSeq 2500	39 MRONJ; 45 healthy patients	Chao1; Shannon	MRONJ versus healthy controls	Bray–Curtis; PCoA; PERMANOVA	Differential features and a microbial network signal were reported between MRONJ and healthy controls.

Study	Author–year	Country	Specimen	Method	Comparator structure	Alpha-diversity metric(s)	Explicit alpha-diversity comparison	Beta-diversity analysis	Differential-abundance/community-composition result
Study 43	Sedghizadeh 2012	United States	saliva and saliva-induced biofilms	16S rRNA V3–V5 amplicon sequencing; 454 GS FLX Titanium pyrosequencing; separate viral metagenomic component	5 BRONJ; 5 non-BRONJ controls	NR	No explicit MRONJ-versus-comparator alpha analysis	NR	Relative composition was reported as dominated by Proteobacteria, Firmicutes and Actinobacteria; viral abundance and prophage induction were also reported.
Study 44	Pushalkar 2014	United States	BRONJ lesion tissue specimens	16S rRNA sequencing; DGGE and clone sequencing	15 BRONJ; 10 periodontal controls and 5 bisphosphonate-exposed without ONJ	ACE; Chao1; Shannon; Simpson; evenness; richness (unspecified)	MRONJ versus drug-exposed non-MRONJ and periodontal controls	NR	Community-composition and anaerobic-genera differences were reported across groups.
Study 45	Kalyan 2015	Germany	oral microbiome samples; exact oral site unclear	16S rRNA sequencing; 454 pyrosequencing	BAONJ subgroup size not clearly extractable in text; participants exposed to nitrogen-containing bisphosphonates without ONJ and treatment-naive controls	Chao1; Shannon; Simpson; evenness	MRONJ/BAONJ versus drug-exposed without ONJ and treatment-naive controls	Bray–Curtis; Jaccard; PERMANOVA	Community-composition differences were reported across BAONJ, bisphosphonate-exposed non-ONJ, and treatment-naive groups.

Study	Author–year	Country	Specimen	Method	Comparator structure	Alpha-diversity metric(s)	Explicit alpha-diversity comparison	Beta-diversity analysis	Differential-abundance/community-composition result
Study 46	Wei 2012	United States	jawbone samples	16S rRNA molecular profiling; DGGE and clone sequencing	6 BRONJ; 6 cancer controls without BRONJ or bisphosphonate exposure but with infection	ACE; Chao1; Shannon; Simpson; evenness; richness (unspecified)	MRONJ versus cancer controls	NR	Molecular community profiles differed between BRONJ and cancer controls.
Study 47	Erovigni FM 2026	Italy	saliva	16S rRNA sequencing; multiple amplicon regions; microbiome panel/NGS	18 MRONJ cases; 21 drug-exposed controls without MRONJ; 16 healthy subjects	NR	No explicit MRONJ-versus-comparator alpha analysis	NR	Salivary community composition differed among healthy, drug-exposed control and MRONJ groups.
Study 48	Kövér Z 2025	Hungary	surgically removed bone samples	Mixed 16S sequencing and culture methods; V3–V4; Illumina MiSeq	35 MRONJ; 35 healthy extraction controls; 5+5 selected for 16S	Chao1; richness (unspecified)	MRONJ versus healthy extraction controls	PERMANOVA	Community-composition differences were reported between MRONJ and healthy extraction controls.
Study 49	Hallmer 2017	Sweden	necrotic bone center and visually healthy bone	16S rRNA molecular profiling; V3–V5; 454 GS Junior pyrosequencing	18 necrotic bone samples; 11 visually healthy bone samples plus 2 healthy controls	Chao1; Shannon; phylogenetic distance	No explicit MRONJ-versus-comparator alpha analysis	NR	No separate differential-abundance comparison was retained.

S6B. Targeted MRONJ microbiological evidence

Study	Author–year	Specimen	Method	Explicit Actinomyces event/denominator available	Direct biofilm evidence	Strict-anaerobe evidence	Fungal evidence	Viral evidence	Actinomyces denominator unit
Study 1	Yamashita 2012	NR	EBER in situ hybridization					Yes	NR
Study 2	Kimoto 2013	NR	EBER in situ hybridization					Yes	NR
Study 3	Miyamoto 2017	NR	EBER in situ hybridization					Yes	NR
Study 6	Kanno NI 2024	necrotic bone/sequestrum	EBER in situ hybridization and EBV immunohistochemistry					Yes	NR
Study 9	Jabbour Z 2016	necrotic bone/sequestrum	DNA checkerboard hybridization with bacterial and fungal genomic probes				Yes		NR
Study 20	Fukuzawa S 2022	palatal mucosal biopsy and sequestrum	EBER in situ hybridization					Yes	NR
Study 22	Sugisaki 2024	maxillary gingival biopsy and resected necrotic bone	EBER in situ hybridization					Yes	NR
Study 24	He P 2022	wound/tissue specimens	Culture-based microbiological testing			Yes	Yes		NR
Study 25	Zirk M 2019	osseous lesion-bottom and submucosal specimens	Culture-based microbiological testing			Yes			NR

Study	Author–year	Specimen	Method	Explicit Actinomyces event/denominator available	Direct biofilm evidence	Strict-anaerobe evidence	Fungal evidence	Viral evidence	Actinomyces denominator unit
Study 37	Ji 2012	soft tissue associated with BRONJ lesion	DGGE and Sanger clone sequencing; V4–V5			Yes			NR
Study 38	Kim HY 2024	oral swab from affected and unaffected sides; submucosal/exudate around lesion	16S rRNA sequencing; V3–V4; Illumina MiSeq; 2 × 300 bp			Yes			NR
Study 39	Li Q 2020	fresh inflammatory tissue next to necrotic bone or sequestrum	Mixed 16S sequencing and culture methods; V3–V4; Illumina MiSeq, paired-end 250 bp			Yes			NR
Study 41	Choi 2025	sequestrum / MRONJ-related oral microbial samples	16S rRNA sequencing; Illumina HiSeq 2500			Yes			NR
Study 42	Hershberger 2024	MRONJ tissue culture specimens	Culture-based microbiological testing			Yes			NR
Study 43	Sedghizadeh 2012	saliva and saliva-induced biofilms	16S rRNA V3–V5 amplicon sequencing; 454 GS FLX Titanium pyrosequencing; separate viral metagenomic component		Yes			Yes	NR

Study	Author–year	Specimen	Method	Explicit Actinomyces event/denominator available	Direct biofilm evidence	Strict-anaerobe evidence	Fungal evidence	Viral evidence	Actinomyces denominator unit
Study 44	Pushalkar 2014	BRONJ lesion tissue specimens	16S rRNA sequencing; DGGE and clone sequencing			Yes			NR
Study 45	Kalyan 2015	oral microbiome samples; exact oral site unclear	16S rRNA sequencing; 454 pyrosequencing			Yes			NR
Study 46	Wei 2012	jawbone samples	16S rRNA molecular profiling; DGGE and clone sequencing			Yes			NR
Study 47	Erovigni FM 2026	saliva	16S rRNA sequencing; multiple amplicon regions; microbiome panel/NGS			Yes			NR
Study 48	Kövér Z 2025	surgically removed bone samples	Mixed 16S sequencing and culture methods; V3–V4; Illumina MiSeq	Yes		Yes			patients
Study 49	Hallmer 2017	necrotic bone center and visually healthy bone	16S rRNA molecular profiling; V3–V5; 454 GS Junior pyrosequencing			Yes			NR

Study	Author–year	Specimen	Method	Explicit Actinomyces event/denominator available	Direct biofilm evidence	Strict-anaerobe evidence	Fungal evidence	Viral evidence	Actinomyces denominator unit
Study 50	De Bruyn 2018	swabs at infected MRONJ lesion margin with visible pus	Targeted real-time PCR using TaqMan assays			Yes			NR
Study 51	Panya 2017	MRONJ bone samples	Culture and PCR/qPCR	Yes					specimens
Study 52	Kövér Z 2023	necrotic bone samples	Anaerobic culture and molecular identification			Yes			NR
Study 53	Ewald 2021	necrotic bone	Culture and PCR/qPCR			Yes			NR
Study 54	Kusumoto 2025	deep tissue culture specimens	Culture-based microbiological testing			Yes			NR
Study 55	Russmueller 2016	deep biopsies of necrotic bone	Culture and histopathology with special stains	Yes		Yes			patients
Study 56	Micheletti 2022	NR	BSE-SEM/SE-SEM and resin-cast etching		Yes				NR
Study 60	Hansen et al. 2006/2007 (MRONJ cluster)	necrotic jaw bone tissue	Histomorphological and histopathological assessment; PCR on selected tissue specimens in the 2007 companion report	Yes					patients

Study	Author–year	Specimen	Method	Explicit Actinomyces event/denominator available	Direct biofilm evidence	Strict-anaerobe evidence	Fungal evidence	Viral evidence	Actinomyces denominator unit
Study 62	Sedghizadeh 2008	affected bone samples; NR	Scanning electron microscopy and biofilm morphology		Yes	Yes			NR
Study 63	Santana DA 2025	histologic slides / jaw lesion tissue	Histopathology with special stains	Yes					patients
Study 65	Brody 2022	histological MRONJ jaw samples	Histopathology with special stains	Yes					specimens

Yes indicates that the study met the predefined criterion for that evidence domain.

Blank cells indicate that the study did not meet the predefined criterion for that evidence domain; blanks do not necessarily indicate that the domain was not assessed.

The *Actinomyces* event/denominator column identifies only the subset of studies with explicit MRONJ-specific events and denominators and does not represent all studies reporting *Actinomyces* detection.

S6C. Contextual evidence

Study	Author–year	Condition	Specimen	Method	Main finding category
Study 12	Choi SG 2022	Jaw osteomyelitis	necrotic bone/sequestrum	Culture; histopathology/special staining	Mixed microbiological methods
Study 13	Li 2023	Osteoradionecrosis	necrotic bone/sequestrum	Community sequencing; culture; PCR/qPCR	Mixed microbiological methods
Study 14	Takeda 2021	Osteoradionecrosis	necrotic bone/sequestrum	Culture; PCR/qPCR; histopathology/special staining	Mixed microbiological methods
Study 16	Bhavthankar JD 2023	Jaw osteomyelitis	necrotic bone/sequestrum	Culture	Culture-based microbiological findings
Study 17	Sezer B 2017	Jaw osteomyelitis	necrotic bone/sequestrum	Culture; PCR/qPCR; histopathology/special staining	Mixed microbiological methods

Study	Author–year	Condition	Specimen	Method	Main finding category
Study 27	Arianpour K 2024	Osteoradionecrosis	tissue at rescue-flap surgery	Tissue culture; Gram staining	Mixed microbiological methods
Study 28	Longfellow GA 2026	Osteoradionecrosis	clinical osteoradionecrosis cultures	Clinical culture; antimicrobial-resistance analysis	Culture-based microbiological findings
Study 29	Hansen et al. 2006 (ORN cluster)	Osteoradionecrosis	bone and fistula skin/mucosa biopsies; mandibular bone biopsy	Histopathology with special stains; semi-nested PCR	Mixed microbiological methods
Study 30	Zhu 2022	Osteoradionecrosis	jaw lesion specimens	Culture; antimicrobial-susceptibility testing	Culture-based microbiological findings
Study 31	Curi MM 2000	Osteoradionecrosis	jawbone biopsy	Bone-biopsy histopathology	Histological or microscopic microbial detection
Study 32	Pigrau C 2009	Jaw osteomyelitis	jaw infection cultures	Clinical culture; antimicrobial-susceptibility testing	Culture-based microbiological findings
Study 33	Frid P 2009	Jaw osteomyelitis	mandibular bone biopsy	Culture; PCR	Mixed microbiological methods
Study 34	Bertrand K 2018	Jaw osteomyelitis	intraoral bone samples	Intraoral bone culture	Culture-based microbiological findings
Study 35	Liu 1998	Osteoradionecrosis	necrotic maxillary bone fragments	Microscopic examination of necrotic bone	Histological or microscopic microbial detection
Study 40	Goda 2014	Jaw osteomyelitis	bone samples from chronic osteomyelitis jaw lesions	Community-level sequencing	Community-level profiling
Study 67	Gaetti-Jardim Júnior 2010	Jaw osteomyelitis	bone sequestra; purulent secretion; granulomatous tissue biopsies	Anaerobic culture; PCR	Mixed microbiological methods

Abbreviations: ACE, abundance-based coverage estimator; BAONJ, bisphosphonate-associated osteonecrosis of the jaw; BRONJ, bisphosphonate-related osteonecrosis of the jaw; BSE-SEM, backscattered-electron scanning electron microscopy; DGGE, denaturing gradient gel electrophoresis; EBV, Epstein–Barr virus; EBER, Epstein–Barr virus-encoded RNA; HOMD, Human Oral Microbiome Database; MRONJ, medication-related osteonecrosis of the jaw; NGS, next-generation sequencing; NR, not reported; ONJ, osteonecrosis of the jaw; ORN, osteoradionecrosis; OTU, operational taxonomic unit; PCoA, principal coordinates analysis; PCR, polymerase chain reaction; PERMANOVA, permutational multivariate analysis of variance; qPCR, quantitative polymerase chain reaction; SE-SEM, secondary-electron scanning electron microscopy.
