# Supplementary material for: Oral microbiome and lesion-associated microbiological findings in medication-related osteonecrosis of the jaw: a systematic review and evidence map
Source: Front Cell Infect Microbiol. 2026 Sep 8;16:1923000. doi: 10.3389/fcimb.2026.1923000 (PMC13593453; doi:10.3389/fcimb.2026.1923000)
Supplement: Supplementary file 2 [file Table1.docx]

Table 1. Characteristics of the core community-level MRONJ studies

| **Study, country and design** | **MRONJ and comparator sample sizes** | **Diagnostic definition** | **Indication and medication exposure** | **MRONJ stage** | **Antibiotic exposure** | **Specimen and sampling** | **Community-level method** |
| --- | --- | --- | --- | --- | --- | --- | --- |
| Yahara H 2020; Japan; Analytical cross-sectional | Initial comparison: 5 MRONJ metagenomic datasets and 5 healthy-control metagenomic datasets; extended analysis: 10 MRONJ and 14 healthy-control datasets. | NR | Cancer-associated MRONJ; antiresorptive exposure; route NR | NR | NR | Unstimulated saliva; 1 mL | Shotgun metagenomic sequencing; Illumina HiSeq 2 × 150 bp |
| Badros AZ 2021; United States; Cohort | 14 patients with BRONJ; 28 control participants (microbiome profiling) | NR | Multiple myeloma; bisphosphonate therapy; route NR | NR | NR | Whole unstimulated saliva; serial sampling | 16S rRNA gene sequencing; 97% OTU clustering |
| Ji 2012; United States; Analytical cross-sectional | 20 patients; 10 antibiotic-treated versus 10 non-antibiotic BRONJ patients; no healthy comparator | historical BRONJ definition | Bisphosphonate exposure; intravenous (n=14) and oral (n=6) | Stage 0–2 | 10 received systemic antibiotics; 10 had no prior antibiotics; treated patients were sampled during a 2-week course | soft tissue associated with BRONJ lesion | 16S rRNA DGGE fingerprinting with clone-library sequencing (ABI PRISM 3730xl) |
| Kim HY 2024; South Korea; Analytical cross-sectional | 12 patients; 12 unaffected-site paired samples | partial definition | Osteoporosis (n=11) and multiple myeloma (n=1); medication class/route NR | NR | No antibiotics within 1 week before sampling | oral swab from affected and unaffected sides; submucosal/exudate around lesion | 16S rRNA sequencing; V3–V4; Illumina MiSeq; 2 × 300 bp |
| Li Q 2020; China; Case-control | 9 patients; 9 healthy/control oral mucosa specimens | NR | Osteoporosis (n=1) and cancer (n=8); medication class/route NR | Stage II/III | No antibiotics within 1 month before sampling | fresh inflammatory tissue next to necrotic bone or sequestrum | Mixed 16S sequencing and culture methods; V3–V4; Illumina MiSeq, paired-end 250 bp |
| Choi 2025; South Korea; Case-control | 39 patients with MRONJ; 45 healthy participants | NR | Osteoporosis; bisphosphonate and denosumab; route partly reported | NR | NR | sequestrum / MRONJ-related oral microbial samples | 16S rRNA sequencing; Illumina HiSeq 2500 |
| Sedghizadeh 2012; United States; Case-control | 5 patients with BRONJ; 5 non-BRONJ control participants | historical BRONJ definition | Osteoporosis and prostate cancer; oral or intravenous bisphosphonate exposure | NR | No local or systemic antimicrobials within 6 months before sampling | saliva and saliva-induced biofilms | 16S rRNA V3–V5 amplicon sequencing; 454 GS FLX Titanium pyrosequencing; separate viral metagenomic component |
| Pushalkar 2014; United States; Case-control | 15 patients with BRONJ; 10 periodontal control participants and 5 bisphosphonate-exposed participants without ONJ | historical BRONJ definition | Cancer; bisphosphonate exposure; route NR | NR | No antibiotics for approximately 3 months before sampling | BRONJ lesion tissue specimens | 16S rRNA DGGE fingerprinting with clone-library sequencing |
| Kalyan 2015; Germany; Analytical cross-sectional | BAONJ subgroup size not clearly extractable; participants exposed to nitrogen-containing bisphosphonates without ONJ and treatment-naive controls | NR | Bisphosphonate exposure; indication and route NR | NR | No active antibiotic treatment at sampling | oral microbiome samples; exact oral site unclear | 16S rRNA sequencing; 454 pyrosequencing |
| Wei 2012; United States; Case-control | 6 patients with BRONJ; 6 cancer control participants without BRONJ or bisphosphonate exposure but with infection | NR | Cancer; intravenous bisphosphonate exposure | NR | No antibiotics at the time of sampling | jawbone samples | 16S rRNA DGGE fingerprinting with clone-library sequencing |
| Erovigni FM 2026; Italy; Case-control | 18 patients with MRONJ; 21 drug-exposed control participants without MRONJ; 16 healthy participants | NR | Mixed indications; bisphosphonate and denosumab exposure | NR | Antibiotics started 2 days before surgical sampling | saliva | 16S rRNA sequencing; multiple amplicon regions; microbiome panel/NGS |
| Kövér Z 2025; Hungary; Case-control | 35 patients with MRONJ; 35 healthy extraction controls; 5 + 5 specimens selected for 16S | partial definition | Malignancy with bone metastases; bisphosphonate/denosumab; predominantly intravenous | NR | No antibiotics for at least 2 months before primary sampling; initiated subsequently | surgically removed bone samples | Mixed 16S sequencing and culture methods; V3–V4; Illumina MiSeq |
| Hallmer 2017; Sweden; Analytical cross-sectional | 18 necrotic bone samples; 11 visually healthy bone samples; 2 healthy control participants | NR | Mixed indications; medication exposure NR | NR | No antibiotics within 3 months before study sampling | necrotic bone center and visually healthy bone | 16S rRNA molecular profiling; V3–V5; 454 GS Junior pyrosequencing |

Abbreviations: BAONJ, bisphosphonate-associated osteonecrosis of the jaw; BRONJ, bisphosphonate-related osteonecrosis of the jaw; DGGE, denaturing gradient gel electrophoresis; MRONJ, medication-related osteonecrosis of the jaw; NGS, next-generation sequencing; NR, not reported; ONJ, osteonecrosis of the jaw; OTU, operational taxonomic unit.
