# Supplementary material for: Oral microbiome and lesion-associated microbiological findings in medication-related osteonecrosis of the jaw: a systematic review and evidence map
Source: Front Cell Infect Microbiol. 2026 Sep 8;16:1923000. doi: 10.3389/fcimb.2026.1923000 (PMC13593453; doi:10.3389/fcimb.2026.1923000)
Supplement: Supplementary file 3 [file Table2.docx]

Table 2. Reporting of key clinical and sampling variables across the MRONJ studies

| **Variable** | **Reported, n/51 (%)** | **Not reported, n/51 (%)** | **Unclear, n/51 (%)** |
| --- | --- | --- | --- |
| Diagnostic definition | 24/51 (47.1%) | 23/51 (45.1%) | 4/51 (7.8%) |
| Exposed/probeable-bone duration | 12/51 (23.5%) | 37/51 (72.5%) | 2/51 (3.9%) |
| Exclusion of previous jaw radiotherapy | 6/51 (11.8%) | 43/51 (84.3%) | 2/51 (3.9%) |
| Exclusion of metastatic disease to the jaw | 1/51 (2.0%) | 48/51 (94.1%) | 2/51 (3.9%) |
| Underlying indication | 42/51 (82.4%) | 9/51 (17.6%) | 0/51 (0.0%) |
| Medication class | 42/51 (82.4%) | 9/51 (17.6%) | 0/51 (0.0%) |
| Medication route | 42/51 (82.4%) | 9/51 (17.6%) | 0/51 (0.0%) |
| Treatment duration | 16/51 (31.4%) | 35/51 (68.6%) | 0/51 (0.0%) |
| MRONJ stage | 26/51 (51.0%) | 25/51 (49.0%) | 0/51 (0.0%) |
| Lesion location | 43/51 (84.3%) | 8/51 (15.7%) | 0/51 (0.0%) |
| Recent antibiotic exposure | 28/51 (54.9%) | 23/51 (45.1%) | 0/51 (0.0%) |
| Antibiotic timing | 21/51 (41.2%) | 30/51 (58.8%) | 0/51 (0.0%) |
| Oral hygiene | 12/51 (23.5%) | 39/51 (76.5%) | 0/51 (0.0%) |
| Periodontal disease | 21/51 (41.2%) | 30/51 (58.8%) | 0/51 (0.0%) |
| Prior extraction or dental surgery | 41/51 (80.4%) | 10/51 (19.6%) | 0/51 (0.0%) |
| Prior debridement | 17/51 (33.3%) | 34/51 (66.7%) | 0/51 (0.0%) |
| Smoking | 15/51 (29.4%) | 36/51 (70.6%) | 0/51 (0.0%) |
| Immunosuppression | 23/51 (45.1%) | 28/51 (54.9%) | 0/51 (0.0%) |
| Specimen timing | 23/51 (45.1%) | 28/51 (54.9%) | 0/51 (0.0%) |
| Sampling site | 43/51 (84.3%) | 8/51 (15.7%) | 0/51 (0.0%) |
