# Supplementary material for: Oral microbiome and lesion-associated microbiological findings in medication-related osteonecrosis of the jaw: a systematic review and evidence map
Source: Front Cell Infect Microbiol. 2026 Sep 8;16:1923000. doi: 10.3389/fcimb.2026.1923000 (PMC13593453; doi:10.3389/fcimb.2026.1923000)
Supplement: Supplementary file 4 [file Table3.docx]

Table 3. Summary of microbiological evidence and quantitative-synthesis feasibility

| **Evidence domain** | **Independent studies or result-level observations** | **Evidence type** | **Main descriptive finding** | **Principal compatibility limitation** | **Synthesis approach** |
| --- | --- | --- | --- | --- | --- |
| Community-level microbiome profiling | 13 independent studies | Community-level profiles | Community profiles were reported with study-specific specimens and methods. | Design, specimen, comparator, and outcome heterogeneity. | Narrative synthesis |
| Alpha diversity | 8 studies; 6 with explicit MRONJ-versus-comparator analyses | Richness and diversity/evenness metrics | Findings were retained separately by metric, comparator structure, and sampling design. | Metrics, paired status, summaries, and comparators were incompatible. | Not quantitatively pooled |
| *Actinomyces* | 9 result-level observations from 6 independent studies | Method-specific event/denominator data | Method-specific event/denominator findings were summarized separately by detection method and denominator unit. | Small strata; mixed patient/specimen units; selected and targeted methods. | Method-specific descriptive display |
| Direct biofilm | 3 independent studies | Direct or validated biofilm evidence | Direct biofilm evidence was distinguished from presumed or discussion-only biofilm. | Visualization and validation methods were not uniform. | Evidence-map synthesis |
| Strict anaerobic evidence | 19 independent MRONJ studies | Direct anaerobic evidence or named obligate-anaerobe findings | Strict evidence required a qualifying anaerobic culture, explicit author classification, or named obligate anaerobes. | Culture and molecular evidence were method-specific. | Evidence-map synthesis |
| Fungal evidence | 2 independent studies | Explicit fungal finding | Fungal findings were reported separately from bacterial and viral observations. | Limited and heterogeneous study-level reporting. | Evidence-map synthesis |
| Viral evidence | 7 independent studies | Explicit viral finding | Viral findings were retained as explicit result-level evidence. | Limited target-specific and case-based observations. | Evidence-map synthesis |
| Contextual jaw-disease evidence | 16 independent studies | Contextual community-level or lesion-associated evidence | Contextual jaw-disease findings were separated from MRONJ-specific synthesis. | Different diseases and clinical ecologies; excluded from MRONJ-specific counts. | Narrative synthesis |

MRONJ, medication-related osteonecrosis of the jaw.
